# Dating of a large tool assemblage at the Cooper’s Ferry site (Idaho, USA) to ~15,785 cal yr B.P. extends the age of stemmed points in the Americas

**DOI:** 10.1126/sciadv.ade1248

**Published:** 2022-12-23

**Authors:** Loren G. Davis, David B. Madsen, David A. Sisson, Lorena Becerra-Valdivia, Thomas Higham, Daniel Stueber, Daniel W. Bean, Alexander J. Nyers, Amanda Carroll, Christina Ryder, Matt Sponheimer, Masami Izuho, Fumie Iizuka, Guoqiang Li, Clinton W. Epps, F. Kirk Halford

**Affiliations:** ^1^ Department of Anthropology, Oregon State University, 203 Waldo Hall, Corvallis, OR 97331, USA.; ^2^ Department of Anthropology, University of Nevada-Reno, 512 Ansari, Reno, NV 89557, USA.; ^3^ Bureau of Land Management, Cottonwood Field Office, 2 Butte Drive, Cottonwood, ID 83522, USA.; ^4^ Oxford Radiocarbo​n Accelerator Unit, School of Archaeology, 1 South Parks Road, Oxford OX1 3TG, UK.; ^5^ Department of Evolutionary Anthropology, University of Vienna, Djerassiplatz 1, 1030 Wien, Austria.; ^6^ Human Evolution and Archaeological Sciences (HEAS), University of Vienna, A-1030 Vienna, Austria.; ^7^ Department of Anthropology, University of Victoria, P.O. Box 1700 STN CSC, Victoria, BC V8W 2Y2, Canada.; ^8^ Northwest Archaeometrics, 5060 SW Philomath Blvd, #331, Corvallis, OR 97333, USA.; ^9^ SWCA Environmental Consultants, 1800 NW Upshur St, Ste. 100, Portland, OR 97209, USA.; ^10^ Department of Anthropology, University of Colorado Boulder, Hale Science Building, 1350 Pleasant St., Boulder, CO 80309, USA.; ^11^ Tokyo Metropolitan University, Faculty of Humanities and Social Sciences, 1-1 Minami-Osawa, Hachioji-shi, Tokyo 192-0397, Japan.; ^12^ Department of Anthropology and Research Reactor Center, University of Missouri, Swallow Hall, 112 S 9th Street, Columbia, MO 65211, USA.; ^13^ Research School of Arid Environment and Climate Change, MOE Key Laboratory of West China’s Environmental System, Lanzhou University, 222 Tianshuinanlu, Lanzhou, Gansu 730000, China.; ^14^ Oregon State University Department of Fisheries, Wildlife, and Conservation Sciences, 104 Nash Hall, Corvallis, OR 97331, USA.; ^15^ Department of Anthropology, Boise State University, Boise, ID 83725, USA.

## Abstract

The timing and character of the Pleistocene peopling of the Americas are measured by the discovery of unequivocal artifacts from well-dated contexts. We report the discovery of a well-dated artifact assemblage containing 14 stemmed projectile points from the Cooper’s Ferry site in western North America, dating to ~16,000 years ago. These stemmed points are several thousand years older than Clovis fluted points (~13,000 cal yr B.P.) and are ~2300 years older than stemmed points found previously at the site. These points date to the end of Marine Isotope Stage 2 when glaciers had closed off an interior land route into the Americas. This assemblage includes an array of stemmed projectile points that resemble pre-Jomon Late Upper Paleolithic tools from the northwestern Pacific Rim dating to ~20,000 to 19,000 years ago, leading us to hypothesize that some of the first technological traditions in the Americas may have originated in the region.

## Teaser

A collection of stemmed projectile points excavated from cultural features dates to ~15,785 cal BP yr at Cooper’s Ferry, Idaho.

## INTRODUCTION

**Fig. 1.**
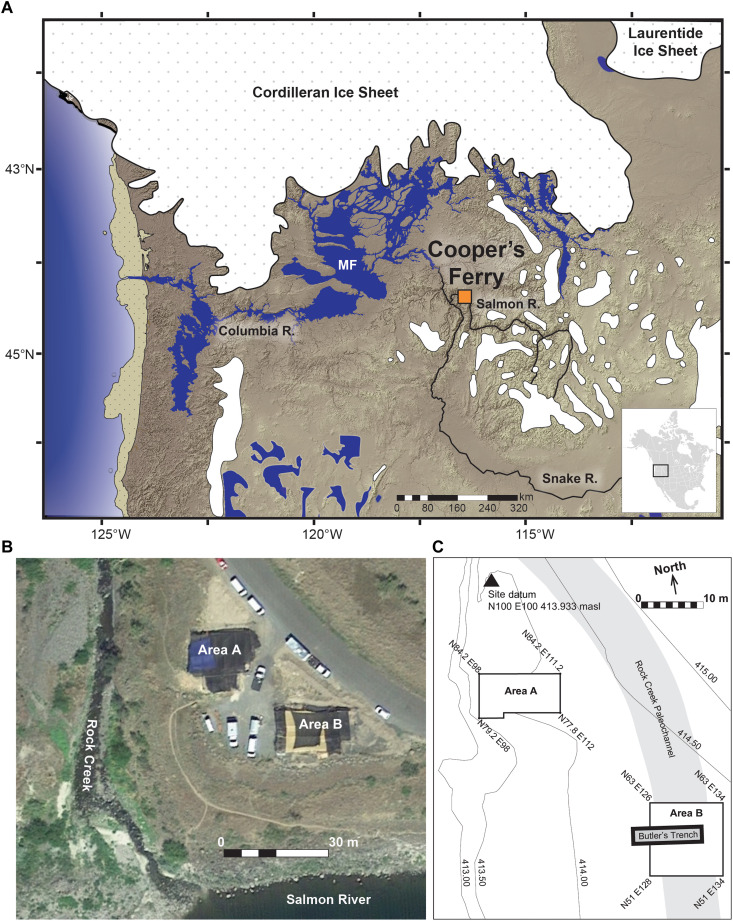
Location maps and aerial images showing the location of the Cooper’s Ferry site and excavation areas. Paleoenvironmental conditions in the Pacific Northwest during glacial conditions at ~16,000 calibrated years before the present (cal yr B.P.) shown in (**A**). Aerial image of the site showing the location of Area A and Area B in relation to the Salmon River (**B**). Site map showing the location of Butler’s Trench and the Rock Creek Paleochannel (**C**). Projected regional environmental aspects at ~16,000 cal yr B.P. are based on modeled extents of Cordilleran and Laurentide glacial ice (*31*), mountain glacier complexes (*32*), positions of Glacial Lake Missoula, Glacial Lake Columbia, the modeled path of the Missoula Flood (MF) and its impoundment pool (*33*), smaller northern Great Basin pluvial lakes (*34*), and shoreline extents along the Pacific outer continental shelf (shown as a tan dotted area at left) (*35*). Aerial image shown in (B) shows excavations in progress on 30 July 2016 (*36*). masl, m above sea level.

**Fig. 2.**
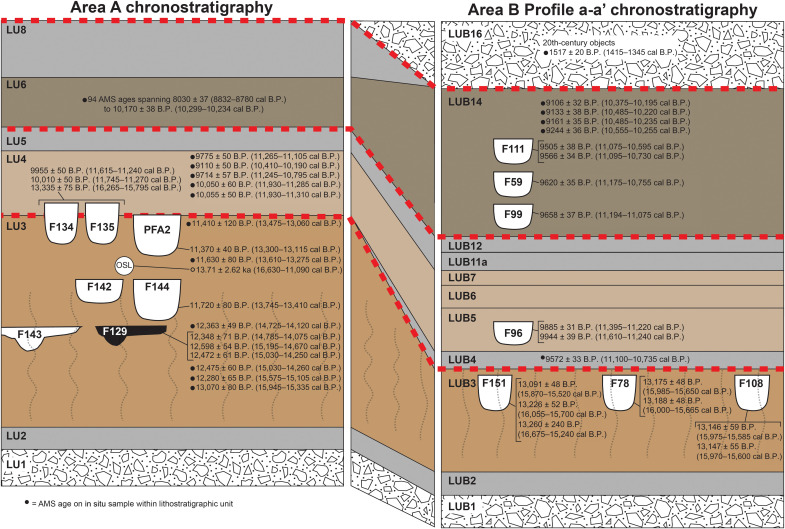
Chronostratigraphic correlation between Area A and Area B. Dashed lines indicate erosional surfaces. Wavy lines show areas of soil development. Black circles indicate radiocarbon ages from samples recovered in a stratigraphic unit. Radiocarbon ages without circles are from cultural features. Radiocarbon ages on LU3 samples derived from rodent burrows (*1*) are not shown. The vertical scale of each composite stratigraphic profile is ~3.0 m. OSL, optically stimulated luminescence. AMS- accelerator mass spectrometry.

Archaeological excavations in Area A at the Cooper’s Ferry site, located on a terrace of the lower Salmon River of western Idaho (Fig. 1), produced a record of a ~16,000- to 13,200-year-old stone tool assemblage that included the earliest known stemmed points in western North America (*1*). This evidence was found in a deeply buried layer of pedogenically altered glacial loess, termed lithostratigraphic unit 3 (LU3) (Fig. 2). LU3 contains a paleosol, called the Rock Creek Soil, which includes a rubified A horizon, calcic B horizon, and loessal C horizon formed roughly in the middle of the 75- to 50-cm-thick LU3 loess deposits in Area A. The Rock Creek Soil has been dated between ~16,450 and 14,160 calibrated years before the present (cal yr B.P.) at multiple localities in the lower Salmon River canyon upstream of the site (*2*–*4*). An erosional unconformity at the top of the unit removed an unknown amount of the LU3 loess and deposits immediately overlying LU3. Four cultural features were found in LU3, including three pits that contained a tooth fragment from an extinct *Equus* sp., numerous bone fragments, flake tools, debitage, and a hearth feature with charcoal radiocarbon dated to ~14,660 cal yr B.P. (Fig. 2: F142, F144, F143, and F129). Radiocarbon dating of culturally associated animal bone and charcoal from the middle portion of LU3 returned ages between ~15,660 and 14,650 cal yr B.P., while Bayesian modeling predicted that LU3 sediment deposition and initial human occupation in Area A began sometime between 16,560 and 15,280 cal yr B.P. [95.4% confidence interval (CI)]. However, while in situ flake tools, debitage, a fire cracked rock(FCR), animal bone fragments, and small pieces of charcoal were also recovered stratigraphically in the lower half of LU3 below the pits and hearth, no formal stone tools, cultural features, or radiocarbon dated samples were recovered from the deeper LU3 sediments, forcing us to rely on Bayesian modeling to estimate that the initial occupation at the site began sometime ~16,000 cal yr B.P. The use of this statistical modeling, together with the limited artifact array from the lowest LU3 loess, has led to some speculation that the artifacts in lower LU3 were present because of stratigraphic mixing and that the site’s earliest cultural remains were not as old as hypothesized (*5*, *6*).

Now, we can report the results of separate excavations conducted at the site’s eastern side in Area B, which revealed additional contemporaneous evidence of early cultural occupation at the site and helps confirm the early age estimates derived from the Area A excavations. We report consistent ^14^C ages from excavated cultural pits originating wholly within the lowest cultural deposits in Area B ranging from 13,260 ± 240 to 13,091 ± 48 yr B.P. (16,675 to 15,240 cal yr B.P. to 15,772 to 15,617 cal yr B.P.). These formal cultural features and the surrounding sediments contain 14 complete and fragmentary stemmed projectile points, other stone tools, substantial amounts of lithic debris, and animal bone fragments. The found projectile points are thousands of years older than Clovis fluted points (~13,000 cal yr B.P.) in North America (*7*, *8*) and are ~2300 years older than stemmed points previously found in Area A (*1*). This evidence greatly extends the timing of stemmed point technology in the Americas. Moreover, unlike several other pre-Clovis age sites in North America (*8*), the tool assemblage from lower LU3 is now quite large, allowing its morphological/technological characteristics to be determined and used in a search for where the antecedents of that technological tradition may be found. We hypothesize that the form of these early stemmed points and the lithic technology used to produce them are similar to bifacial points found in northeast Asia, particularly northern Japan, dating to the Late Upper Paleolithic ~21,400 to 16,170 cal yr B.P. (*9*). Here, we describe the Area B stratigraphy and chronology, characterize this early Cooper’s Ferry stemmed point assemblage, and discuss its implications for understanding the Pleistocene archaeology of the Americas.

## RESULTS

**Fig. 3.**
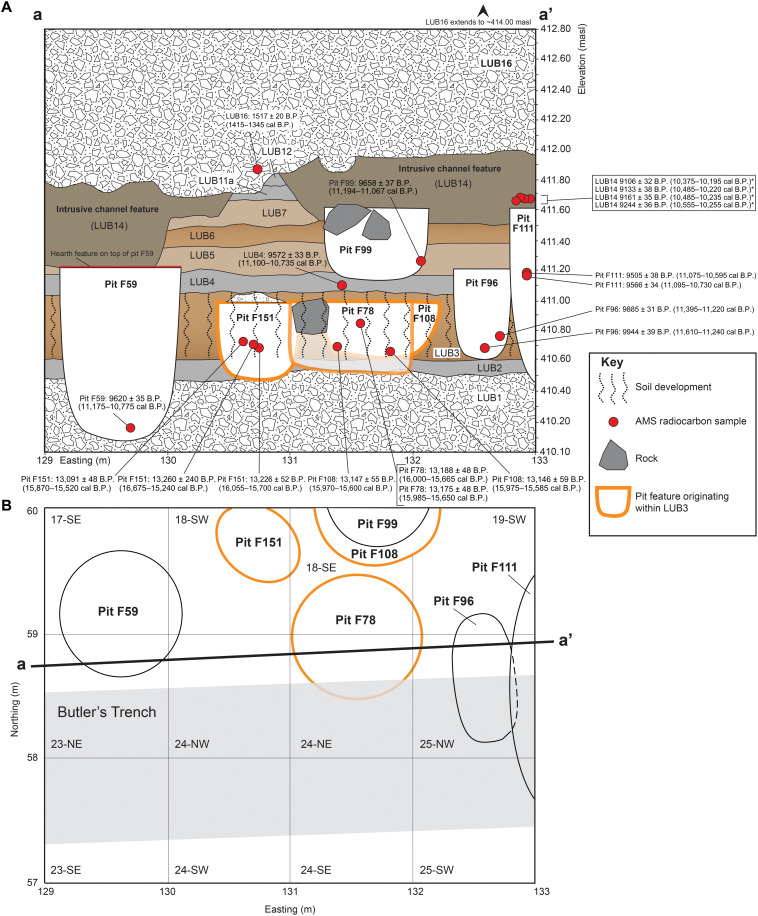
Composite stratigraphy of Area B. Drawing of stratigraphic units exposed along the a-a’ easting profile (**A**). Looking north into the deposits arranged near the a-a’ stratigraphic transect–pits F151 and F108 are positioned behind pit F78. To show this arrangement, the fill of F78 is made partially transparent. Plan view of Area B’s deepest excavation units showing distribution of pit features and trench excavation placed by Butler (**B**). Excavation unit numbers and quadrant designations are shown in each 1 m–by–1 m square (e.g., 23-SE).

Archaeological excavations were conducted at the Cooper’s Ferry site from 2012 to 2018 at a location designated as Area B (Fig. 1 and fig. S1). The base of these excavations exposed a stratified sequence of alluvial gravel and sand deposits (LUB1-LUB2) overlain by loess (LUB3; Figs. 2 and 3 and fig. S2). These LUs are informally designated at the site level. A backhoe trench used to test the sediments in Area B in the 1960s (*10*) partially disturbed some of the deposits in Area B, but given the readily identifiable intact sediments of the LUB3 brown loess, these darker disturbed trench deposits were easily recognized and discarded before excavation.

### Area B stratigraphy

Area B is located closer to both the Salmon River and a paleochannel of the nearby Rock Creek (Fig. 1). As a result, its upper stratigraphy is more complex than that in the adjacent Area A (*1*) because of slightly different erosional and depositional factors related to this proximity. However, the lowest sedimentary units containing the earliest cultural deposits can be readily traced across the site to Area A (Fig. 2). The stratigraphy of Area B includes 11 LUs and an occurrence of the Rock Creek Soil (Figs. 2 and 3 and table S1). LUB3, the equivalent of LU3 in Area A, is a loess that overlies the LUB2 and LUB1 alluvium. The same erosional unconformity at the top of LU3 also occurs at the top of LUB3, but more of the upper loess deposits of this sedimentary unit were removed in Area B than in Area A. As a result, only the lower portion of the LU3 loess–equivalent sediments remains in LUB3.

This erosion also removed the upper portion of the Rock Creek Soil in Area B, and only the calcic B and loessal C horizons remain. This paleosol formed after the construction of the cultural pit features described below as the carbonate horizon extends through the fill of these pits. Bone fragments and stone artifacts in LUB3, including those in the pits, bear heavy carbonate coatings associated with Rock Creek Soil pedogenesis (fig. S3). This carbonate coating distinguishes LUB3 artifacts from the artifacts found in the overlying sediments and indicates that their deposition at the site predates the 16,450 to 14,160 cal yr B.P. formation of the Rock Creek Soil (*2*–*4*, see the supplementary materials).

### Archaeological evidence originating within LUB3

**Fig. 4.**
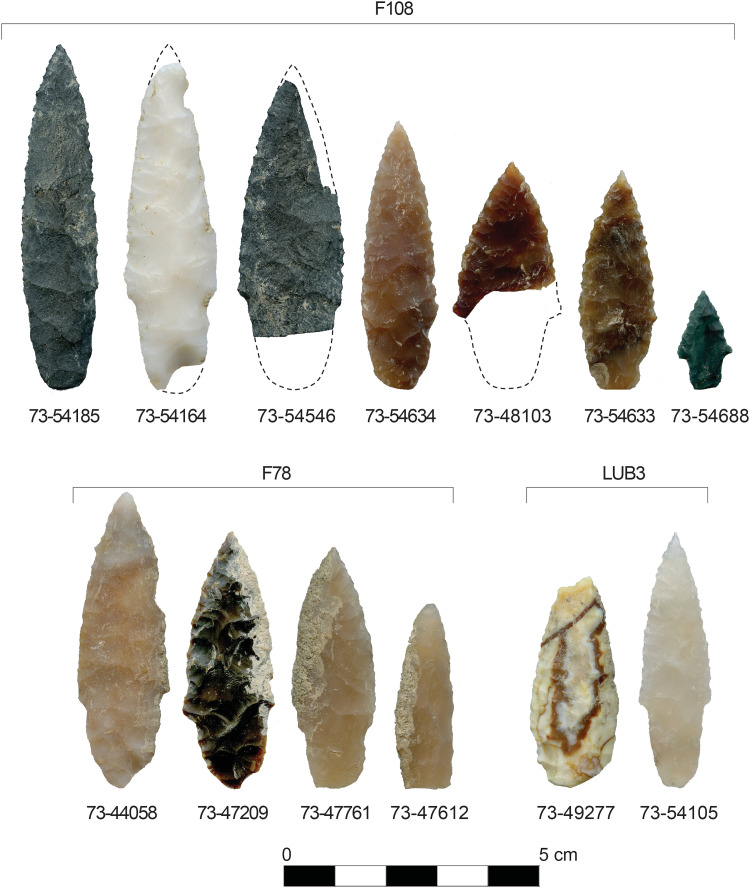
Projectile points from LUB3 sediments, pit F78, and pit F108. Catalog numbers are shown beneath each point (e.g., 73-54185). Dashed lines show estimated extents. A small fragment of a probable stemmed point base found in F78 is not shown here.

Layer LUB3 contained three cylindrical cultural pit features designated as F78, F108, and F151 (Fig. 2 and figs. S4 to S9). Feature 78 (~105 cm in diameter and ~50 cm deep) contained four complete and fragmentary stemmed projectile points (Fig. 4), a small fragment of what appears to be a stemmed point base, a fragment of a biface, a burin spall, a small edge fragment of a core, 250 pieces of debitage, seven pieces of FCR, two pieces of charcoal, and 226 fragments of animal bone (tables S2 and S3). The top of the F78 pit lies below the upper limits of LUB3, as marked by a clear stratigraphic boundary of contrasting pit fill sediments and a large angular basalt cobble, the top of which was buried by the continued deposition of LUB3 loess (fig. S4). Pit F108 (~90 cm in diameter and ~40 cm deep) is positioned immediately north of and at the same elevation as pit F78, as revealed by the presence of artifacts and faunal materials in a carbonate-rich pit fill (figs. S7 and S10). Feature 108 contained seven complete and fragmentary stemmed projectile points (Fig. 4), 53 pieces of debitage, and 21 fragments of animal bone. The top of pit F151 (~75 cm long, 60 cm wide, and ~50 cm deep) also lies below the upper boundary of LUB3 and is capped by a small pile of pebbly sandy loam sediments that contrast in color and texture with the surrounding LUB3 and LUB4 deposits (fig. S9), reminiscent of the cairn found on Area A’s Pit Feature A2 (*11*). Feature 151 contained eight pieces of debitage and 16 animal bone fragments in carbonaceous pit fill.

An animal bone fragment recovered in situ within pit F78 returned two radiocarbon ages of 13,175 ± 48 yr B.P. (15,882 to 15,719 cal yr B.P.) and 13,188 ± 48 yr B.P. (15,914 to 15,740 cal yr B.P.) (Table 1). Two in situ animal bone fragments from pit F108 returned radiocarbon ages of 13,147 ± 55 yr B.P. (15,970 to 15,600 cal yr B.P.) and 13,146 ± 55 yr B.P. (15,975 to 15,590 cal yr B.P.). Three animal bone fragments recovered in situ within pit F151 returned radiocarbon ages of 13,091 ± 48 yr B.P. (15,772 to 15,617 cal yr B.P.), 13,226 ± 52 yr B.P. (15,970 to 15,790 cal yr B.P.), and 13,260 ± 240 B.P. (16,675 to 15,598 cal yr B.P.). These radiocarbon ages show that pits F78, F108, and F151 were probably created at about the same time.

**Table 1.** AMS ages from Area B organized by laboratory number. RN is the reading number. The percent collagen is the yield of extracted collagen as a function of the starting weight of bone samples. C:N is the atomic weight ratio of carbon to nitrogen. %C is the percentage of carbon in the combusted sample. Stable isotope ratios of C and N are expressed in per mil (‰) relative to Vienna Pee Dee belemnite and ambient inhalable reservoir. The calibrations were done using the OxCal 4.3 software (*29*) and the IntCal20 calibration curve (*30*). Missing chronometric data (*) are due to a lack in reporting or measurement on behalf of the laboratories. –, not determined.

RN	Laboratory no.	Material	Northing (m)	Easting (m)	Elevation (masl)	LU	% collagen	C:N	%C	d13C (‰)	d15N (‰)	yr B.P.	± 1 SD	cal yr B.P. (95.4% CI)
44096	D-AMS 045619	Charcoal	60.394	130.680	411.889	Lower LUB16	–	–	*	*	–	1517	20	1415–1345
42302	D-AMS 045621	Charcoal	59.846	132.055	411.306	Lower LUB14, within F99	–	–	*	*	–	9658	37	11,194–11,075
26569	D-AMS 3572	Mussel shell	57.962	132.968	411.403	Lower LUB14	–	–	*	−8	–	9133	38	10,485–10,220
26568	D-AMS 3573	Mussel shell	57.679	132.758	411.379	Lower LUB14	–	–	*	−3.8	–	9106	32	10,375–10,195
26263	D-AMS 3576	Mussel shell	57.823	132.849	411.438	Lower LUB14	–	–	*	−6.2	–	9161	35	10,485–10,235
26264	D-AMS 3581	Mussel shell	57.872	132.908	411.420	Lower LUB14	–	–	*	−11.7	–	9244	36	10,555–10,255
44812	OxA-40353	Bone	58.184	132.724	410.784	Lower LUB5, within F96	6.7	3.2	44.6	−20.08	6.3	9885	31	11,395–11,220
48795	OxA-40375	Bone	59.973	131.831	410.708	LUB3, within F108	1.1	3.3	37.2	−18.87	6	13,146	59	15,975–15,585
48884	OxA-40376	Bone	57.834	133.770	411.179	Lower LUB14, within F111	0.7	3.4	41.9	−19.65	7.6	9566	34	11,095–10,730
45210	OxA-40386	Bone	58.6200	132.482	410.685	Lower LUB5, within F96	6.4	3.3	40.6	−20.11	12.5	9944	39	11,610–11,240
49224	OxA-40387	Bone	58.032	133.760	411.191	Lower LUB14, within F111	1.9	3.3	40.9	−20.14	7.6	9505	38	11,075–10,595
48817	OxA-40389	Bone	60.085	131.349	410.683	LUB3, within F108	1.7	3.3	41.5	−20.51	5	13,147	55	15,970–15,600
44450	OxA-41974	Bone	59.883	130.644	410.708	LUB3, within F151	5.7	3.2	43.6	−20.38	5.6	13,091	48	15,870–15,520
44548	OxA-41975	Bone	59.102	131.553	410.868	LUB3, within F78	2.4	3.3	42.5	−20.33	7	13,188	48	16,000–15,665
44548	OxA-41976	Bone	59.102	131.553	410.868	LUB3, within F78	2.2	3.2	42.5	−20.39	7.1	13,175	48	15,985–15,650
40205	OxA-41977	Bone	59.276	131.383	411.042	LUB4	1.8	3.3	42.4	−20.21	11.2	9572	33	11,100–10,735
44687	OxA-41978	Bone	59.872	130.753	410.634	LUB3, within F151	5.6	3.2	42.8	−20.13	5.3	13,226	52	16,055–15,700
44451	OxA-X-3172-14	Bone	59.836	130.697	410.708	LUB3, within F151	0.9	3.3	41.5	−20.27	4.3	13,260	240	16,675–15,240
44507	OxA-X-3172-15	Bone	59.041	131.283	410.739	Within displaced sediment	2.2	3.4	44.9	−20.43	14.3	9650	100	11,240–10,710
44417	OxA-X-3172-16	Bone	59.308	131.332	410.907	Within displaced sediment	0.7	3.4	42.7	−20.6	11.6	9640	120	11,250–10,595
25816	UCIAMS-144543	Bone	59.507	129.740	410.177	Lower LUB14, within F59	*	*	*	−19.8	7.4	9620	35	11,175–10,775

Excavation of LUB3 sediments outside of these pit features found in situ 10 pieces of debitage, six animal bone fragments, and two stemmed projectile points. One of these is a fragmentary stemmed point (73-49277; Fig. 4) found in situ at an elevation of 410.846 m above sea level (masl), lying ~15 cm below the surface of F78 and therefore dates somewhat earlier. The other stemmed point (73-54105; Fig. 4) was excavated in situ above the top of pit F108 but buried within the upper limits of LUB3 sediments and thus dates sometime after the formation of the pit features but before the erosion of LUB3.

### Pit features originating above LUB3

Four pit features (from oldest to youngest: F96, F99, F59, and F111) were dug downward from other LUs that lie above LUB3 in the vicinity of the composite stratigraphic profile shown in Fig. 3. None of these pits intersected F78, F108, or F151. These younger pit features contained stemmed points in forms different from those seen in LUB3, F78, and F108, an array of stone tools, FCR, debitage, and fragmentary animal bone pieces. Notably, pit F59 contained the partial skeletal remains of a wolverine (*Gulo gulo*), and the pit was capped by a hearth (*12*). Organic samples from these four younger pit features returned five accelerated mass specrometry (AMS) ages ranging between 9944 ± 39 yr B.P. (11,610 to 11,240 cal yr B.P.) and 9505 ± 38 yr B.P. (11,075 to 10,595 cal yr B.P.) (Table 1). The artifacts found in these upper deposits and younger pit features lack the heavy carbonate coatings seen on objects in LUB3 and its inclusive pit features.

### Geochronology

**Fig. 5.**
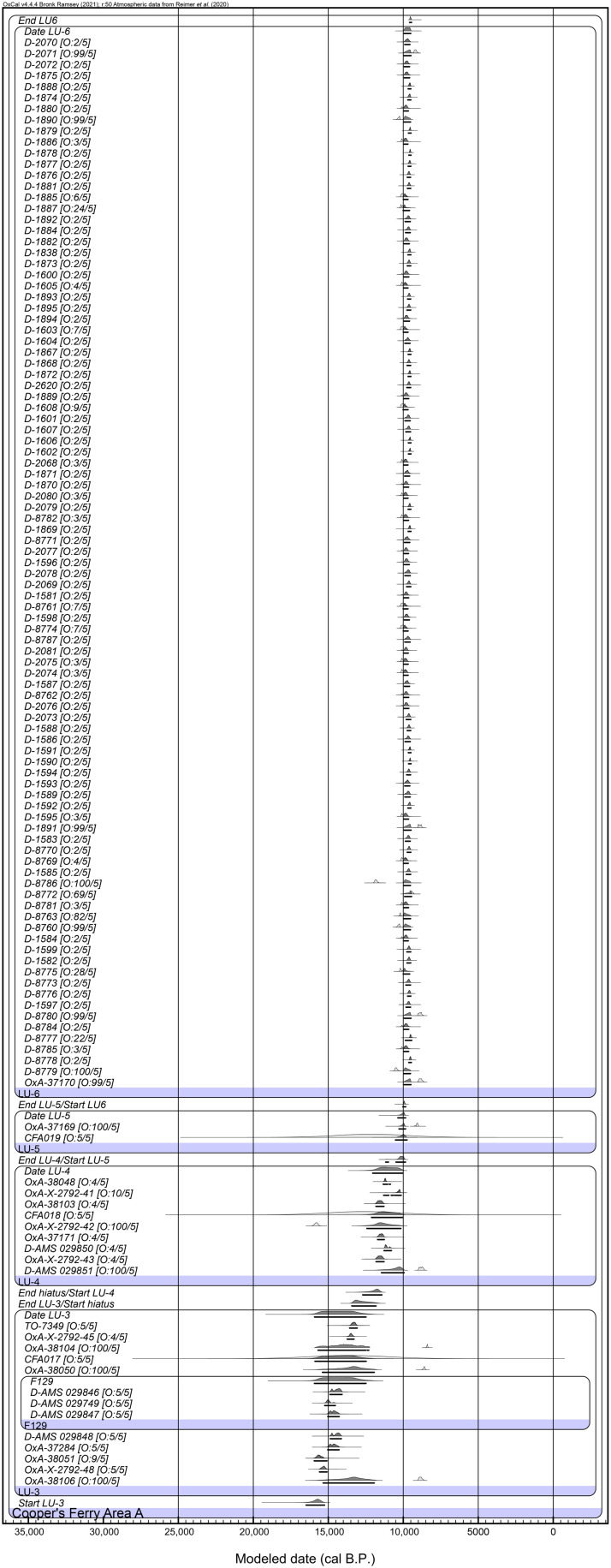
Bayesian model for Cooper’s Ferry Area A. This model includes 94 previously unreported radiocarbon measurements on mussel shells from LU6 and estimates the start and end of LU3 at 16,500 to 15,250 and 13,450 to 11,800 cal yr B.P., respectively, which are comparable to previous results (*1*). Outlier analysis output is noted as “O:posterior probability/prior probability.”

The radiocarbon chronology of Area A was previously modeled (*1*). Here, we report a remodeling of the Area A chronology that includes 94 previously unreported radiocarbon measurements on freshwater mussel shells from LU6 (table S4). No freshwater reservoir offset was applied as seven living mussels from the Salmon River returned modern F^14^C values (table S5). Resolution for this model was set at 50 (years) to ease/speed computing (see OxCal code in the Supplementary Materials). Bayesian modeling places the start and end of LU3 at 16,500 to 15,250 and 13,450 to 11,800 cal yr B.P. (Fig. 5), in agreement with estimates previously obtained for the same stratum (*1*). LU3 is estimated to have a duration of between 2070 and 4195 years (or 2300 to 3500 years at 68.3% CI).

**Fig. 6.**
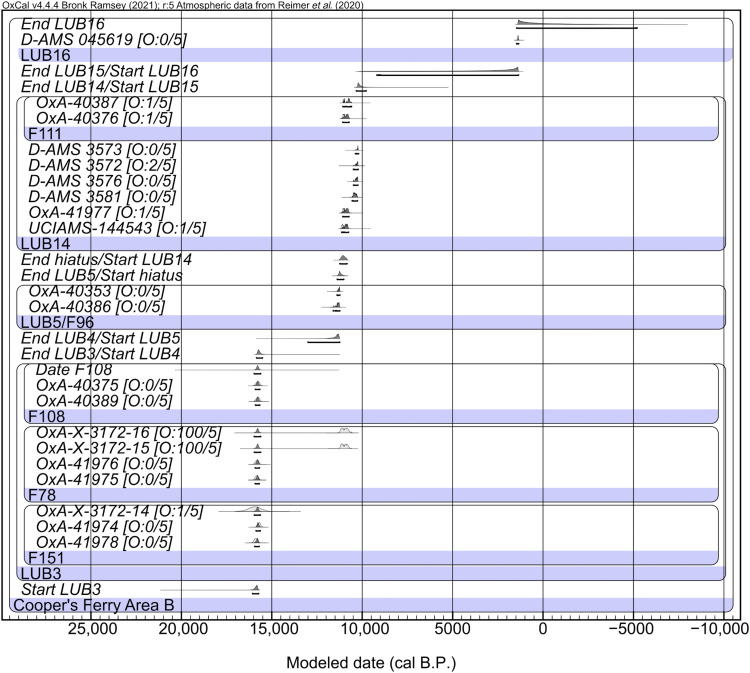
Bayesian model for Cooper’s Ferry Area B. This model estimates the start of LUB3 at 16,045 to 15,725 cal yr B.P. Outlier analysis output is noted as “O:posterior probability/prior probability.”

For Area B, Bayesian modeling of radiocarbon data identifies no major outliers and places the start of LUB3 at 16,045 to 15,725 cal yr B.P. (Fig. 6). This age range is statistically comparable to the estimate for the commencement of LU3 (equivalent) in Area A reported here but is much more tightly constrained (figs. S19 and S20). All features within LUB3 (F151, F78, and F108) are estimated to date to 15,955 to 15,625 cal yr B.P., indicating their likely contemporaneity. LUB3 is estimated to have ended at 15,845 to 15,530 cal yr B.P., considerably earlier than the estimated end of LU3 in Area A, and is attributed to the differential erosion of the LU3/LUB3 surface in Areas A and B. LUB5 likely begins at 12,965 to 11,240 cal yr B.P. following an interval of 4645 to 2755 years that is covered by optically stimulated luminescence (OSL) age 73-15-OSL-Lu2-5 at LUB4 (see the Supplementary Materials for sensitivity testing including OSL ages). Overall, the chronostratigraphic sequence shows good integrity.

The 11 late Pleistocene ^14^C age estimates from LU3 (*1*) in Area A are largely derived from the upper half of the Hammer Creek Loess deposits (Fig. 2) (*2*–*4*), while the seven ages ranging from 13,260 ± 240 to 13,091 ± 48 ^14^C B.P. (16,675 to 15,240 cal yr B.P.) in Area B are derived from the base of the loess unit (Fig. 2) and confirm the modeled start date for the age of the initial cultural occupation previously reported (*1*).

### Characterizing the early Cooper’s Ferry lithic assemblage

Of the 14 projectile point specimens found in LUB3 and pits F78 and F108, 12 were made on cryptocrystalline silicate and 2 were made on fine-grained volcanic rock (Fig. 4). Both kinds of tool stone material are available within ~10 km of the site. Most of the projectile points are relatively small and made on elongate flakes with minimal bifacial reduction. Four larger points (73-44058, 73-54164, 73-54546, and 73-54185; Fig. 4) were more extensively reduced from bifacial preforms. The cross-sectional form of the stemmed points ranges from biconvex to plano-convex, and all show some degree of resharpening on their blade margins. The points typically show collateral flaking patterns, and several retain single beveled blade forms. The smallest stemmed point (73-54688; Fig. 4) is similar in size to a diminutive, stemmed point found at the Gault site in Texas in deposits dated by OSL to ~16 thousand years (ka) beneath a Clovis Paleoindian component (*13*). Many of the F78 and F108 points retain weak shoulders and contracting haft margins—design attributes also seen in a pre-Clovis–aged stemmed point from the Friedkin site dated to ~15.5 ka by OSL (*14*) and among points found in association with mammoth bones and a 14.5-ka tephra layer at Mexico’s Santa Isabel Iztapan site (*14*–*16*). Within the Cooper’s Ferry site, the haft morphometry of stemmed points changes sequentially throughout the late Pleistocene (see the supplementary materials). The early stemmed points from LUB3 and its inclusive pit features bear more subtle shoulders and contracting stem margins that make them similar to but morphometrically different from younger named stemmed point types that are known from the Pacific Northwest region (e.g., Lind Coulee and Windust), indicating a probable evolutionary relationship that requires further exploration. See the Supplementary Materials for additional discussion of projectile point morphology and the results of debitage analysis.

## DISCUSSION

Combined with the previous results from the Area A excavations (*1*), there are now 18 late Pleistocene ^14^C ages that date the cultural materials contained within the Hammer Creek Loess (LU3/LUB3) at Cooper’s Ferry. Together, these support a modeled estimate of 16,045 to 15,725 cal yr B.P. for the initial occupation at the site with intermittent occupation continuing until 13,450 to 11,800 cal yr B.P. when the loess surface was truncated by erosion. Cultural features created during this 2070-to-4195-year period (or 2300 to 3500 years at 68.3% CI) of LU3/LUB3 formation include a hearth, five storage/refuse pits, and what appears to be a food processing surface. Artifacts within the loess consist of 16 complete or fragmentary stemmed points, 30 other stone tools, 482 pieces of debitage, 355 bone fragments, including tooth enamel from an extinct horse, eight pieces of FCR, and a single fragment of freshwater river mussel shell. Fourteen of the stemmed points were deposited before the formation of the Rock Creek Soil and date to between ~16,000 and 15,600 cal yr B.P. Seven radiocarbon dates on animal bone found in direct association within two pit features bearing 12 stemmed projectile points show that humans lived at the Cooper’s Ferry site between ~16,045 and 15,725 cal yr B.P., confirming our earlier findings (*1*). The in situ discovery of a fragmentary stemmed point and other cultural materials from LUB3 loess outside of the pit features indicates that people occupied the site for some time before the dated pit features were created.

This discovery significantly expands both the radiocarbon chronology of human occupation in the Americas and our knowledge of the technological traditions used by its early inhabitants. Progenitors of the First Americans share ancestry with upper Paleolithic peoples of both southern Siberia and eastern Asia and likely became geographically isolated sometime after ~25,000 cal yr B.P. (*17*, *18*) before expanding into the Americas after ~19,500 cal yr B.P. (*19*, *20*). Paleogenetics cannot yet determine where exactly in northeast Asia these ancestors resided, so we must also rely on a close assessment of technological (stone tool) evidence to identify potential regions from which the First Americans may have originated. The nearest and most comparable projectile point form in northeast Asia that predates the ~16,000–cal yr B.P. Cooper’s Ferry occupation is associated with late upper Paleolithic (LUP) bifacial point-bearing sites in Hokkaido (figs. S12 and S13) (*9*, *21*, *22*). This LUP bifacial point tradition is preceded by a blade-point industry dating from ~32,000 to 20,000 cal yr B.P. in Hokkaido and northern Honshu (*21*). These stemmed point assemblages include both the collateral flaking and single beveled projectile point blade forms that occur in the late Pleistocene–aged stemmed points at Cooper’s Ferry (*23*). These bifacial stemmed point technologies occur well before the appearance of different lithic and ceramic technologies associated with incipient Jomon occupations in Hokkaido (~14,700 cal yr B.P.) (*24*–*25*), which may reflect the arrival of different human groups with different cultural adaptations (*25*). Dental and DNA evidence that indicate that Holocene-aged Jomon populations could not be the ancestors of the First Americans (*26*) may thus be correct but is largely irrelevant. We hypothesize that this shared similarity in pre-Jomon stemmed point technology may point to the general location along the northwest Pacific Rim from which some of the earliest peoples in the Americas may have originated between ~22,000 and 16,000 years ago (*27*, *28*).

## MATERIALS AND METHODS

Archaeological excavations in Area B of the Cooper’s Ferry site were conducted from 2012 to 2017. During this time, excavators sought to define cultural features and find items measuring ≥1 cm^2^ in diameter in the ground, so that in situ total station measurements could be made of object locations. Stratigraphic information was recorded in the field during multiple field seasons. Artifact and faunal analyses, including near-infrared analysis of bone samples (see the supplementary materials), were conducted at Oregon State University. Radiocarbon samples reported from Area B were pretreated using standard methodologies at the Oxford Radiocarbon Accelerator Unit, the W.M. Keck Carbon Cycle Accelerator Mass Spectrometer Facility at the University of California, Irvine, and at the DirectAMS laboratory. Accelerator mass spectrometry dating, optically stimulated luminescence dating, and subsequent Bayesian analysis of chronometric results were performed using the protocols described in Supplementary Materials and Methods (see the supplementary materials). All radiocarbon ages were calibrated using the IntCal20 database (*30*).

## Supplementary Materials

### This PDF file includes:

Materials and Methods Supplementary Text Figs. S1 to S28 Tables S1 to S10 References
