# Supplementary material for: Dating of a large tool assemblage at the Cooper’s Ferry site (Idaho, USA) to ~15,785 cal yr B.P. extends the age of stemmed points in the Americas
Source: Sci Adv. 2022 Dec 23;8(51):eade1248. doi: 10.1126/sciadv.ade1248 (PMC9788777; doi:10.1126/sciadv.ade1248)
Supplement: Supplementary file 1 — Materials and Methods Supplementary Text Figs. S1 to S28 Tables S1 to S10 References [file sciadv.ade1248_sm.pdf]

Science Advances
AAAS

Supplementary Materials for

Dating of a large tool assemblage at the Cooper's Ferry site (Idaho, USA) to ~15,785 cal yr B.P. extends the age of stemmed points in the Americas

Loren G. Davis *et al.*

Corresponding author: Loren G. Davis, loren.davis@oregonstate.edu

Sci. Adv. **8**, eade1248 (2022)
DOI: 10.1126/sciadv.ade1248

This PDF file includes:

Materials and Methods
Supplementary Text
Figs. S1 to S28
Tables S1 to S10
References

1-Background and Excavation Methods

The Cooper's Ferry site is located within an alluvial terrace at the confluence of Rock Creek and the lower Salmon River of western Idaho (Fig. 1). The Niimíipuu (the Nez Perce Tribe) refer to this place as an ancient village site named Nipéhe *(38,39)*. Road construction conducted in the 1930s removed more than five meters of sedimentary deposits from the Cooper's Ferry site. These deposits and their archaeological contents are thought to represent the time period between ~8000-2000 yr B.P. (~9450-1950 cal yr B.P.). If not for this road construction the earliest archaeological components at the Cooper's Ferry site would have been buried at a depth of roughly eight meters below the 20th Century landscape surface. Archaeological excavations conducted by B. Robert Butler (*10*) in the 1960s involved a combination of mechanical trenching with a backhoe and manual excavation with hand tools, which produced a trench measuring 2 by 8 meters (Fig. 1). Butler reported the discovery of a stratified sequence of sandy and silty sediments containing more than 1200 pieces of debitage, and 52 stone tools, including six stemmed projectile points. Provenience information for these finds is limited to stratigraphic layers and most items appear to have been found *ex situ* in screened sediments. No radiocarbon ages were obtained. The trench was backfilled at the conclusion of archaeological excavations. This backfill sediment is readily identified by the presence of pre-A.D. 1970s artifacts as well as pre-contact period contained within poorly sorted pebbly to cobbly sands and silts bedded in discontinuous and mixed layers. These historic fill deposits were delineated and excavated separately from undisturbed pre-contact sediments.

Author L.G.D. led excavations at the Cooper's Ferry site in 1997 (*40,41*) and later from 2009-2018 (*42,43*) to clarify the cultural chronology and technological patterns of its Western Stemmed Tradition archaeological components, and to investigate the environmental context of the lower Salmon River canyon during the late Pleistocene to early Holocene period. Excavations in Area B took place during the summers of 2012-2017 (fig. S1). An initial Area B excavation block was established in 2012 (fig. S2) in alignment with the original position of B. Robert Butler's trench. This initial block alignment was adjusted ~50 cm to the north in 2013 to bring the Area B units into alignment with the Area A block grid.

Excavation methods employed careful removal of sediment to expose artifacts, faunal material, cultural features, and other datable samples in their *in situ* positions. During this process excavators also worked to identify, record, and excavate rodent burrows separately from undisturbed sediments. Excavations were typically conducted in 10 cm arbitrary-within-stratigraphic levels and excavators sought to find all archaeological items in place where their positions could be mapped with a laser transit. To avoid confusion, only those items determined not within rodent burrows were mapped in place and are referred to here as *in situ* objects. Excavated sediments were passed through 1/8" wire mesh screen nested atop 1/16" screen mesh.

2-Area B Stratigraphic Descriptions

The stratigraphic record of Area B was recorded from excavation unit walls that were exposed at different times between 2012-2017. Characteristics of these deposits were described using United States Department of Agriculture (USDA) Soil Survey nomenclature (*44,45*) and organized in accordance with concepts and terms presented in the North American Stratigraphic Code (*46*). Textural descriptions follow USDA Soil Conservation Service guidelines (*45*) and

were established from hand-texturing tests. Lithostratigraphic units were defined based on textural composition, inclusive mineralogy, and geometry. The lithostratigraphic and pedostratigraphic units exposed in the a-a' profile shown in Fig. 3 are described in Table S1.

3-Hammer Creek Loess and Rock Creek Soil

The sediments of LU3/LUB3 consist of local yellowish brown sandy and silty aeolian materials termed the Hammer Creek Loess first described at a location 21 km upstream from the Cooper's Ferry site and subsequently at localities further upstream and downstream (*2-4,41*). These sediments are identified as aeolian in origin based on multiple criteria, including the absence of internal stratification, or bedding structures, the angle of repose of deposits (typically greater than 10%), dominance of fine sand- and silt-sized particles, clastic surface morphology, and presence of secondary calcium carbonate content (*47*). Alluvial sediments deposited in the floodplain zone of the lower Salmon River provided a material source for short distance aeolian transport upslope away from the river, resulting in the accumulation of sandy loess on the lower flanks of the canyon (*3,4*). This model of aeolian transport is supported by scanning electron microscopy observations, which revealed abrasion and conchoidal fracturing on the edges and faces of angular to subangular fine to very fine sand grains (*3*). Lancaster (*2*) suggests these aeolian sediments were accumulating in the canyon before and after 13,561±50 yr B.P. (16,560-16,146 cal yr B.P.), while Davis (*4*) and Davis et al. (*3*) indicate the loess began to accumulate before 14,930±1030 yr B.P. (19,400-16,804 cal yr B.P.) and continued until sometime after ~13,090±750 yr B.P. (16,689-14,789 cal yr B.P.).

The Hammer Creek Loess contains a moderately well-developed paleosol termed the Rock Creek Soil that is found at multiple locations up and downstream from the Cooper's Ferry site. The soil is typified by weakly developed cambic horizons of light yellowish brown (10YR 6/4) to brown (10YR 5/3) color with sandy loam to silt loam textures, massive structure, includes common calcium carbonate hypocoatings, fine carbonate filaments, and carbonate pendants formed at the base of pebbles and cobbles. Pedogenic development is constrained to several discontinuous periods. In multiple profiles, paleosols are seen as thin horizons separated by unaltered loess. This is characteristic of the Rock Creek Soil and is interpreted as a cycle involving brief periods of surficial stability followed by renewed aeolian sedimentation and soil burial. At multiple locations within ~40 km upstream from the Cooper's Ferry site, the Rock Creek Soil is characterized by both a Stage II carbonate morphology and an erosional surface that indicates removal of the uppermost soil horizons (*2-4,41*).

At locality SR-26 the Rock Creek Soil is dated by soil humates to before 11,320±80 yr B.P. (13,252-13,159 cal yr B.P.), while at locality SR-27 it is dated by soil humates to at or before 12,220±310 yr BP (14,627-13,798 cal yr BP) and at locality SR-23 to at or before 13,090±750 yr BP (16,689-14,789 cal yr BP) (*3,4*). Further upstream at the Twin Bridges locality, Lancaster (*2*) obtained bulk soil organic matter dates of 12,730±39 yr BP (15,315-15,012 cal yr BP), 12,371±51 yr BP (14,747-14,128 cal yr BP), and 13,561±50 yr BP (16,560-16,146 cal yr BP) for the age of the soil. Together these ages suggest the Rock Creek Soil formed sometime between ~16,450-13,250 cal yr B.P.

The carbonate aspect of the LUB3 deposit is pedogenic based on our observation of common, fine rhizolith features and hypocoatings in pores throughout the matrix, which indicates downward percolation and evaporation of soil water. We also see that carbonates accumulate on the undersides of bone fragments and artifacts, which is also indicative of pedogenic carbonate formation (*48*). If these carbonates were the product of phreatic processes all the artifacts in LUB3 and above would be coated and encased in with carbonate and they are not.

The slight reddish-pinkish colored loess seen in LU3 and LUB3 reflects the development of a cambic subsurface horizon, which includes *in situ* oxidation of parent materials but no significant evidence of clay translocation. The Rock Creek paleosol at Cooper's Ferry is like other loess paleosols described in the Columbia River Plateau region that include paleosol sequences with relatively thin cambic horizons overlying calcic horizons (e.g., Bwb-Bkb; *49,50*). Darker, organic-rich A horizons are uncommon in Plateau loess paleosol sequences, lost either to surficial erosion prior to burial (as in the case of LUB3) or due to oxidation (i.e., decomposition) of the horizon's soil organics over time (*49*).

4-Methods for Identifying Possible Displaced Artifacts in Rodent Burrow Traces (Krotovina)

Burrowing rodents can displace artifacts vertically and horizontally because of their tunneling behavior in a site's sedimentary matrix. In many sites the identification of such displaced artifacts can be difficult because the color and texture of the burrow fills and those of their sedimentary matrix are very similar. Fortunately, in the early sedimentary deposits at Cooper's Ferry krotovina can be readily distinguished from the brown (10YR 5/3) loess of LU3/LUB3 by their much darker brown (10YR 4/3) and grayish brown (10YR 5/2) fill derived from mixing of the overlying depositional units.

Rodent burrow traces at Cooper's Ferry appear as cylindrical tunnels that crosscut through the site's different lithostratigraphic units and can be easily seen as patches and extended polygons on contrasting colored and textured sediments (fig. S14). The site's krotovina typically measure less than 10 cm in diameter, bear slope angles that range from 0-45 degrees, and can appear as individual tunnels or branching networks that extend through one or more lithostratigraphic units. To differentiate between *in situ* archaeological deposits and bioturbated deposits created by burrowing rodents, we developed a set of procedures at the start of the 2009 field season and applied them consistently during all years of excavation (2009-2018). At the start of each excavation level, we identified krotovina by delineating their extents as they were seen in sediments to be excavated and assigning them a unique numerical identifier. During the process of level excavation, we isolated the sediments contained within each krotovina and first excavated non-krotovina sediments, leaving the areas with infilled burrows to stand as pedestals of sediment. Upon reaching the end of the excavation level, the pedestaled krotovina were photographed and their spatial extents recorded with a total station. Finally, the sediments in each krotovina were excavated and screened separately with their archaeological contents bagged and cataloged. By identifying, isolating, recording, and excavating each rodent burrow trace encountered, we were able to separate objects that had been displaced in the site stratigraphy due to bioturbation. We did not use the total station to spatially record items that were found within krotovina to avoid mixing up *ex situ* and *in situ* finds.

This process worked well in helping us distinguish possible displaced artifacts within the loess deposits of LU3/LUB3 but was less useful in identifying possible burrowing within the archaeological pit fills wholly contained within the loess. This was because the dark archaeological deposits within the pits, which allowed us to readily distinguish the pit dimensions and deposits from the surrounding loess in the same way we identified krotovina, were not visibly different from any potential rodent burrows within them. Fortunately, the pedogenic carbonate deposits associated with the formation of Rock Creek soil proved to be a useful tool in recognizing the *in situ* provenience of artifacts within the pit fills.

As with the other artifacts from LUB3, lithic artifacts >1 cm diameter from within the pit fills have extensive artifact coatings resulting from the formation of the Rock Creek soil after the artifacts were deposited. The presence of this carbonate coating confirms these artifacts were not displaced from above by rodent burrowing. On lithic artifacts ≤1 cm diameter, however, carbonate pendants are found differentially on some but not all the smaller lithic artifacts. Carbonates tend to be present on smaller items made of fine grained volcanics but are not consistently present on CCS or obsidian. This may be due to the texture of these toolstone types combined with their small size, with coarser FGV more readily hosting carbonate pendant development than smoother CCS and obsidian. Hence, it is not always possible to confirm the depositional integrity of the very smallest lithic items from within the LUB3 pit fills.

The story is more straightforward for bones from both the pit fills and the LUB3 sediments in that they are grayer in color, more physically weathered, and have thicker carbonate coatings (fig. S3) than items in the younger, overlying LUB4 and F99 deposits. These are readily distinguished from a few small bone fragments from within the pit fills which do not share these characteristics, and which resemble bones from overlying depositional units.

5-Discussion of Archaeological Features

During excavation of LUB3, our team identified deposits that did not match the sedimentological character of known lithostratigraphic units, and which might signal the presence of a different lithostratigraphic or pedostratigraphic unit, an infilled rodent burrow trace, or patterns of sediment and soil alteration resulting from various cultural activities during past occupation of the site. These cultural activities include a range of site formation actions that impart observable characteristics of modification to the site's stratigraphic units, such as digging and refilling pits, heating with fire, and discarding materials that do not match site sediments (e.g., piling rocks, spreading different colored and textured sediments across a surface bearing different geological properties). These processes of site formation are more complex than the simple act of discarding artifacts or faunal materials on an ancient surface. Instead, these processes create definable three-dimensional shapes, which are known as archaeological features. We identified and recorded three archaeological features that were dug into an ancient surface that was present during the original deposition of LUB3 sediments. These three features were interpreted as cultural pits excavated and backfilled in the Pleistocene by the site's human inhabitants. This interpretation is based on the observation that LUB3 pit features are consistent in size, shape, and archaeological content with other pit features that have been described previously from Area A (*1,12,41*) and from Area B (*13,42*). The artifact and faunal content of these previously undiscovered pit

features from LUB3 is consistent with other reported cultural pits and as such, they are generally interpreted to have probably been used to cache equipment and dispose of camp refuse. Intentional caching of points can be inferred from the clustered spatial pattern of stemmed points within F78 and F108 (fig. S4). Broken points may have been included in these caches by the site's occupants with an intention to repair them later, or their internment might have had some other unknown symbolic reason.

Because the pit features were too cylindrical, too large, and too uniform in shape, contain relatively high concentrations of artifacts and faunal materials, and can retain piles of cobbles at their upper limits that suggest cairn construction, we do not think these pit features were caused by large burrowing animals (e.g., coyote, badger) or because of an overturned tree (i.e., "tree throw"). Moreover, their artifact and faunal material content, including concentrations of projectile points, is entirely consistent with other published cultural pit features found previously at the Cooper's Ferry site (*1,12,13,41,42*).

Feature 78 was a cylindrical pit measuring ~105 cm in diameter. Excavation initially identified this feature from its darker colored sediments that contrasted with the surrounding LUB3 deposits and the appearance of a subangular cobble at its western side (figs S4–S8). Excavators mapped 493 artifacts and fragments of faunal materials *in situ*, including seven stone tools throughout the extent of the F78 pit (fig. S10). Four stemmed projectile points were found in the upper half of the pit feature (Fig. 4). A small fragmentary edge of a unidirectional core and a burin flake were found, along with seven pieces of fire cracked rock (FCR). An animal bone fragment excavated *in situ* returned two AMS ages of 13,188±48 yr B.P. (16,000-15,665 cal yr B.P.) and 13,175±48 yr B.P. (15,985-15,650 cal yr B.P.).

Feature 108 was a cylindrical pit measuring ~90 cm in diameter, roughly oriented along a southwest to northeast axis. Excavation initially identified this feature from its circular pattern of darker colored sediments that contrasted with the surrounding LUB3 deposits (fig. S7). Excavators mapped 81 artifacts and fragments of faunal materials *in situ*, including seven stemmed projectile points (Fig. 4 and fig. S10 and S11). Two animal bone fragments excavated *in situ* returned two AMS ages of 13,147±55 yr B.P. (15,855-15,672 cal yr B.P.) and 13,146±59 yr B.P. (15,859-15,666 cal yr B.P.).

Feature 151 was a cylindrical pit measuring ~75 cm long and 60 cm wide, roughly oriented along a northwest to southeast axis. Excavation initially identified this feature from a pile of pebbly loamy sand at its surface, underlain by a circular pattern of darker colored sediments that contrasted with the surrounding LUB3 deposits (fig. S9). Excavators mapped eight pieces of debitage and 16 fragments of animal bone *in situ*, within the pit (fig. S11). Three animal bone fragments returned AMS ages of 13,260±240 yr B.P. (16,175-15,240 cal yr B.P.), 13,226±52 yr B.P. (15,970-15,790 cal yr B.P.), and 13,091±48 yr B.P. (15,772-15,617 cal yr B.P.).

6-Near Infrared Analysis

We used near-infrared (NIR) spectroscopy to non-destructively prescreen bone samples excavated from LUB3, F78, F108, and F151 for collagen preservation following methods described by Sponheimer et al. (*51*). The frequency and amplitude of collagen in the NIR range reflect the type of vibration, concentration, and absorptivity of hydrogen bonds in amino acids

and other hydrogen-rich molecules (C-H, N-H, O-H, S-H) (*52*). Since near-infrared light penetrates approximately 8.5 mm into biological tissues (*53,54*), NIR is an ideal technique to analyze the preservation of collagen in the bone because the outer surface of bone is vulnerable to diagenesis (*55-57*).

A Labspec 4 spectrometer (Malvern Panalytical) equipped with a fiber-optic probe was used to collect the NIR spectra (each spectrum an average of 50 scans) from each bone sample. Each spectrum takes a few seconds to collect, and we prescreened 776 samples over three days at Oregon State Univerisity. We used principal component analysis, partial least squares regression, and visual inspection of spectra from specimens of known collagen yields to characterize the unknown specimens (*51*). These comparative data were then used to make decisions about which bone samples were likely to contain enough collagen for successful AMS radiocarbon dating at the Oxford Radiocarbon Accelerator Unit laboratory (fig. S15).

7-Radiocarbon Dating
Fragmentary bone samples from medium to large animals were submitted for AMS analysis at the Oxford Radiocarbon Accelerator Unit. Methods for sample pretreatment and radiocarbon measurement follow a previous publication on the site (*1*) where these are described in further detail. Calibration and Bayesian modelling were undertaken using the IntCal20 calibration curve (*31*) and the OxCal 4.4 software programme (*58*). Chronometric data, including radiocarbon and luminescence dates (with a measurement date of 2012) for Area A (Table S4) and Area B (Table 1), were included in a multi-phase Bayesian model using stratigraphic information as a prior. The 'General' outlier model was used to identify and downweight outlying dates, with each assigned a prior outlier probability of 5% (*59*). All age estimates here are given at 95.4% credible/confidence intervals (CI) and rounded to 5 years (default resolution).

8-Supplemental OSL Dating
During the early excavation phases of Area B, we collected six quartz optically stimulated luminescence (OSL) samples to serve as rough guides for the approximate ages of the Area B deposits. While the resulting 2σ OSL age ranges of 2000-3000 years are not precise enough to distinguish individual depositional events, they do provide broad support for the more detailed radiocarbon-based chronology.

Sample Collection, Preparation and Measurement
The OSL samples were collected by driving steel tubes (20 cm long, 5 cm diameter) into cleaned vertical sections in Area B. Each tube was sealed in a black plastic and taped shut to avoid light exposure and moisture loss. In the OSL laboratory at Lanzhou University the sediments at each end of the cylinders were removed and used for water content and dose rate measurements. All six samples were measured using a standard single aliquot regenerative-dose (SAR) dating protocol on coarse-grained (90-125 μm) quartz grains (*60*). The purity of the quartz was checked by using an infrared (IR) depletion ratio test (*61*). A preheat plateau test was applied to one sample to determine the SAR measurement conditions. The result shows that the SAR protocol with a preheat temperature of 240 ºC and a second preheat of 200 ºC is suitable for equivalent dose (De) determination (fig. S16). All the samples were measured using a large aliquot size of 4-5 mm to retrieve detected signals. The natural and regeneration dose OSL signal decreased rapidly during the first second of stimulation, indicating that the OSL signal is dominated by a

fast component (fig. S17a). The growth curve was readily fitted using a single saturation exponential (fig. S17b). The average recycling ratio of all aliquots for six samples are 0.98±0.01 (fig. S17c), and the average recuperation ration of natural signal of almost aliquots for six samples are less than 5%, except few aliquots varying between 5-13% (fig. S17d). The Des-overdispersions for all aliquots of the samples are less than 20%. Together these all indicate the quartz OSL dating measurements are reliable. The De values and dose rate of all quartz samples were calculated using LDAC program v1.0 (*62*).

Water Content Estimation
Estimation of the average water content of sediment samples over their depositional history is an important unknown variable in determining OSL age estimates. The problem is particularly fraught at Cooper's Ferry due to its unique depositional history. In the loess regions of western China and elsewhere measured water content usually ranges from ~5-15% and an estimated water content of 10% is often used in calculations of age estimates (*63*). The measured water content of all the Cooper's Ferry samples was also below 10%, but the average water content over the life of the samples was likely much higher. Sometime after the deposition of the loess the nearby Salmon River began to aggrade as the result of a major neotectonic event in the lower Salmon River Canyon (*64*), with the level of the Salmon River eventually exceeding the elevation of the Cooper's Ferry and saturating the loess. For example, as shown in Fig. 1, a distributary channel of Rock Creek, which grades to the Salmon River, overlie virtually the entire intact Area B depositional sequence. Sometime after ~2000 cal BP, the Salmon River cut through the downstream obstruction, the river incised to near its present elevation, groundwater levels dropped, and the loess dried out (*64*). As a result of this complex depositional history, the average water content of the OSL samples is difficult to estimate and we have therefore calculated multiple age estimates using assumed water content levels of 10±5%, 20±5%, 30±5% as shown in Tables S6-S8, respectively.

High Water Content Age Estimates
The calculated age estimates using water content estimates of 30% are generally consistent with the ^{14}C dates from the same stratigraphic units (fig. S18 and Table S9). The exception is the sample from LUB4. LUB4 is a brief alluvial episode that post-dates the erosional event at the top of LUB3 and which likely reworked some of these earlier sediments. Alluvial sediments may produce inaccurate OSL ages, usually aberrant older age estimates, due to poor resetting or partial bleaching of some of the sediments as they are transported and redeposited (*65*).

Summary
Six OSL age estimates from Area B generally support the radiocarbon-based chronostratigraphy for Area B. However, a complex depositional history makes it difficult to adequately assess the average water content of the samples used in the age estimate calculations.

9-Bayesian Modeling
For Area B, sensitivity testing was conducted to test the impact of OSL ages on the chronology described in the main text (henceforth Model "A", which only includes radiocarbon data; Fig. 3). Each alternate model used OSL measurements calculated using different water content estimates (Tables S6-S8). Model "B" included OSL ages with a water content of 10±5% (fig. S20), Model "C" included the OSL dates calculated with a water content of 20±5% (fig. S21), whilst Model

"D" used dates calculated with a 30±5% water content (fig. S22). In fig. S23, we plot the difference in age between the start boundaries of Model A compared with Models B, C and D. The results are statistically identical, suggesting that differences in water content estimates within OSL age calculations do not have a significant impact on our Bayesian chronology for Area B. This is unsurprising since, except for 73-15-OSL-Lu2-2, OSL ages at the base of the sequence (LUB2-3) are generally identified as major outliers in the alternate models and down-weighted accordingly (hence the comparable start estimates for LUB3 as seen in fig. S23). Moreover, the uncertainty in the water content values captures the bulk of variation in the resulting OSL ages at two sigma. Further work is required to better understand the offsets between radiocarbon and OSL ages.

10-Lithic Analysis

Methods for Studying Projectile Points

The 13 projectile points found in LUB3, F78, and F108 were examined under low magnification (10-20x) and measured with digital calipers. A 3D digital model of each point was also created with a David SL-3 structured light system. The resulting digital XYZ point cloud models were processed and their geometric morphometric attributes were characterized using the GLiMR approach (*43,66*).

Visual Observations of Area B Projectile Points

73-54105 (upper portion of LUB3; RN 48558; N 60.204, E 132.094, EL 411.072; Fig. 4). Bifacially flaked projectile point with stemmed haft. Straight to convex haft margins contract below moderate shoulders to a rounded base. Haft margins show edge grinding. Maximum length = 50.1 mm. Maximum width = 14.8 mm. Maximum thickness = 5.9 mm. Manufactured from white to clear CCS material. Biconvex in cross section. Parallel collateral flaking on blade and haft. Minor blade resharpening apparent. Calcium carbonate adheres to one side (i.e., one face of the hafted biface).

73-49277 (middle portion of LUB3; RN 45513; N 59.261, E 132.763, EL 410.846; Fig. 4). Bifacially flaked projectile point with fractured tip and partial stemmed haft. Straight haft margins contract below subtle shoulder and terminate at crosswise fracture. Remnant of haft margin shows edge grinding. Maximum length = 40.3 mm. Maximum width = 17.7 mm. Maximum thickness = 8.1 mm. Manufactured from white, tan, and brown CCS material. Plano-convex in cross section. Parallel collateral flaking on blade. Blade resharpening not apparent. Calcium carbonate adheres to blade margin.

73-44058 (Pit Feature 78; RN 43733; N 58.96, E 131.655, EL 410.83; Fig. 4). Bifacially flaked projectile point with stemmed haft. Convex haft margins contract below moderate to slight shoulders to a narrow rounded base with minor fracturing on one side. Haft margin shows edge grinding. Maximum length = 57.9 mm. Maximum width = 18.3 mm. Maximum thickness = 6.1 mm. Manufactured from white and tan CCS material. Plano-convex in cross section. Flaking performed in roughly collateral pattern with flakes removed at right angles to the long axis of the blade and haft. Partial ventral surface of original macroflake remains on one side. Edge damage apparent on one blade margin. Blade resharpening is minor and limited to distal blade portion. Calcium carbonate adheres to blade margin.

73-47209 (Pit Feature 78; RN 44347; N 59.387, E 131.55, EL 410.953; Fig. 4). Bifacially flaked projectile point with stemmed haft. Convex haft margins contract below moderate to slight shoulders to a narrow rounded base with minor fracturing on one side. Haft margin shows edge grinding. Maximum length = 50.3 mm. Maximum width = 18.2 mm. Maximum thickness = 5.1 mm. Manufactured from brown CCS material. Biconvex in cross section. Flaking performed in roughly collateral pattern with flakes removed at various angles. Blade resharpening is apparent with minor retouch along margins and prominent ears at haft-blade transition. Calcium carbonate adheres to one margin.

73-47612 (Pit Feature 78; RN 44612; N 59.016, E 131.571, EL 410.859; Fig. 4). Bifacially flaked projectile point with fractured stemmed haft. Straight haft margins contract below subtle shoulders and terminate at crosswise fracture. Remnant of haft margin shows edge grinding. Maximum length = 33.5 mm. Maximum width = 13.3 mm. Maximum thickness = 6.1 mm. Manufactured from white to clear CCS material. Plano-convex in cross section. Parallel collateral flaking on convex side and random flaking on planiform side. Blade resharpening is extensive. Calcium carbonate adheres to blade margin.

73-47761 (Pit Feature 78; RN 44684; N 59.105, E 131.372, EL 410.882; Fig. 4). Bifacially flaked projectile point with stemmed haft. Convex haft margins contract below slight shoulders to a rounded base. Haft margin shows edge grinding. Maximum length = 50.0 mm. Maximum width = 17.7 mm. Maximum thickness = 6.9 mm. Manufactured from white to clear CCS material. Biconvex in cross section. Parallel collateral flaking on blade and haft. Some flakes extend beyond the point's longitudinal midline. Blade resharpening apparent with minor flake retouch along margins. Calcium carbonate adheres to one margin.

73-48103 (Pit Feature 108; RN 44799; N 59.876, E 131.827, EL 410.802; Fig. 4). Bifacially flaked projectile point blade fragment. Small remnant of ear at haft-blade transition provides evidence that this point probably appeared like specimen 73-47209, which has a stemmed haft. Maximum length = 29.6 mm. Maximum width = 20.5 mm. Maximum thickness = 5.4 mm. Manufactured from brown CCS material. Plano-convex in cross section. Parallel pressure flakes extend past midline on one side and remain on edge margins on other side where ventral surface of original macroflake remains. Blade resharpening is evident on all margins and produced an ear on the remaining haft-blade transition portion. Calcium carbonate adheres to one side.

73-54164 (Pit Feature 108; RN 48600; N 60.148, E 131.809, EL 410.969; Fig. 4). Bifacially flaked projectile point with stemmed haft. Convex haft margins contract below moderate shoulders and terminate at a fragmentary rounded base. Haft margin shows edge grinding. Fractures present on distal blade tip and along blade margins. Retouch along blade margins from opposing faces of blade produced single beveled blade form. Maximum length = 63.3 mm. Maximum width = 19.1 mm. Maximum thickness = 6.4 mm. Manufactured from white CCS material. Biconvex in cross section. Parallel collateral flaking on blade and haft. Flake removal at base and lateral burination of tip suggest bipolar compression due to kinetic impact. Calcium carbonate adheres to one side.

73-54185 (Pit Feature 108; RN 48616; N 60.111, E 132.125, EL 410.952; Fig. 4). Bifacially flaked lanceolate projectile point. Haft element discernable from inflection where edge margins

contract to flat basal facet. Haft margin shows edge grinding. Retouch along blade margins produced single beveled blade form. Maximum length = 67.6 mm. Maximum width = 15.3 mm. Maximum thickness = 11.4 mm. Manufactured from blueish-gray fine grained volcanic material. Biconvex in cross section. Parallel collateral flaking to over-midline flaking on blade and haft. Calcium carbonate adheres to one side.

73-54546 (Pit Feature 108; RN 48685; N 60.065, E 132.068, EL 410.90; Fig. 4). Bifacially flaked projectile point fragment with fractured haft and blade margin. Remnant of haft margins contract to lateral break and bear edge grinding. Burination fracture removed tip and portion of one blade margin. Retouch along blade margins present and produced small ear on one side at haft-blade transition. Maximum length = 50.0 mm. Maximum width = 26.8 mm. Maximum thickness = 6.9 mm. Manufactured from bluish-gray fine grained volcanic material. Biconvex in cross section. Roughly parallel flaking on blade and haft cross over midline from both sides. Burinated blade suggests kinetic impact. Calcium carbonate adheres to one side.

73-54633 (Pit Feature 108; RN 48725; N 60.120, E 131.607, EL 410.875; Fig. 4). Bifacially flaked projectile point with stemmed haft. Convex haft margins contract below subtle shoulders and terminate at flat base with minor fracturing on one side. Maximum length = 44.8 mm. Maximum width = 16.6 mm. Maximum thickness = 6.1 mm. Manufactured from brown CCS material. Biconvex in cross section. Near-parallel collateral flaking on blade and haft. Blade resharpening is minor but has produced a small ear at the haft-blade transition on one side. Calcium carbonate adheres to one side.

73-54634 (Pit Feature 108; RN 48730; N 60.099, E 131.707, EL 410.859; Fig. 4). Bifacially flaked projectile point with stemmed haft. Convex, contracting haft margin below subtle shoulder present on one side while other bears straight contracting haft margin below a shoulderless haft-blade inflection. Maximum length = 52.7 mm. Maximum width = 15.5 mm. Maximum thickness = 6.1 mm. Manufactured from light brown CCS material. Plano-convex in cross section. Parallel collateral flaking on blade and haft faces. Blade resharpening evident in removal of abundant small retouch flakes along both margins. Calcium carbonate adheres to one side.

73-54688 (Pit Feature 108; RN 48771; N 59.991, E 131.943, EL 410.798; Fig. 4). Bifacially flaked projectile point with stemmed haft. Straight haft margins contract below moderate shoulders and terminate at flat basal facet. Haft margins show edge grinding. Maximum length = 19.5 mm. Maximum width = 15.8 mm. Maximum thickness = 2.7 mm. Manufactured from green CCS material. Plano-convex in cross section. Minor edge retouch preserves original dorsal and ventral surfaces. Blade resharpening produced an ear on one side at haft-blade transition. Calcium carbonate adheres to one side.

Preliminary views on 3D Geometric Morphometry of Early Projectile Points at Cooper's Ferry
Preliminary evaluation of the 3D geometric morphometric attributes for these points reveals some important patterns. Haft reentrant (i.e., the negative space that lies between the convex hull and the haft margin; fig. S24) size and shape is relatively small in the older F108, F78, LUB3, and Area A points, and increases in size over time in points from Pit Feature A2 (PFA2) and Pit Feature P1 (PFP1) (figs. S25-S27). This pattern shows that there are clear differences in the haft

design of stemmed points found in the lower and upper portion of the LU3/LUB3 deposit and signals what may be a progressive evolutionary sequence in the development of hafting design at Cooper's Ferry. More work is needed to fully assess morphometric differences among stemmed points at the Cooper's Ferry site.

Other Stone Tools
One small fragment of a biface made on CCS material was found in F78. A burin spall made on CCS material showing the creation of a burin tool was found in F78. A small fragment of a unidirectional flake core made on CCS material was found in F78.

Debitage Analysis
We employed a lithic attribute analysis system that is grounded in experimental studies of lithic reduction sequences and their corresponding products to provide a preliminary analysis of all provenienced debitage (*67-70*). Visual observations of debitage were made with a Leica zoom binocular microscope at magnification ranging from 3.5X to 22.5X and lithic attributes were recorded.

Debitage from LUB3 (n = 10) is mostly made from cryptocrystalline silicate (CCS) rock and analysis indicates early and late stage biface reduction via percussion. One flake shows early stage core reduction. Four pieces of fine grained volcanic (FGV) debitage show percussion reduction of early stage bifaces.

Feature 78 debitage (n = 250) shows primarily early and late biface reduction of CCS material through percussion and pressure methods, followed by early and late core reduction of CCS. Less common are FGV flakes showing early biface reduction and early core production, mainly through percussive reduction. One small obsidian pressure flake was found *in situ* within F78. X-ray fluorescence analysis produced indeterminate results due to the object's small size.

Feature 108 debitage (n = 53) is dominated by CCS materials made during early and late stage biface reduction. One FGV flake shows early biface reduction, and three others show indeterminant percussion reduction.

Feature 151 contained eight *in situ* pieces of debitage. Analysis of these CCS and FGV pieces show a range of percussion and pressure techniques applied to make bifaces at early and late stages of production.

Artifact Totals from LUB3, F78, F108, F151, and LU3
Frequencies of artifacts found in LUB3 and its early pit features, and in LU3 (*1*), are reported in Table S2. Two Levallois-like cores, previously reported by Davis and Willis (*71*), were excavated *in situ* from LU3 in Area A and are included here in the artifact totals.

11. Examination of Faunal Materials
Authors L.G.D. and C.W.E. examined the bone fragments found *in situ* and from screened sediments within LUB3 and features 78, 108, and 151 to identify any potential human skeletal remains and to evaluate whether specimens could be assigned to taxonomic groups. No definitive human remains were identified among the bone fragments recovered from Area B. L.G.D.

reviewed all bone submitted for radiocarbon analyses and did not observe any specimens bearing cortical thicknesses or morphological and anatomical attributes consistent with human bone. The bone specimens from LUB3 and its inclusive cultural features were very fragmentary and generally lack clear anatomical features and could be only identified to the class *mammalia* based on their size and cortical thickness. The excavated bone fragments most likely represent different small, medium, and large bodied mammals. Because of this situation, we hope to use instrumental analyses (e.g., ZooMS) to provide faunal identifications of the bone fragments. Knowing more about the animal taxa present in these pit features could help clarify paleoecological conditions, economic patterns of site occupants, and help to interpret the function of the pit feature. Frequencies of faunal materials found in LUB3 and its early pit features, and in LU3 (*1*), are reported in Table S3.

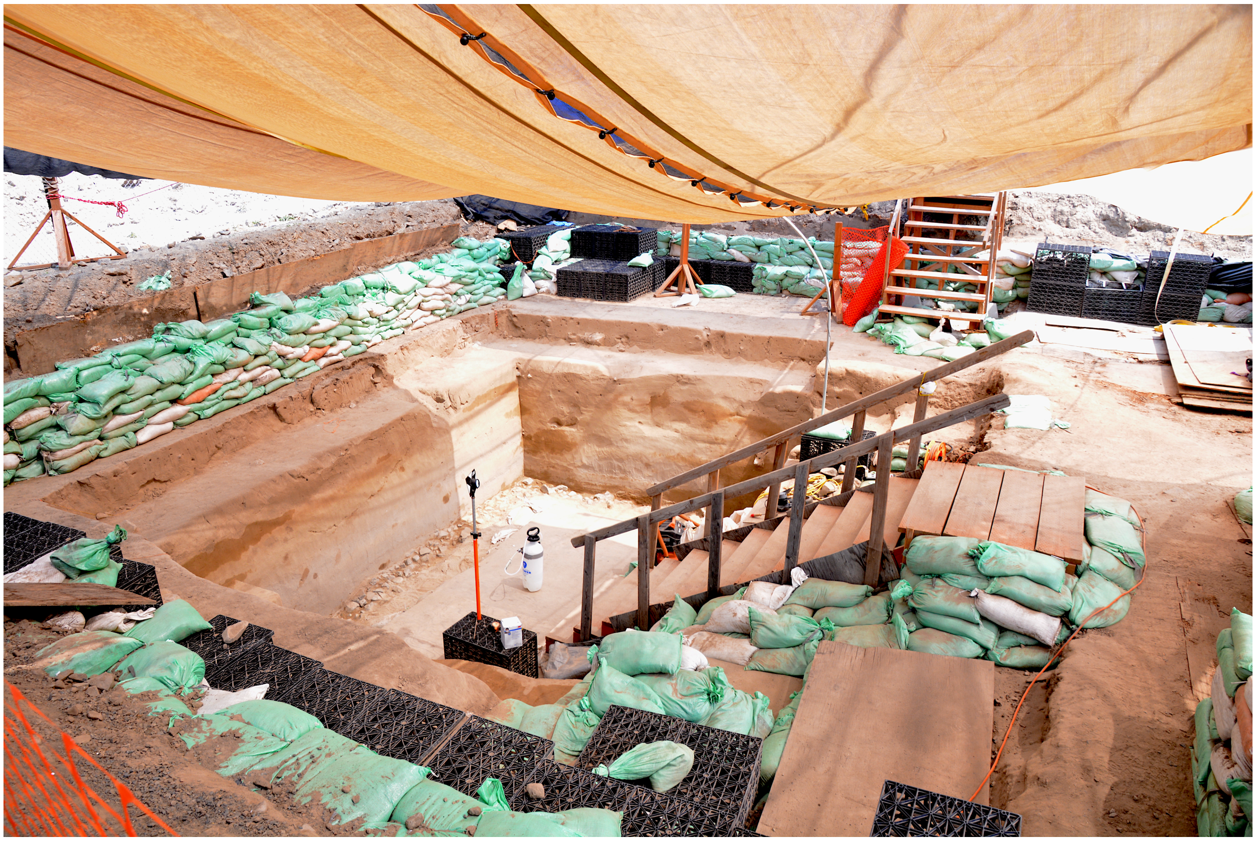

Fig. S1. Overview of Area B excavations at the Cooper’s Ferry site during the summer of 2017. View to the east.

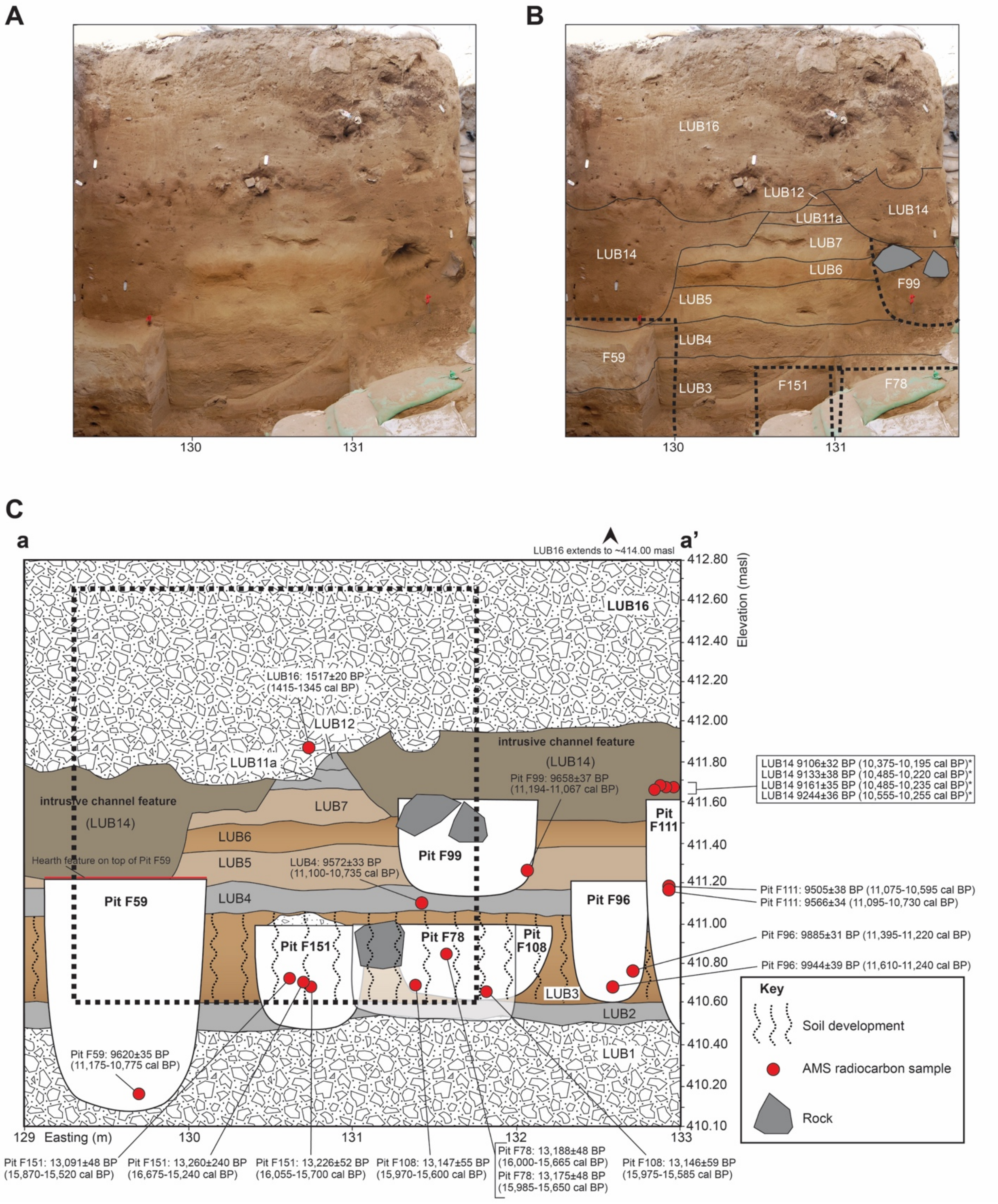

Fig. S2. Comparison of stratigraphic profiles along the a-a’ transect, Area B. Cleaned stratigraphic profile exposed along the a-a’ transect in 2012 (A). Stratigraphic boundary lines overlain on cleaned profile (B). Composite stratigraphic drawing showing stratigraphic units, pit features, and radiocarbon ages (C). Dashed black box in (C) shows orientation of stratigraphic profiles shown in (A) and (B) relative to the composite stratigraphy drawing.

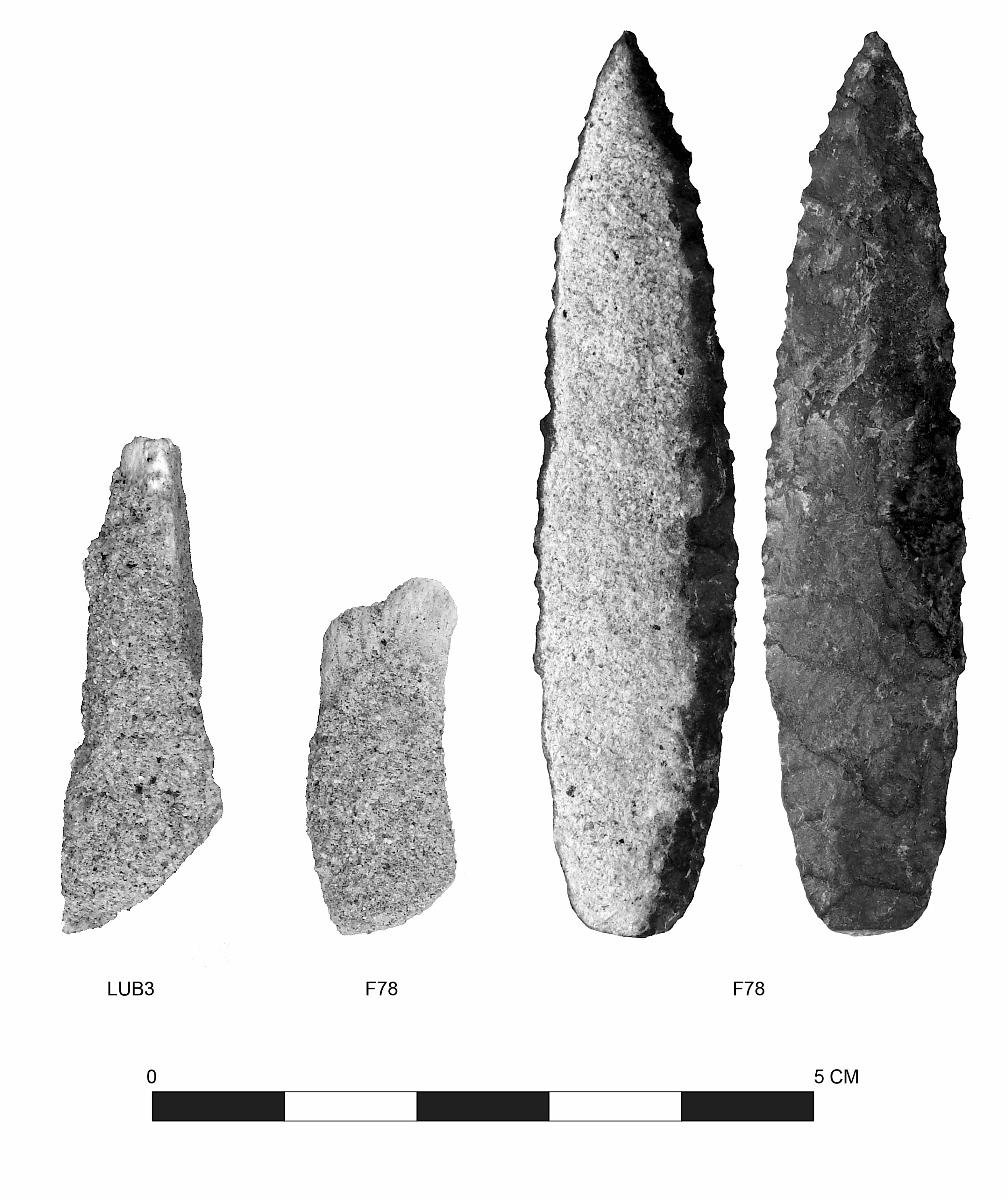

Fig. S3. Examples of carbonate coatings. Carbonates are pedogenic and occur on the undersides of objects, caused by formation of the Rock Creek Soil seen on bone and artifacts from LUB3 and its cultural pit features. Both sides of point 73-54185 from F78 are shown at right.

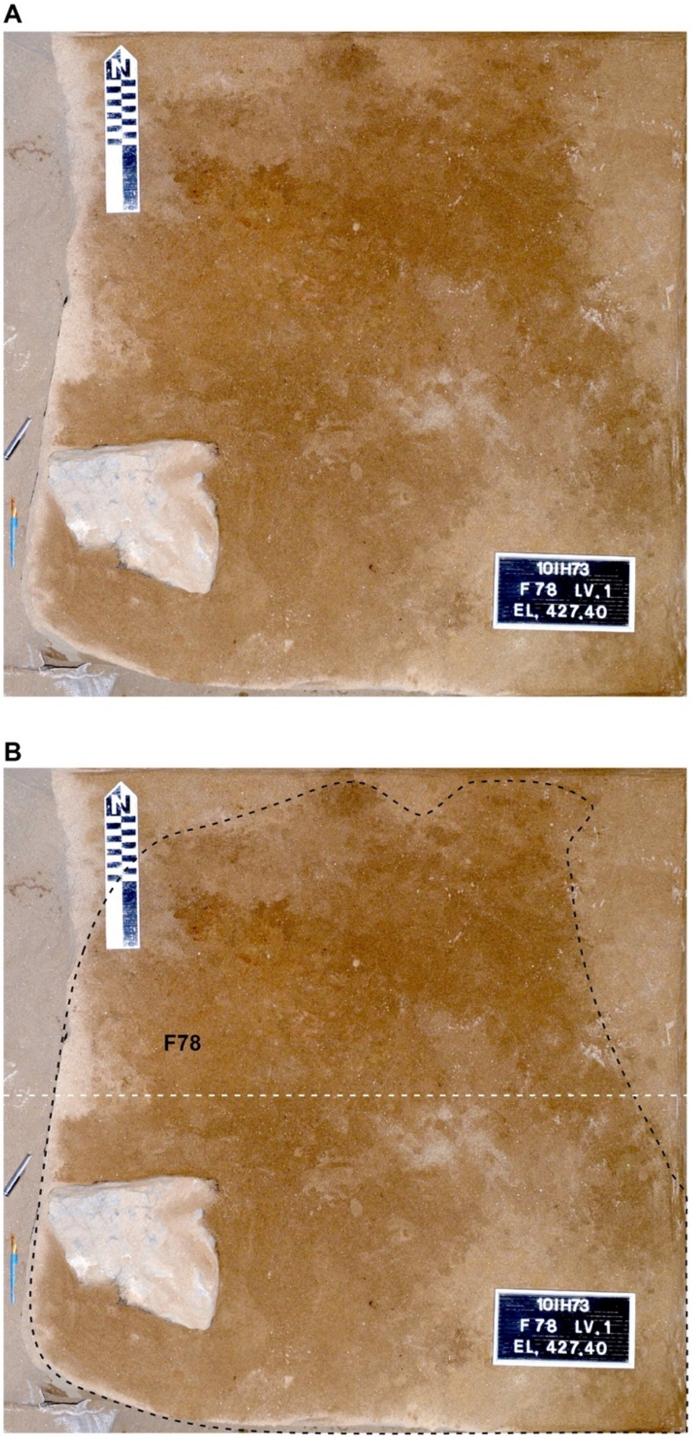

Fig. S4. Plan view of F78 showing contrasting pit fill matrix and cairn stone as seen in 2015 (A). Elevation (EL) of 427.40 recalculates to 410.98 masl, following adjusted site datum used in Davis et al. (*1*). Dashed black line shows boundaries of F78 when first exposed during excavation (B). The shape of F78 becomes more circular in deeper excavation levels. Dashed white line shows position of wall profile shown in fig. S5 and S6.

A

B

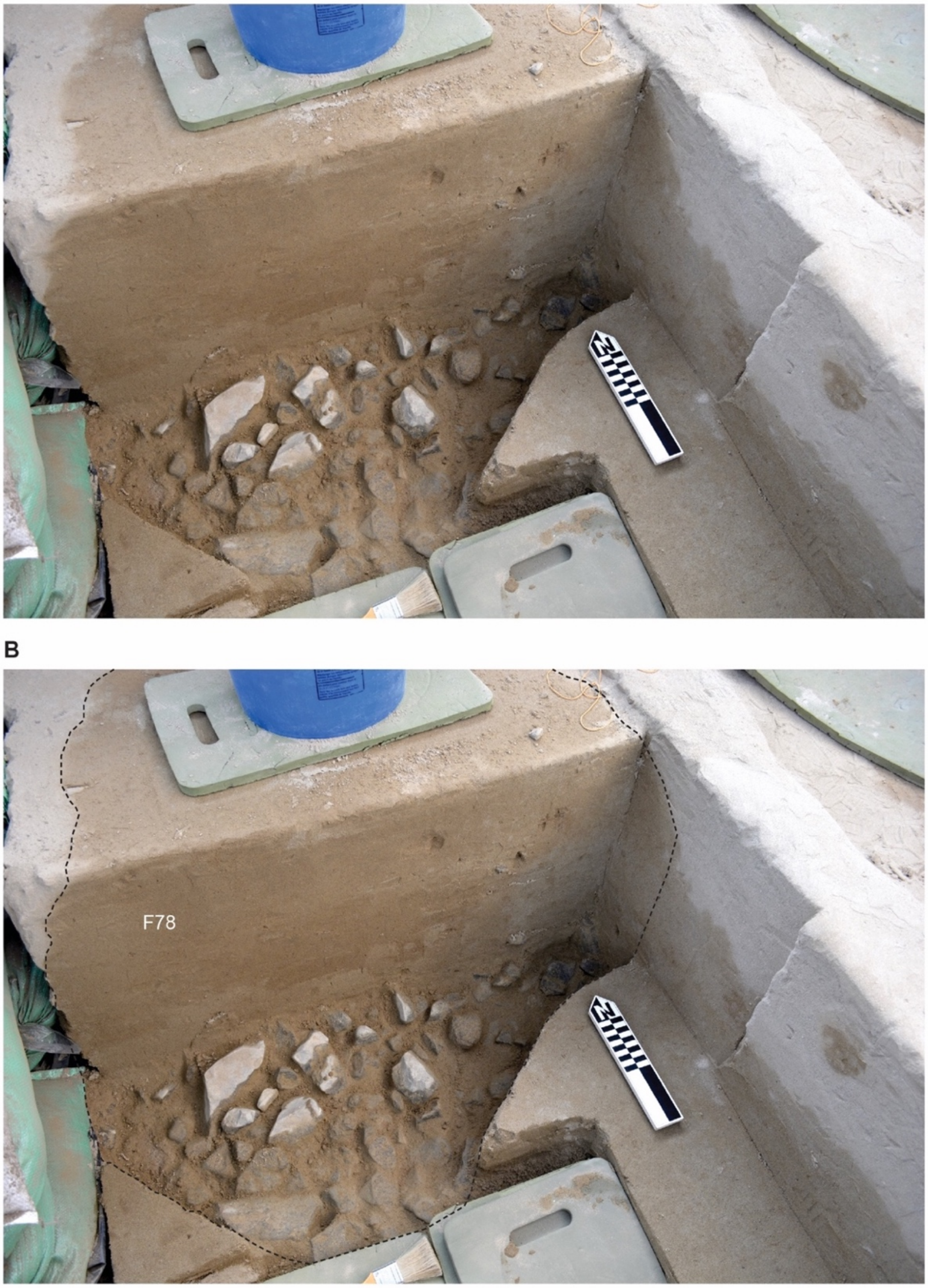

Fig. S5. Oblique photo showing F78 sediment infill and base of pit on LUB1 gravels (A). Extents of pit feature 78 boundaries drawn in black dashed line on stratigraphic profile (B). The plan shape of F78 became more circular a few centimeters below its upper surface.

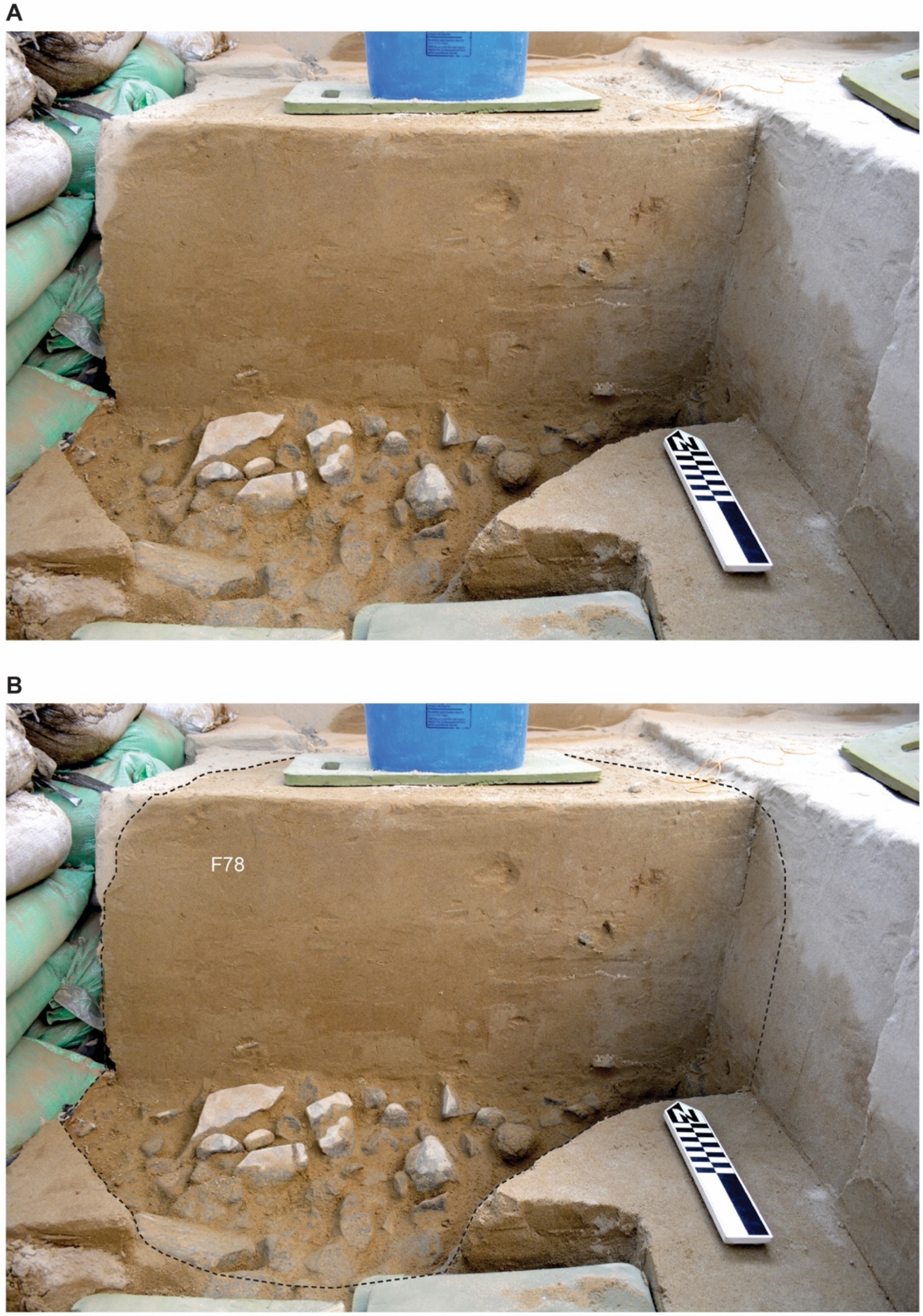

Fig. S6. Profile of the F78 pit fill, view to north (A). The extent of pit feature 78 is shown as a black dashed line (B).

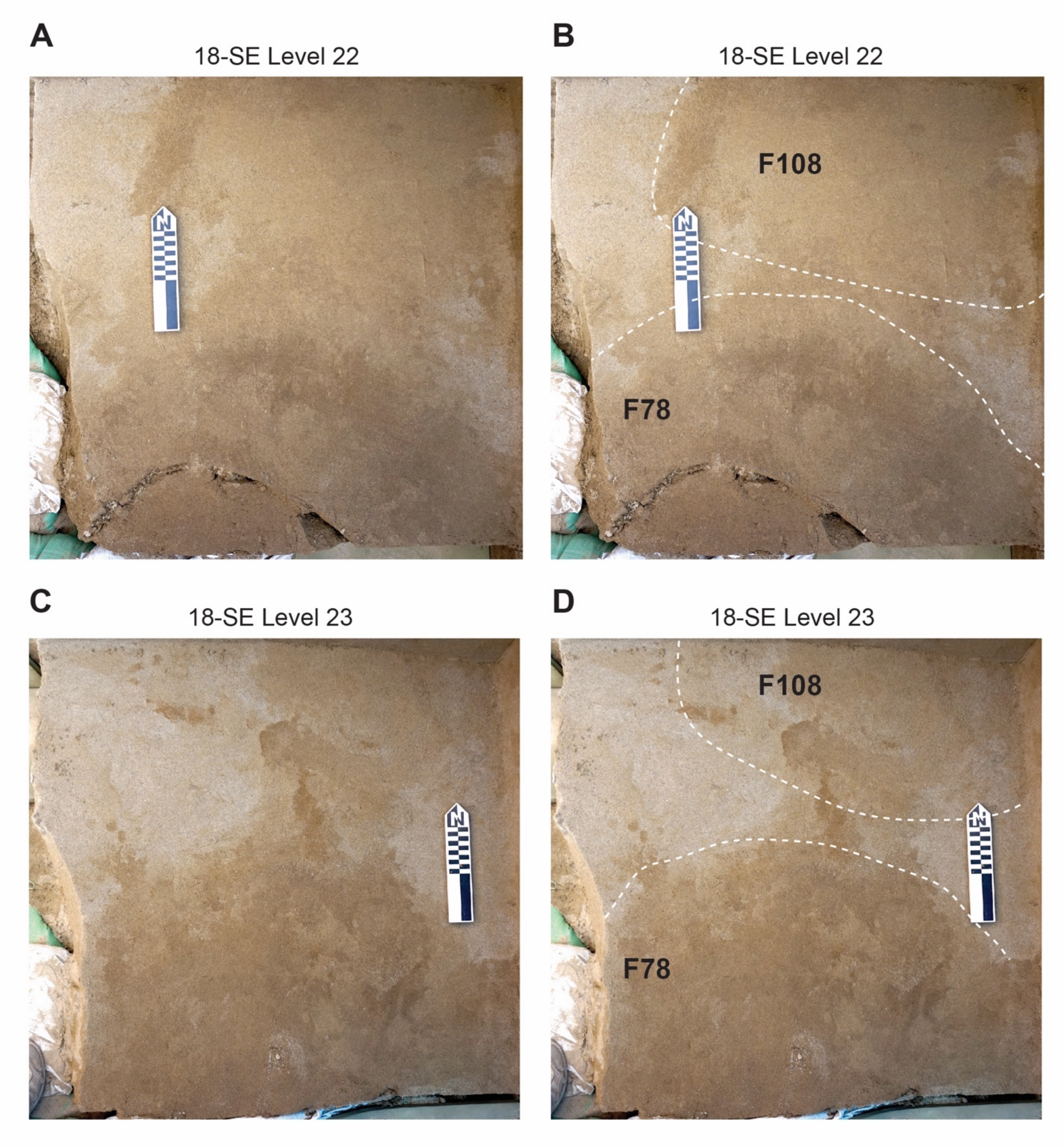

Fig. S7. Photograph montage of pit features 78 and 108 as seen in levels 22 and 23. A semi-circular wedge of displaced sediment is seen in the lower portion of F78 in images A and B.

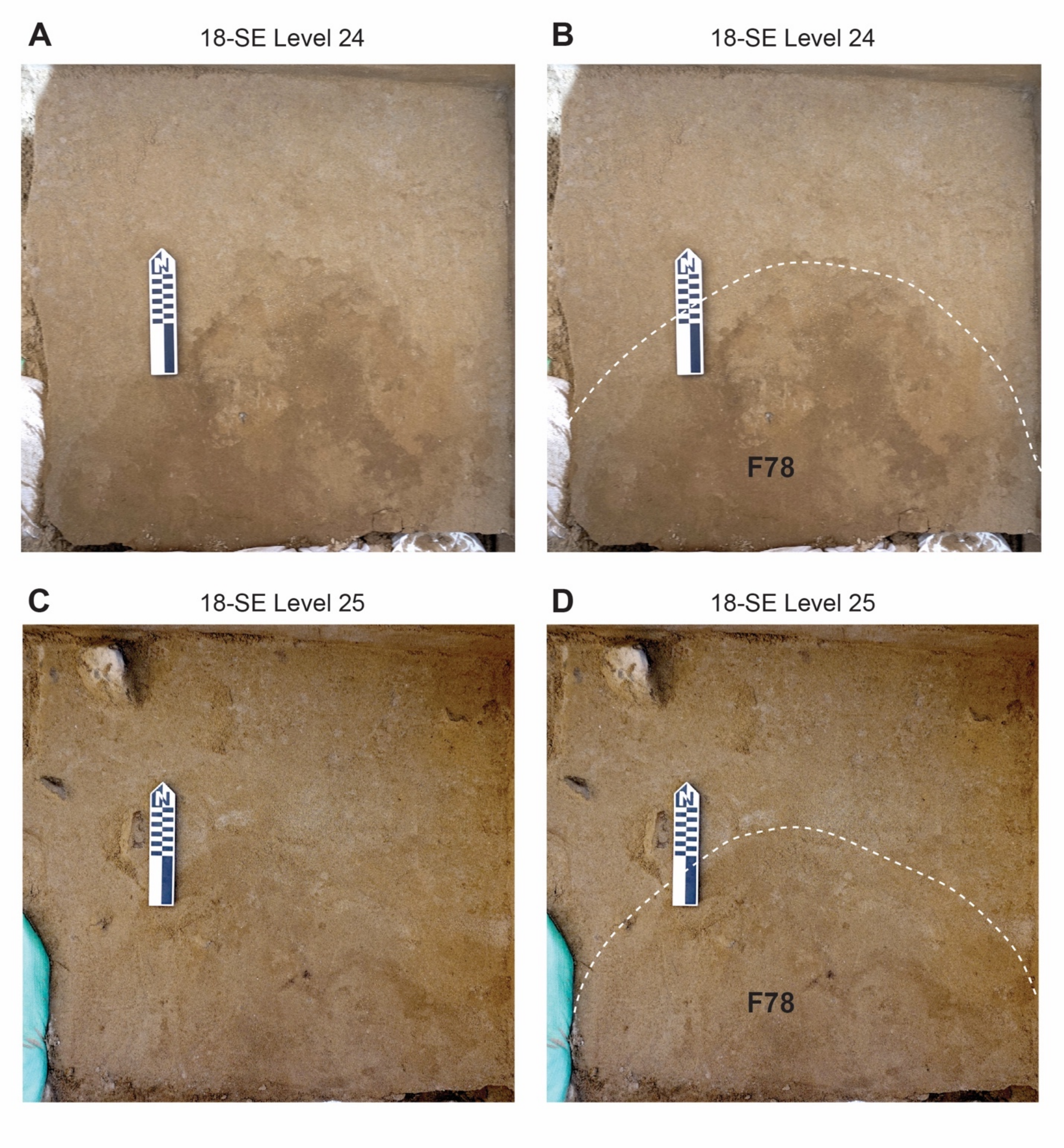

Fig. S8. Photograph montage of pit feature 78 as seen in the completion photos for levels 24 and 25. Feature 78 is more readily seen in these levels. Artifact and pit feature boundaries for F108 began to disappear during the excavation of level 24.

A

B

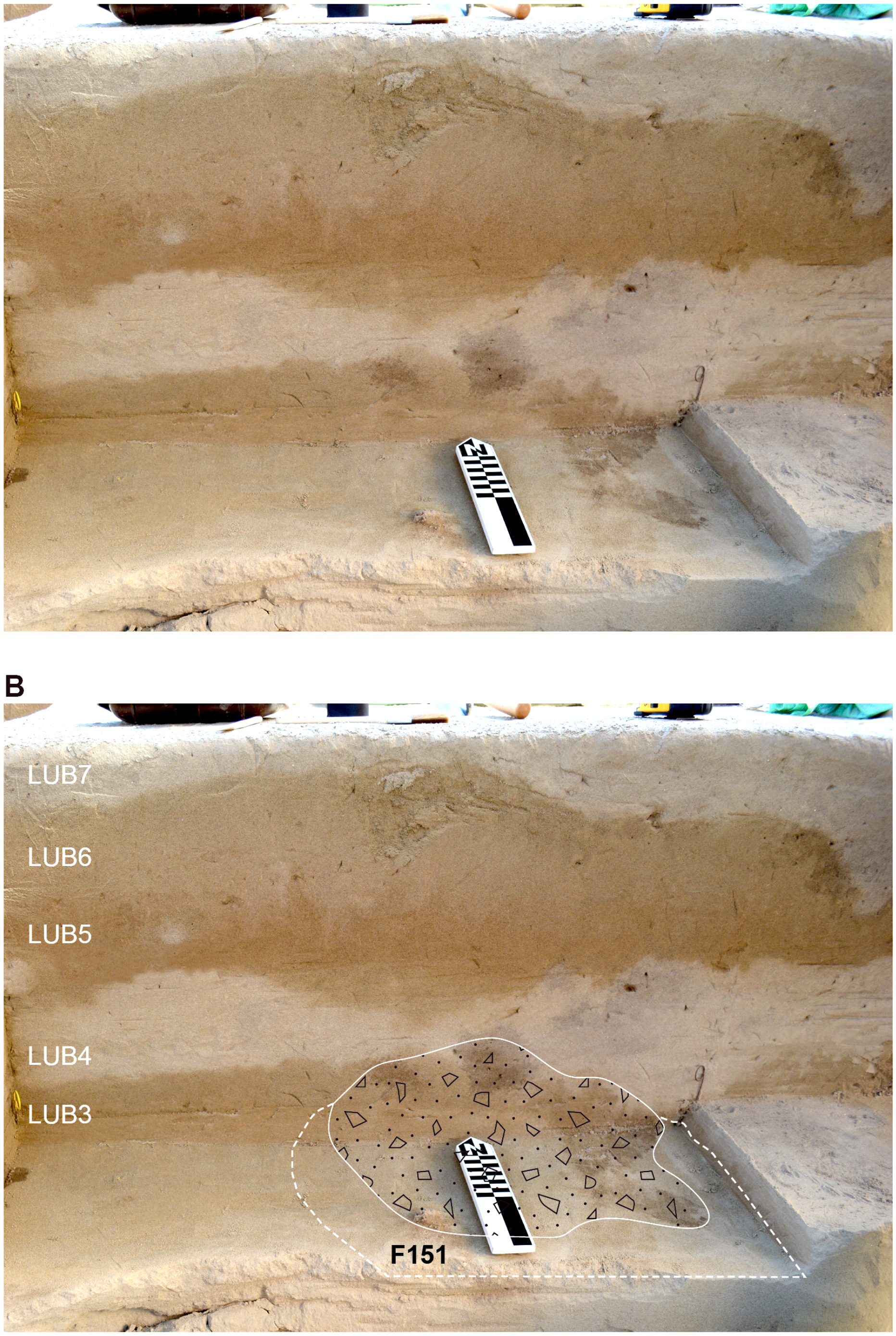

Fig. S9. Stratigraphic profile showing the location of a pile of pebbly sandy loam sediments that contrasted in color and texture with the surrounding LUB3 and LUB4 deposits (A). The extents of this pebbly deposit that were encountered at the top of F151 in excavation Unit 18-SW are drawn with a solid white line and gravelly fill pattern (B). The extents of F151 were revealed during excavation and shown within excavation Unit 18-SW with a white dashed line.

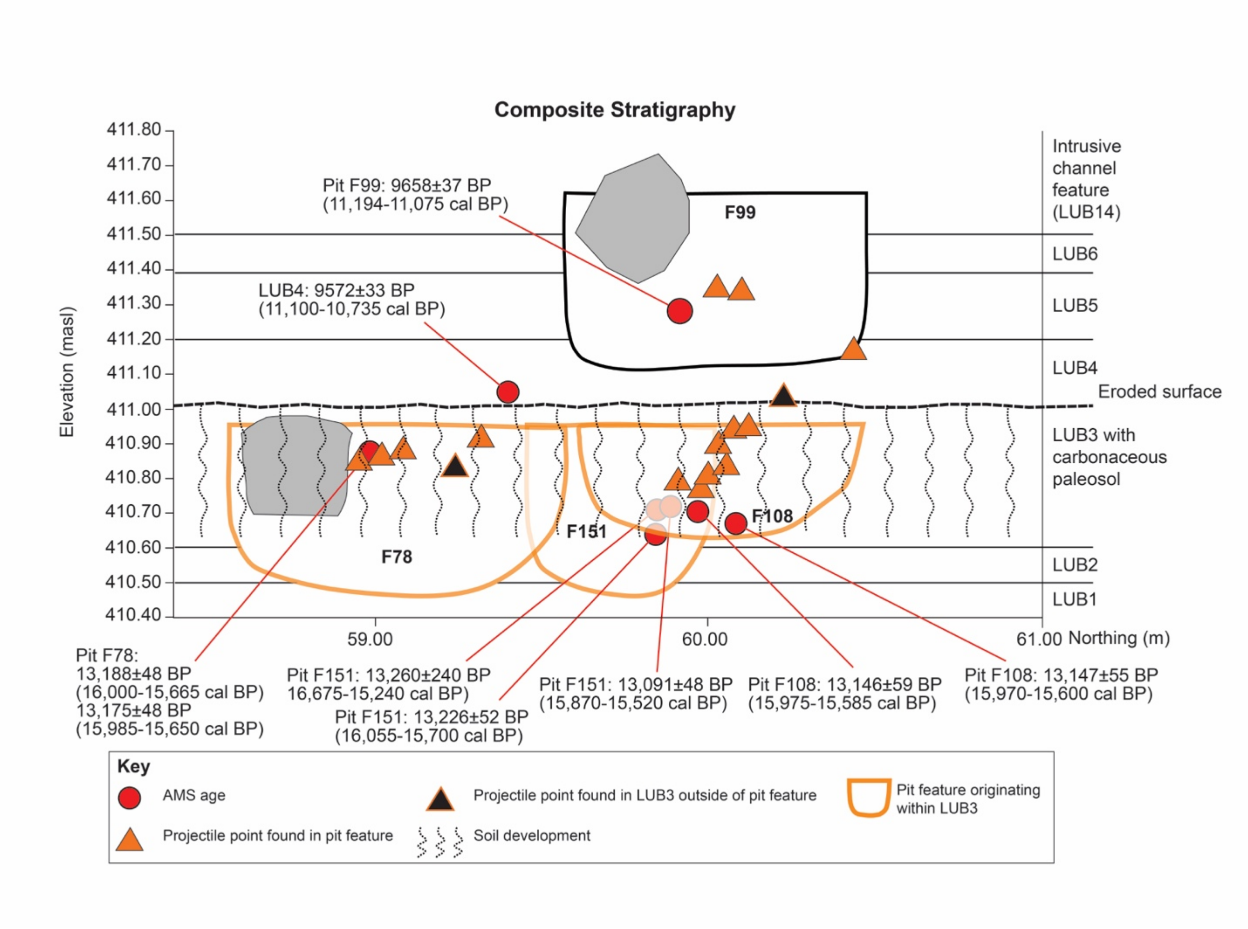

Fig. S10. Composite stratigraphy showing the northing backplot of projectile points and radiocarbon dated samples associated with the F78, F108, and F99 pit features. Pit features originating within LUB3 are shown with orange outlines.

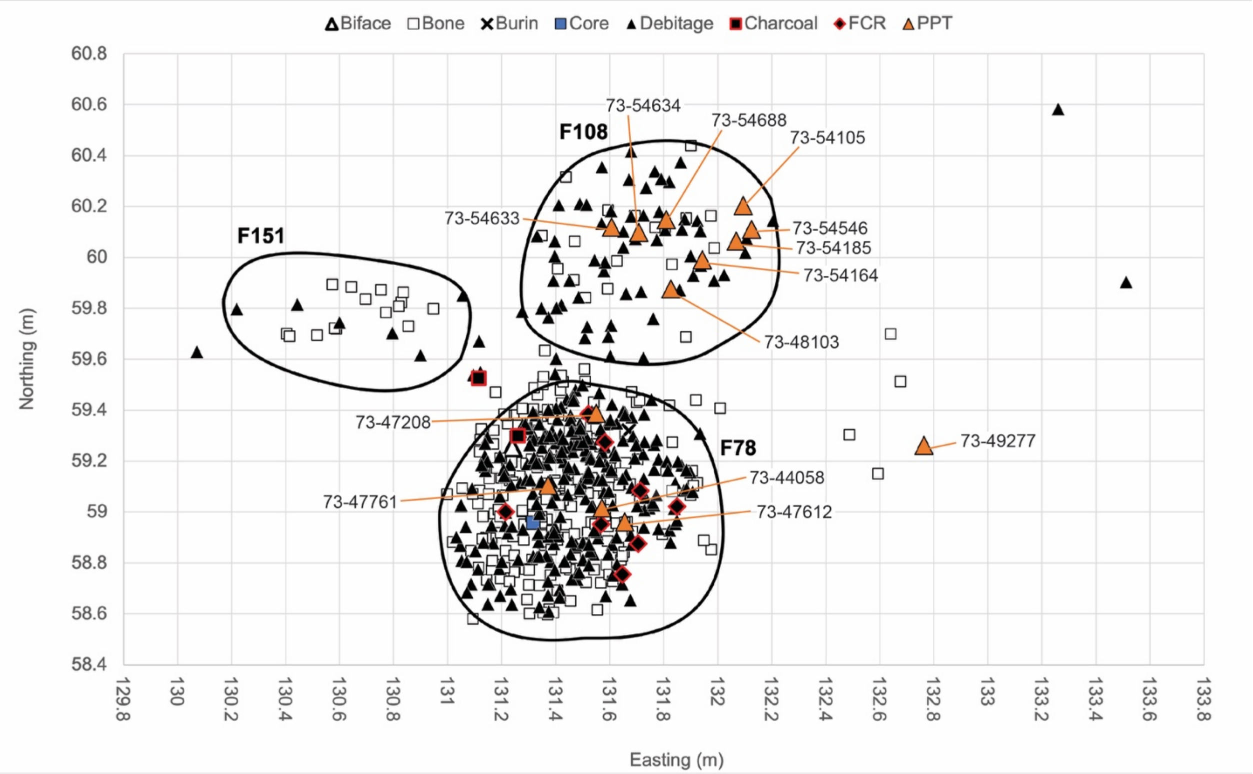

Fig. S11. Plan view of *in situ* artifact and faunal material distribution in LUB3, F78, F108, and 151. Projectile points are designated by their associated catalog numbers (e.g., 73-54546). Projectile points 73-54105 was found above F108 in the upper portion of LUB3 and point 73-49277 was found in LUB3 sediments but deeper than the tops of the pit features. Black lines around plotted items show approximate shape of pits, as revealed during excavation.

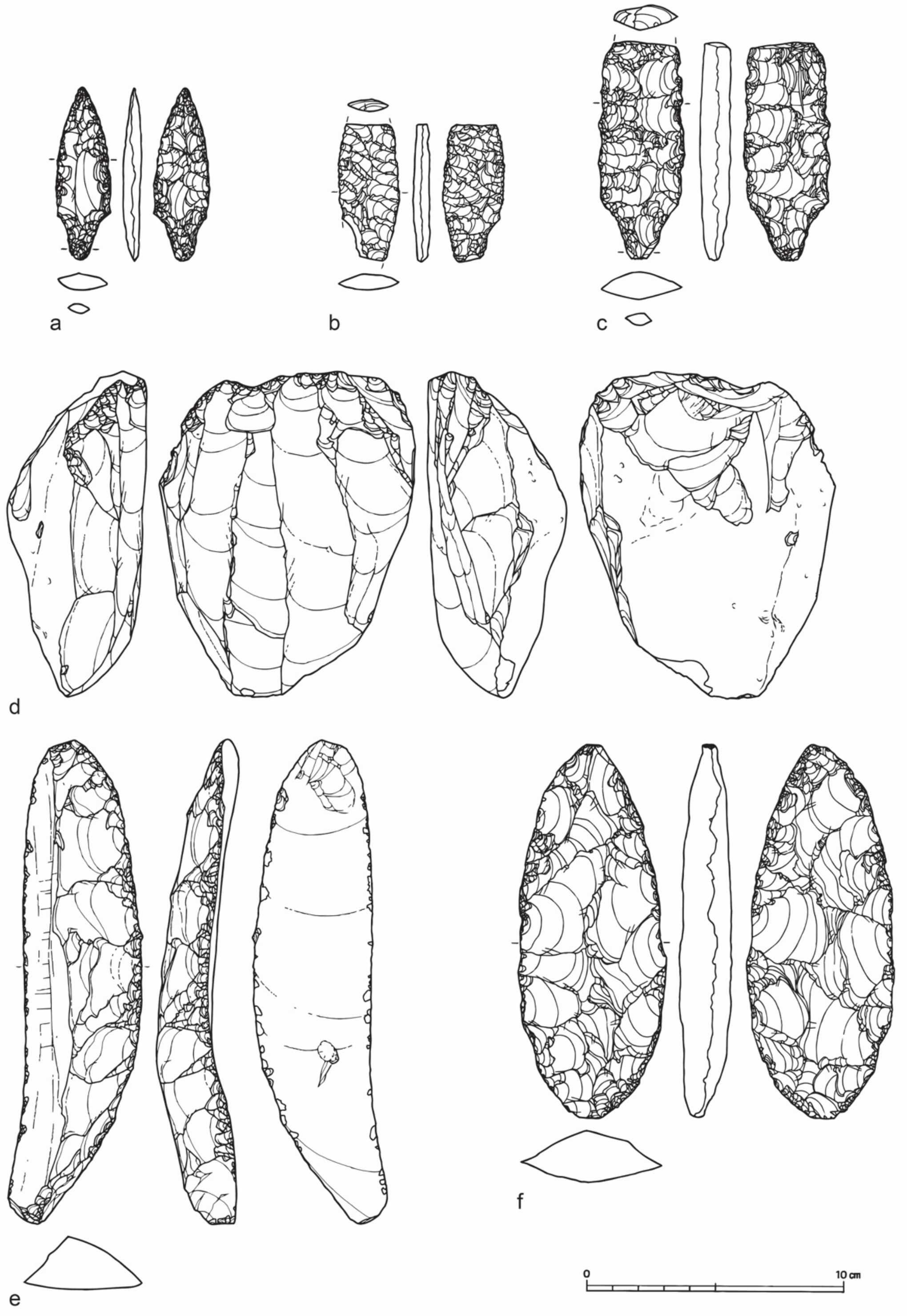

Fig. S12. Late Upper Paleolithic artifacts from the Okushirataki 1 site, Hokkaido, Japan. Illustrated examples of bifacial stemmed points (a-c), prismatic blade core (d), prismatic blade tool (e), and biface (f) technology excavated from the Okushirataki 1 site's SB-53 archaeological component dating to ~21,400-19,830 cal yr B.P. from CB-19 (redrawn from *9*).

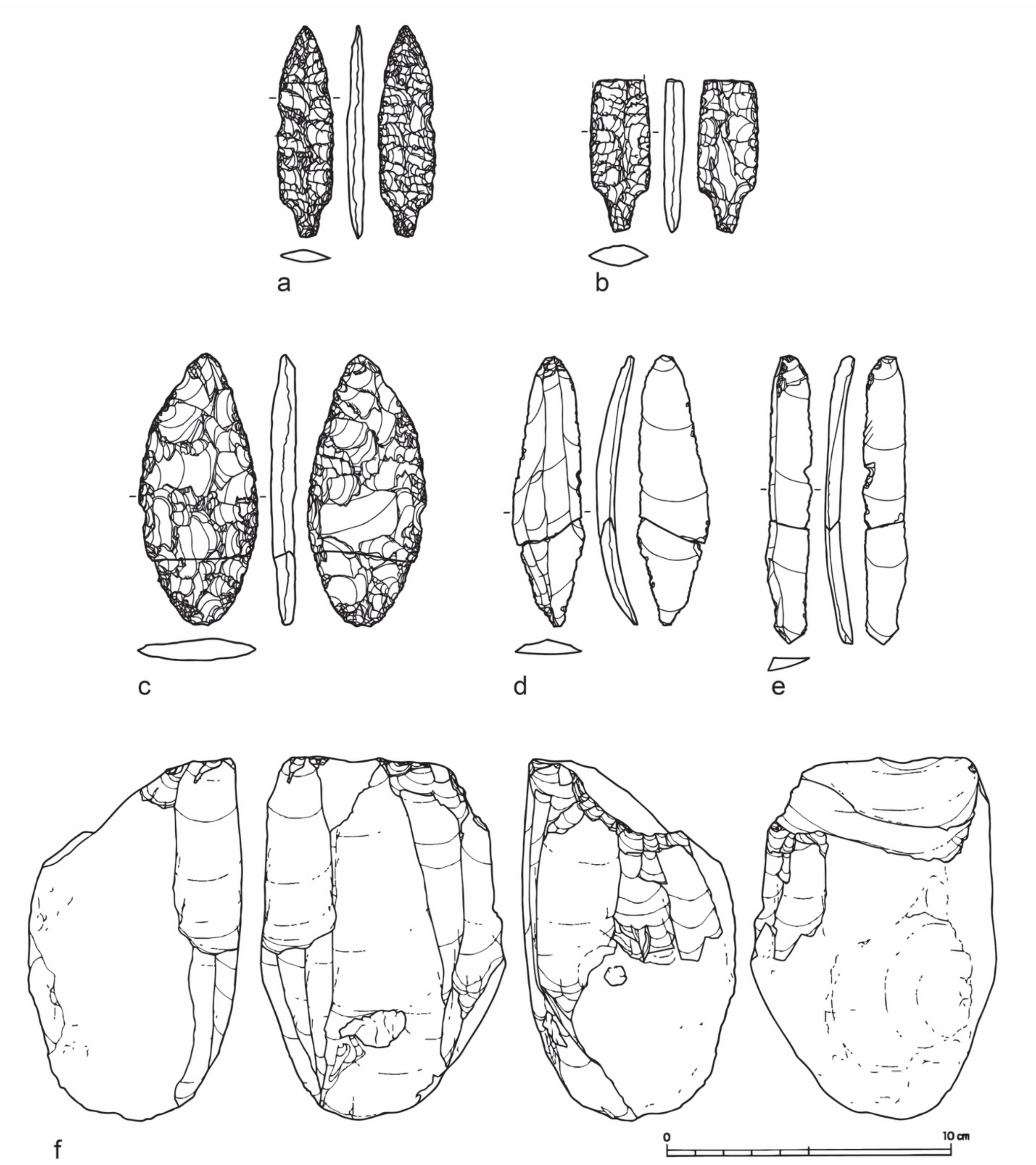

Fig. S13. Late Upper Paleolithic artifacts from the Hattoridai 2 site, Hokkaido, Japan. Illustrated examples of bifacial stemmed points (a, b), biface (c), prismatic blades (d, e), and prismatic blade core (f) technology excavated from the Hattoridai 2 site's SB-38~40 archaeological component, dating to ~16,750-16,170 cal yr B.P. from CB-5 (redrawn from *9*).

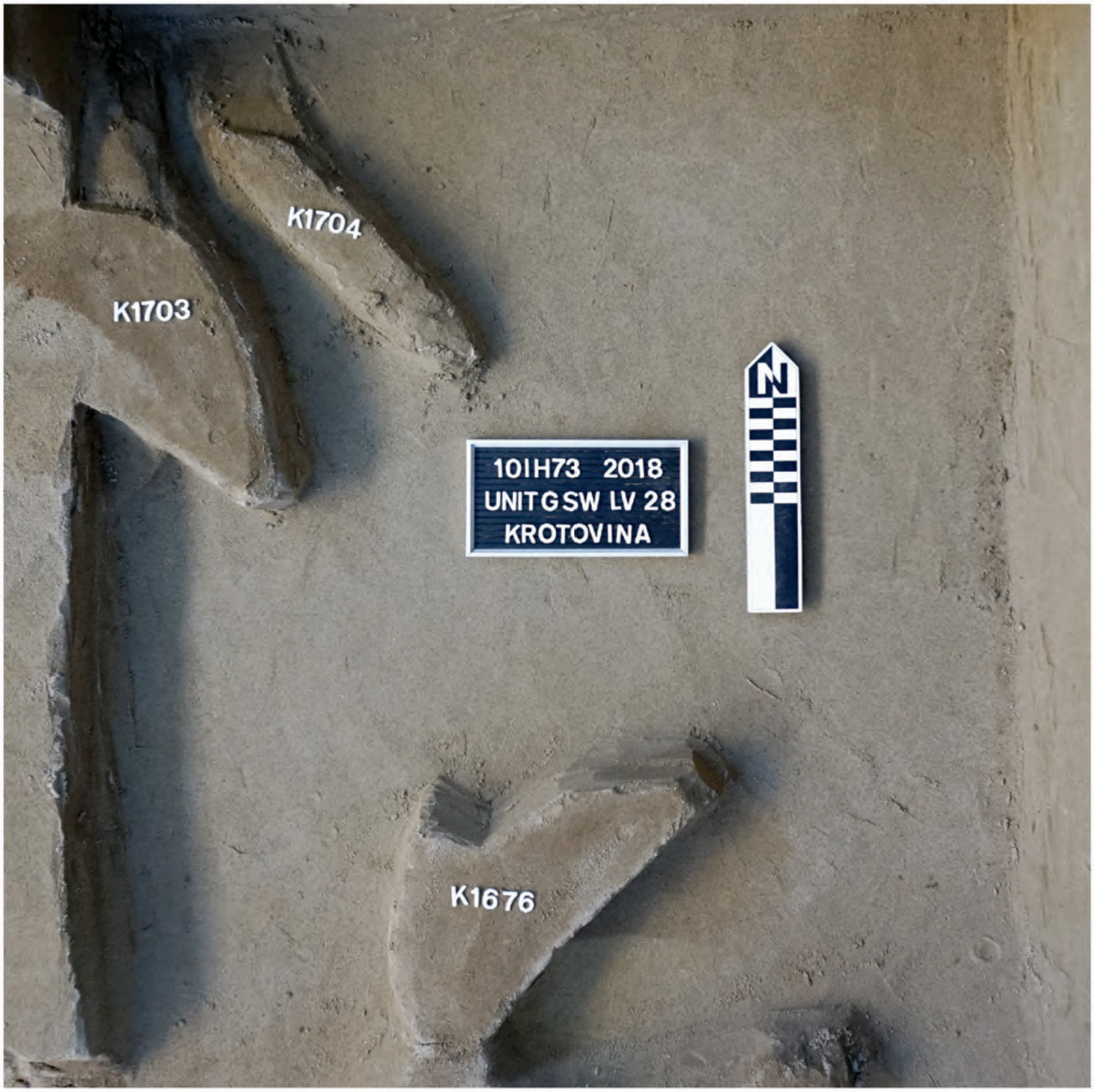

Fig. S14. Examples of krotovina traces. Krotovina (rodent burrows) from the Cooper's Ferry site were defined and excavated separately from undisturbed sediments.

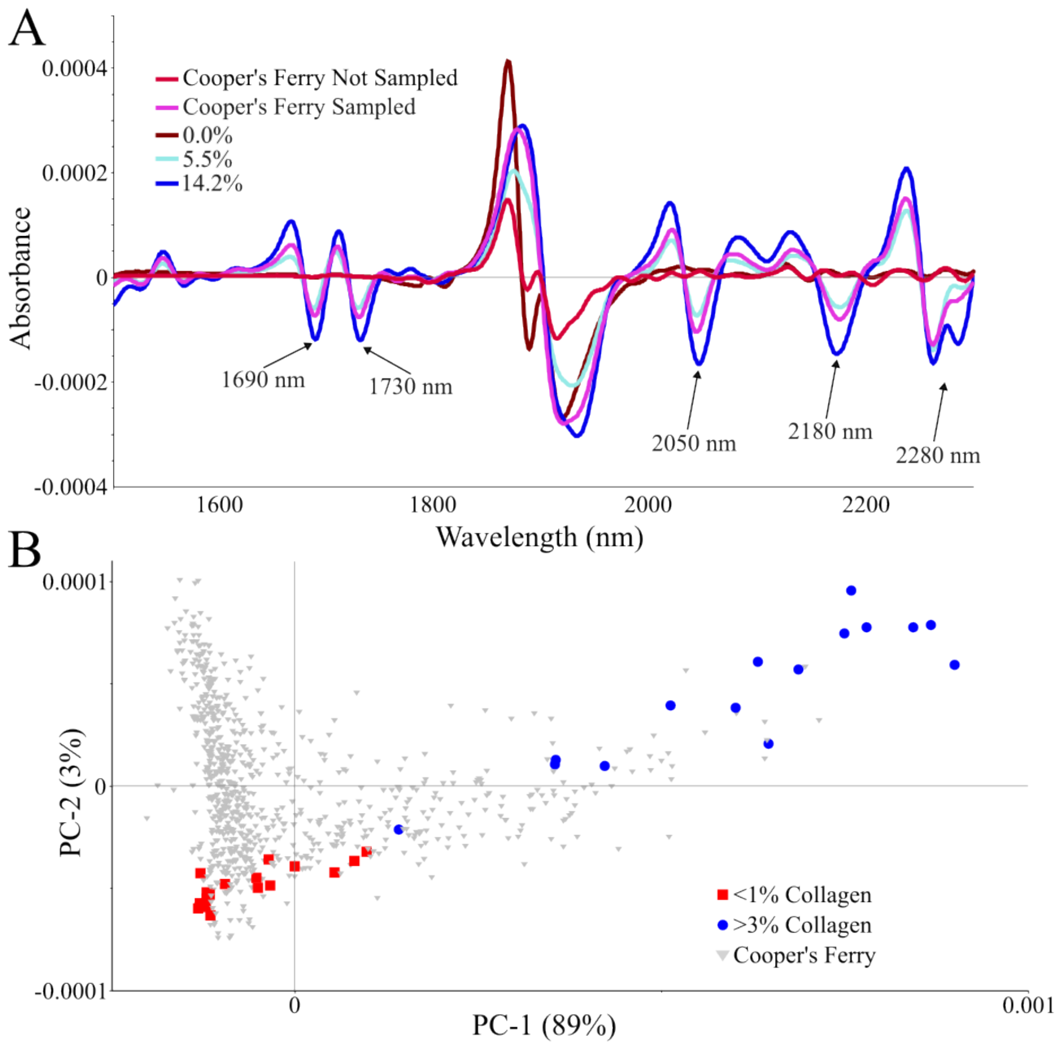

Figure S15. NIR bands reflect collagen content. A) Absorbance spectra (second derivative; 31 points smoothing) from known archaeological specimens yielding 0.0% collagen (maroon), 5.5% collagen (turquoise), and 14.2% collagen (blue), and two specimens from Cooper's Ferry–one sampled (fuchsia) and one unsampled (red). The unsampled Cooper's Ferry specimen showed strong similarities with archaeological specimens lacking collagen in bands/regions reflecting collagen content (arrows at 1690 nm, 1730 nm, 2050 nm, 2180 nm, and 2280 nm) and was therefore not sampled for radiocarbon dating. The sampled Cooper's Ferry specimen, in contrast, retained clear evidence of collagen preservation–possibly greater than 5%--and was therefore an ideal candidate for radiocarbon dating. B) PCA scores plot (PC1 and PC2) of the collagen-related regions of the NIR absorbance spectra (1671-1751 nm, 2030-2060 nm, 2153-2200 nm, 2250-2303 nm; second derivative; 31 points smoothing) of 32 known larger whole bone samples from archaeological sites and 776 other smaller Cooper's Ferry samples (gray triangles). Among the known archaeological samples, specimens with more than 3% collagen (blue circles) separate from low collagen specimens (<1%; red squares). Along PC1 (89% of variation), Cooper's Ferry samples typically cluster with specimens containing <1% collagen. Only 12.2% of the Cooper's Ferry samples were deemed good candidates for AMS radiocarbon dating (those within the blue cluster). Only 4.6% of the samples appeared sufficiently well preserved to make successful collagen extraction almost certain (those nearer the blue group's centroid).

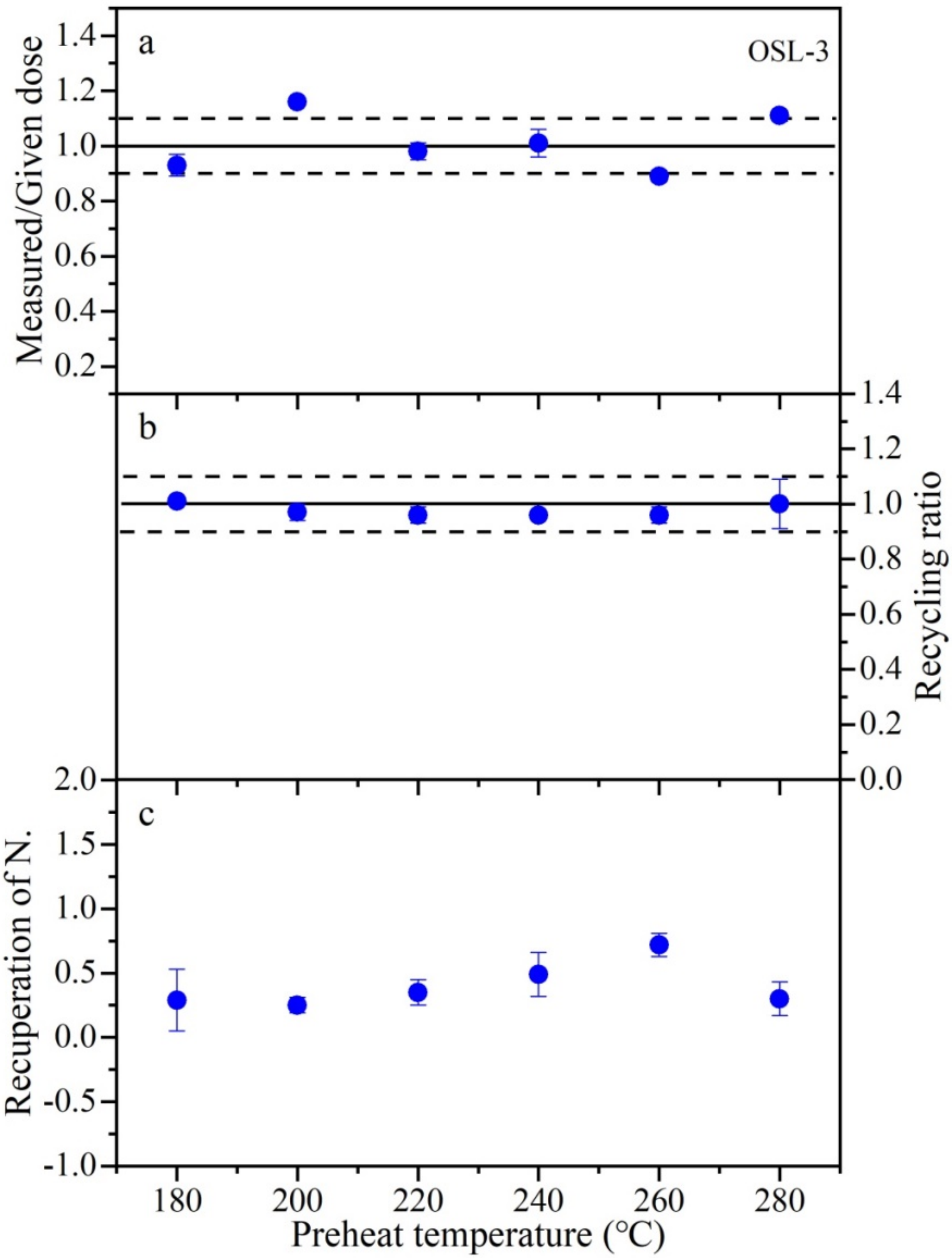

Fig. S16. Preheat plateau test results for a quartz test sample.

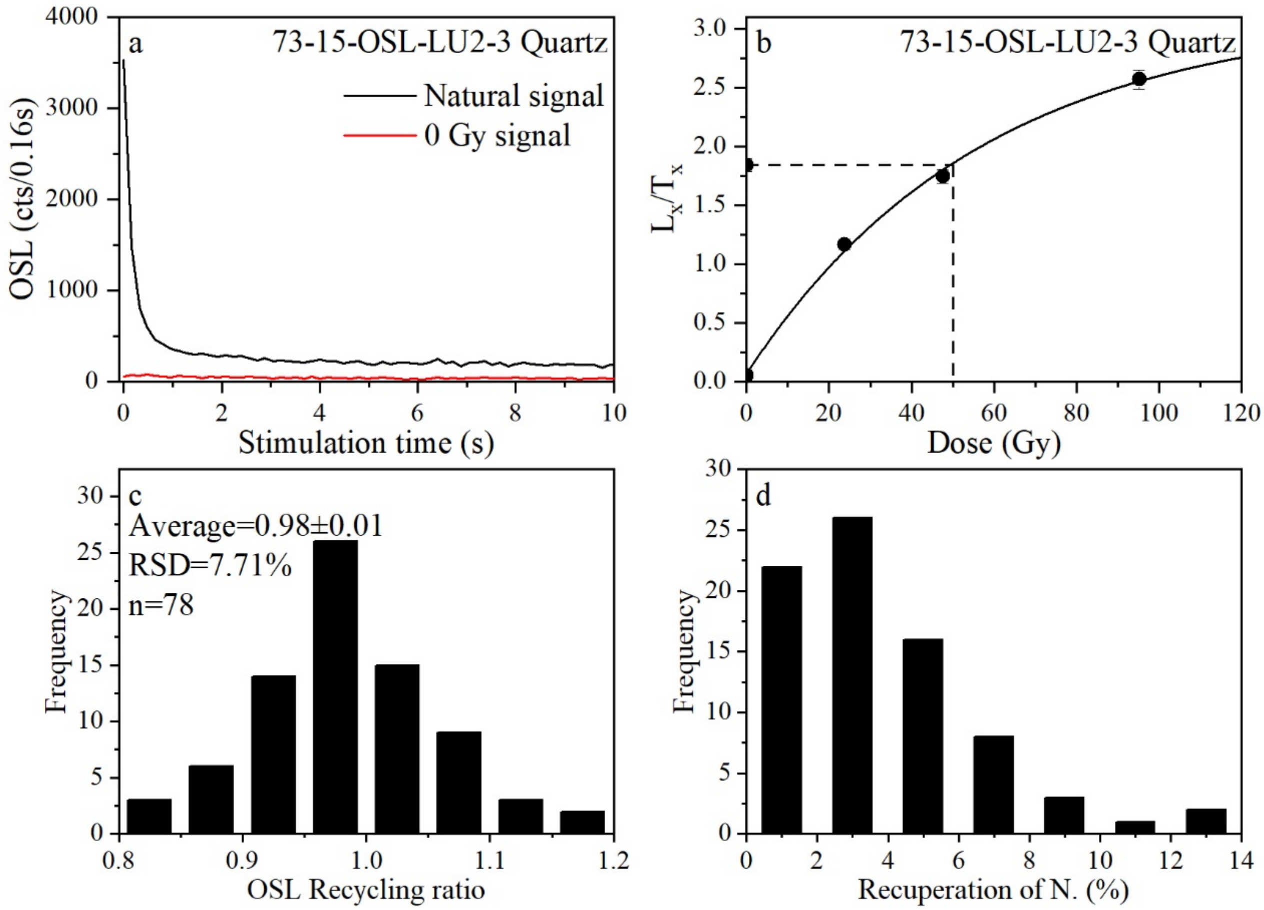

Fig. S17. Luminescence characteristics of quartz test samples; (a) and (b) are the decay and growth curves of the OSL signal for sample 73-15-OSL-LU2-3; (c) and (d) are the recycling ratios and recuperation values of all the quartz aliquots.

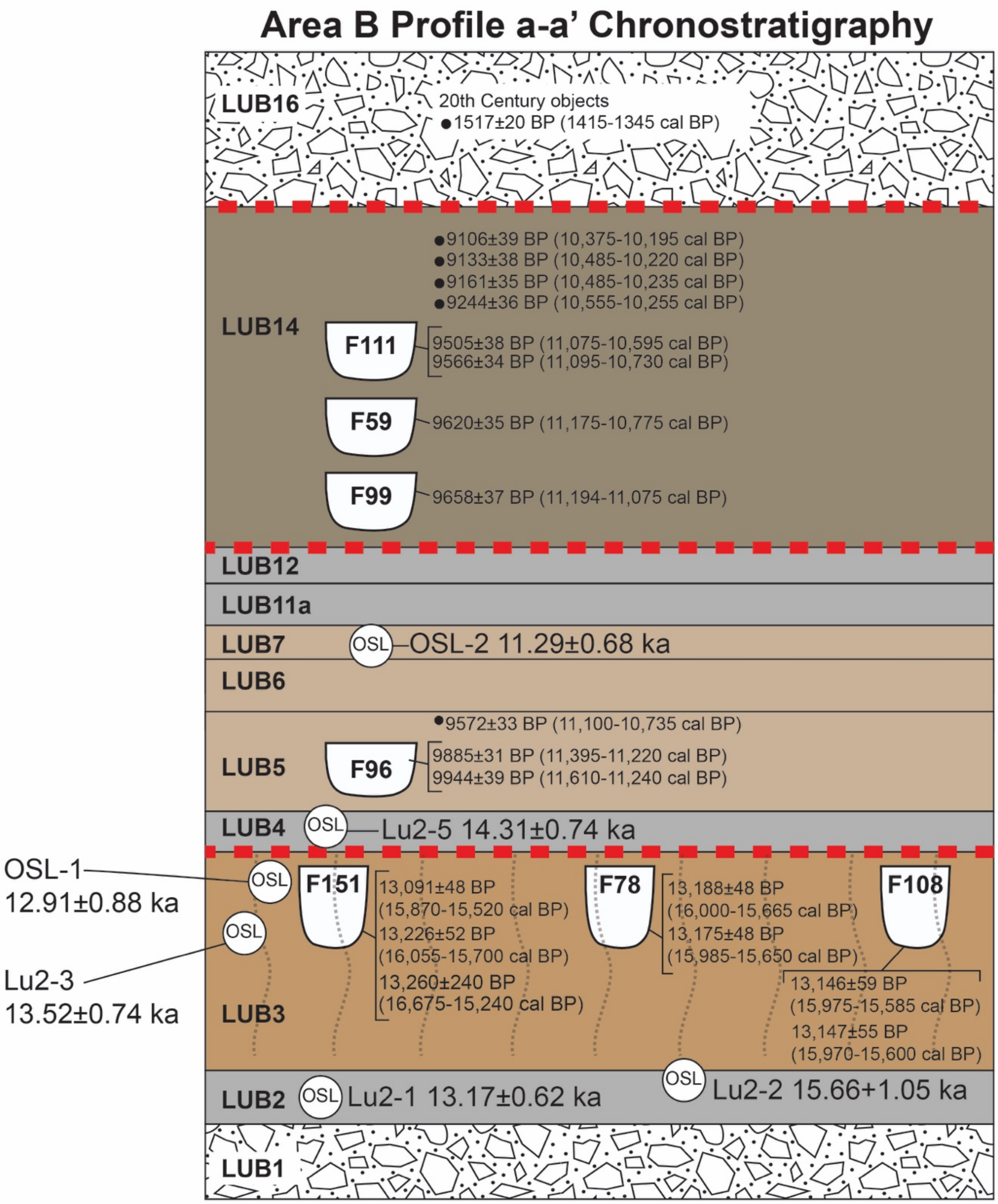

Fig. S18. Combined Area B ^{14}C and OSL chronostratigraphy using a 30% water content estimate for the OSL samples.

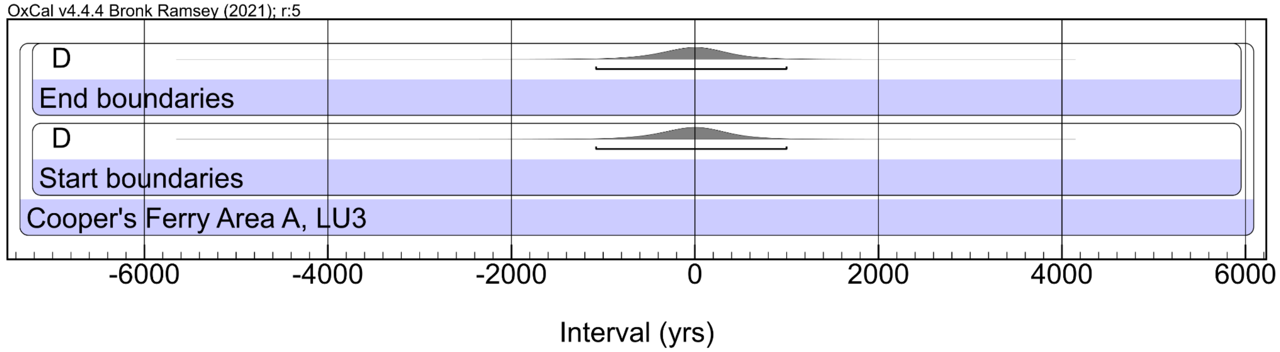

Fig. S19. Probability density functions for the difference (“D”) between the start and end boundaries of LU3, as estimated by Davis et al. (*1*) and in this analysis. These results suggest that there is 95.4% chance that the modelled outputs have no significant difference.

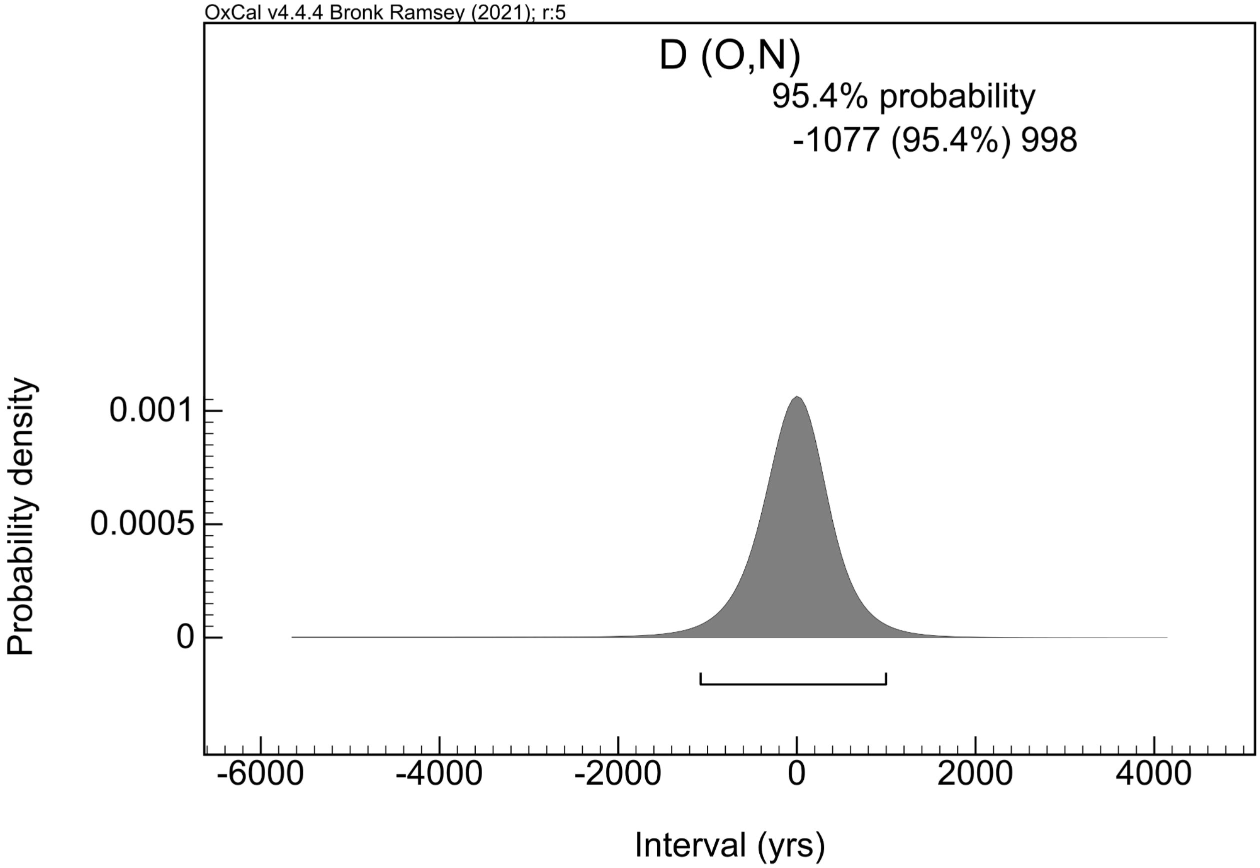

Fig. S20. Probability density function for the difference ("D") between the start of Area A LU3 ("O"; from model in Fig. 5) and Area B LUB3 ("N", from model in Fig. 6). These results suggest that there is 95.4% chance that the modelled outputs have no significant difference.

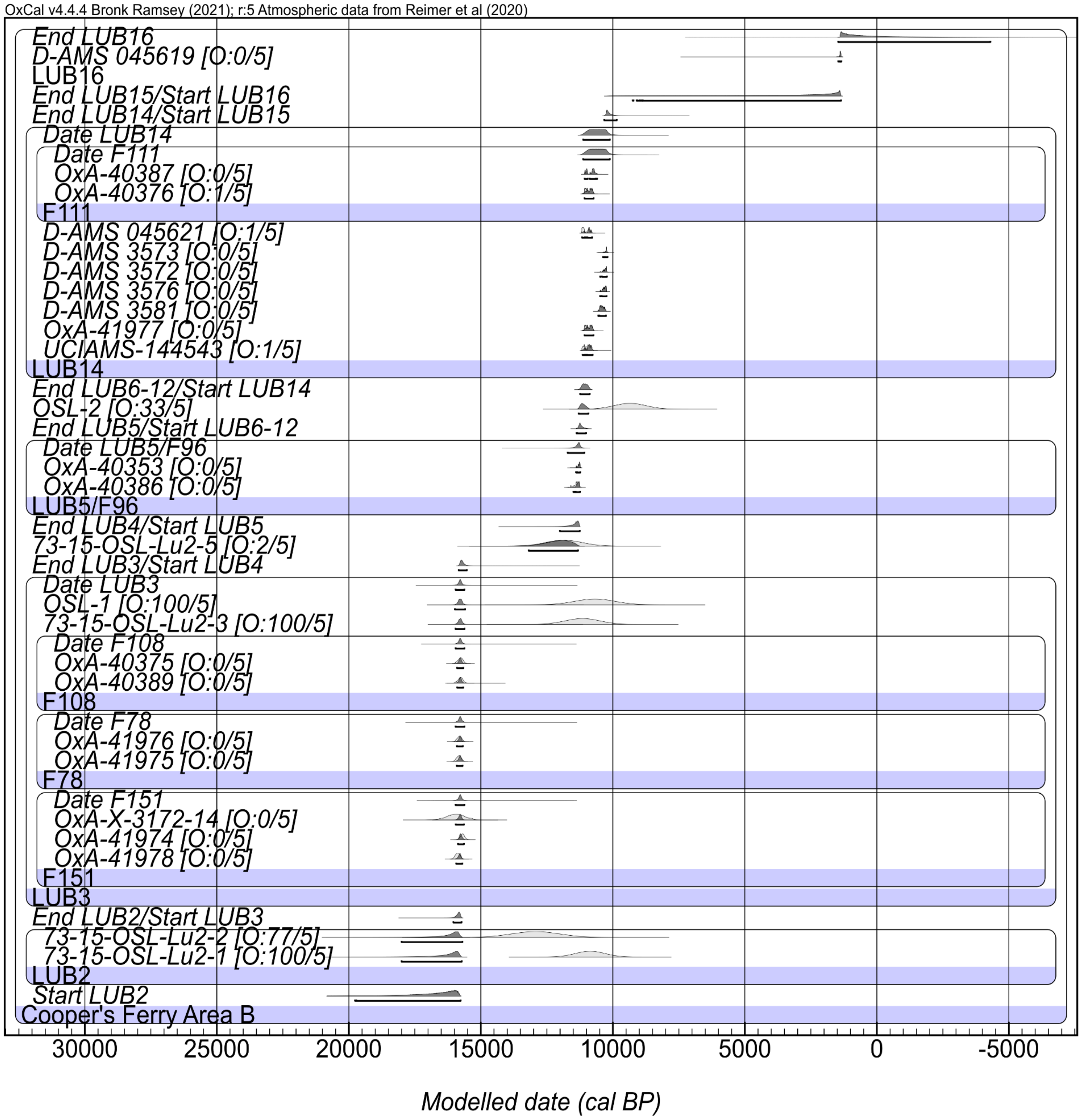

Fig. S21. Bayesian model for Cooper's Ferry Area B, including OSL ages calculated with a 10±5% water content (Model "B"). This estimates the start of LUB3 at 16,040-15,725 cal yr B.P. Outlier analysis output is noted as 'O:posterior probability/prior probability'.

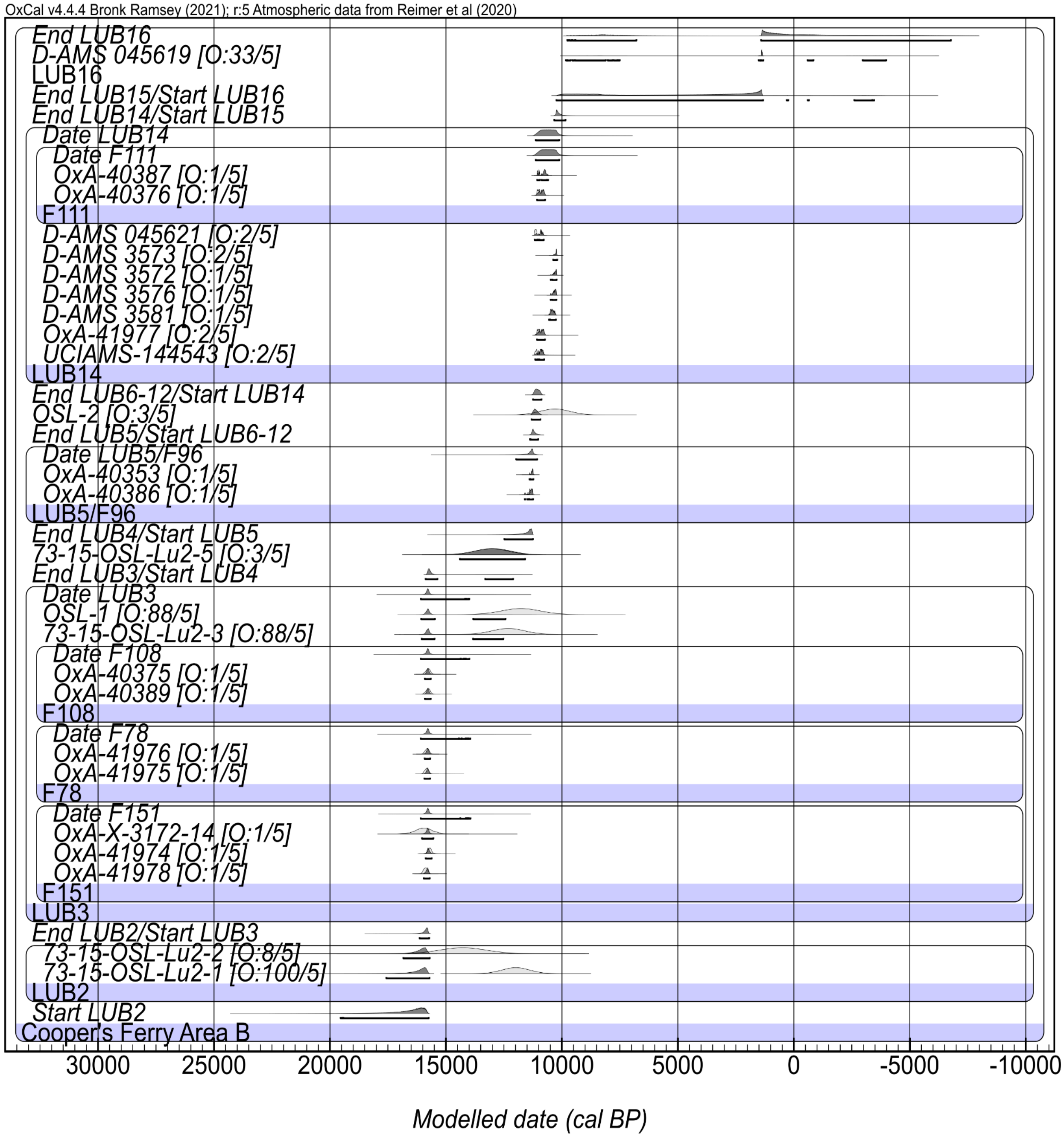

Fig. S22. Bayesian model for Cooper's Ferry Area B, including OSL ages calculated with a 20±5% water content (Model "C"). This estimates the start of LUB3 at 16,140-15,715 cal yr B.P. Outlier analysis output is noted as 'O:posterior probability/prior probability'.

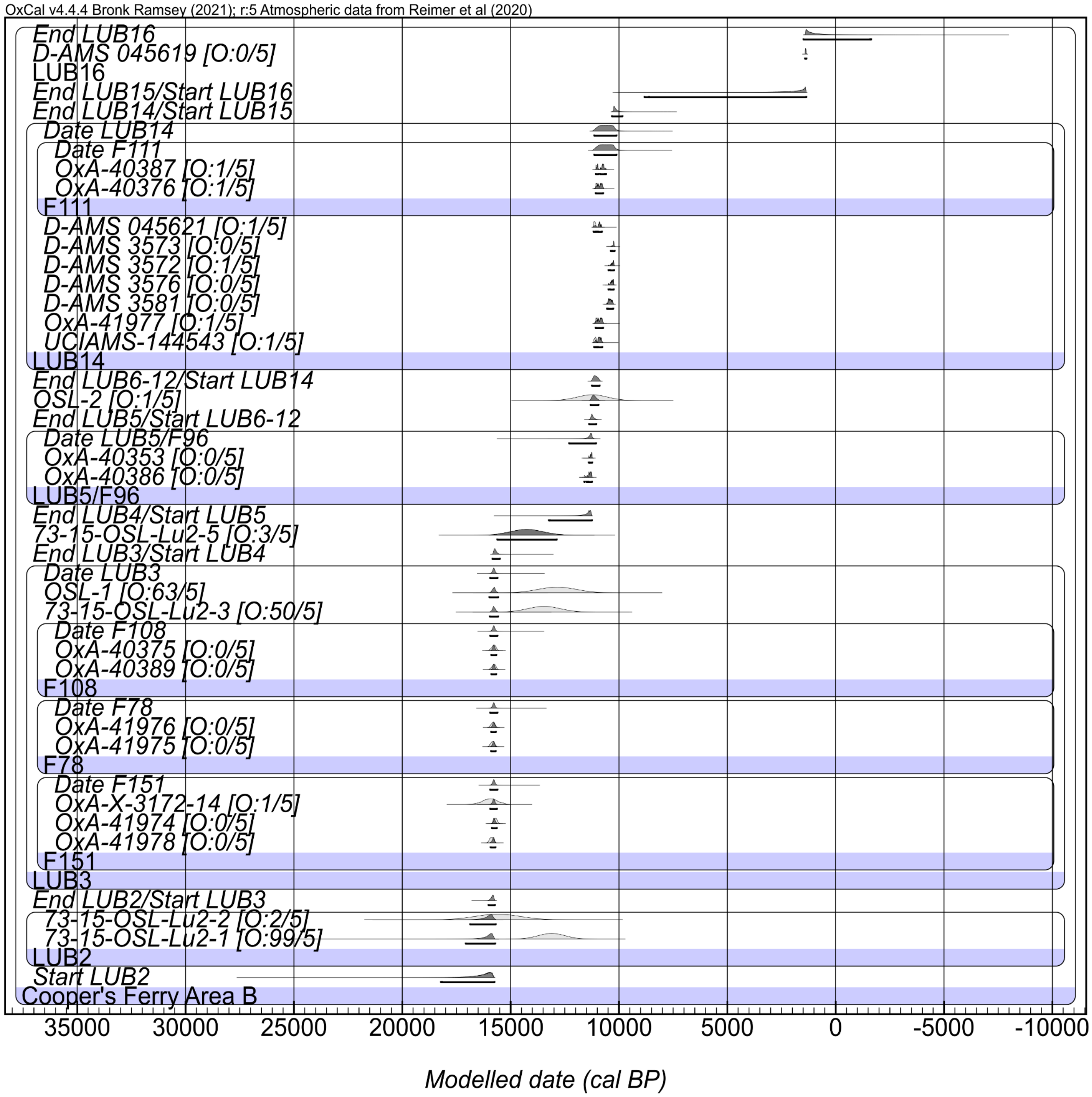

Fig. S23. Bayesian model for Cooper's Ferry Area B, including OSL ages calculated with a 30±5% water content (Model "D"). This estimates the start of LUB3 at 16,020-15,725 cal yr B.P. Outlier analysis output is noted as 'O:posterior probability/prior probability'.

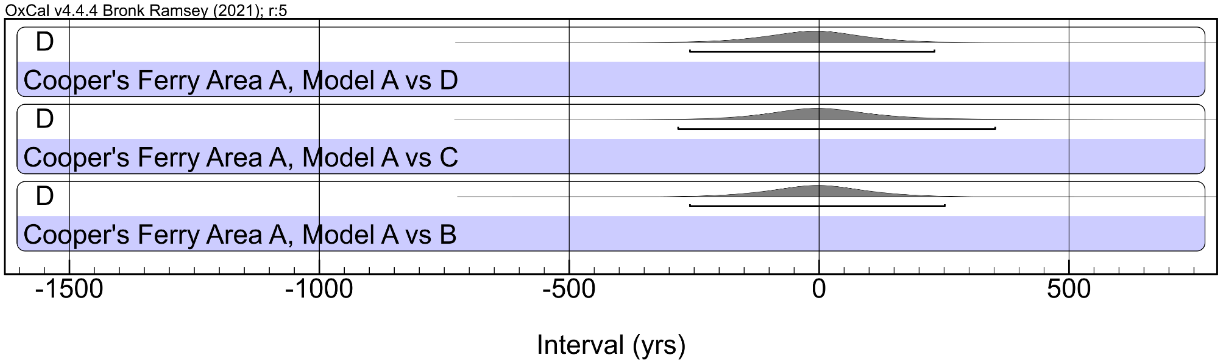

Fig. S24. Probability density functions for the difference ('D') between the start of Area A LU3 in Model A (Fig. 5) versus the same in Models B-D (figs. S20-S22). These results suggest that there is 95.4% chance that the modelled outputs have no significant difference.

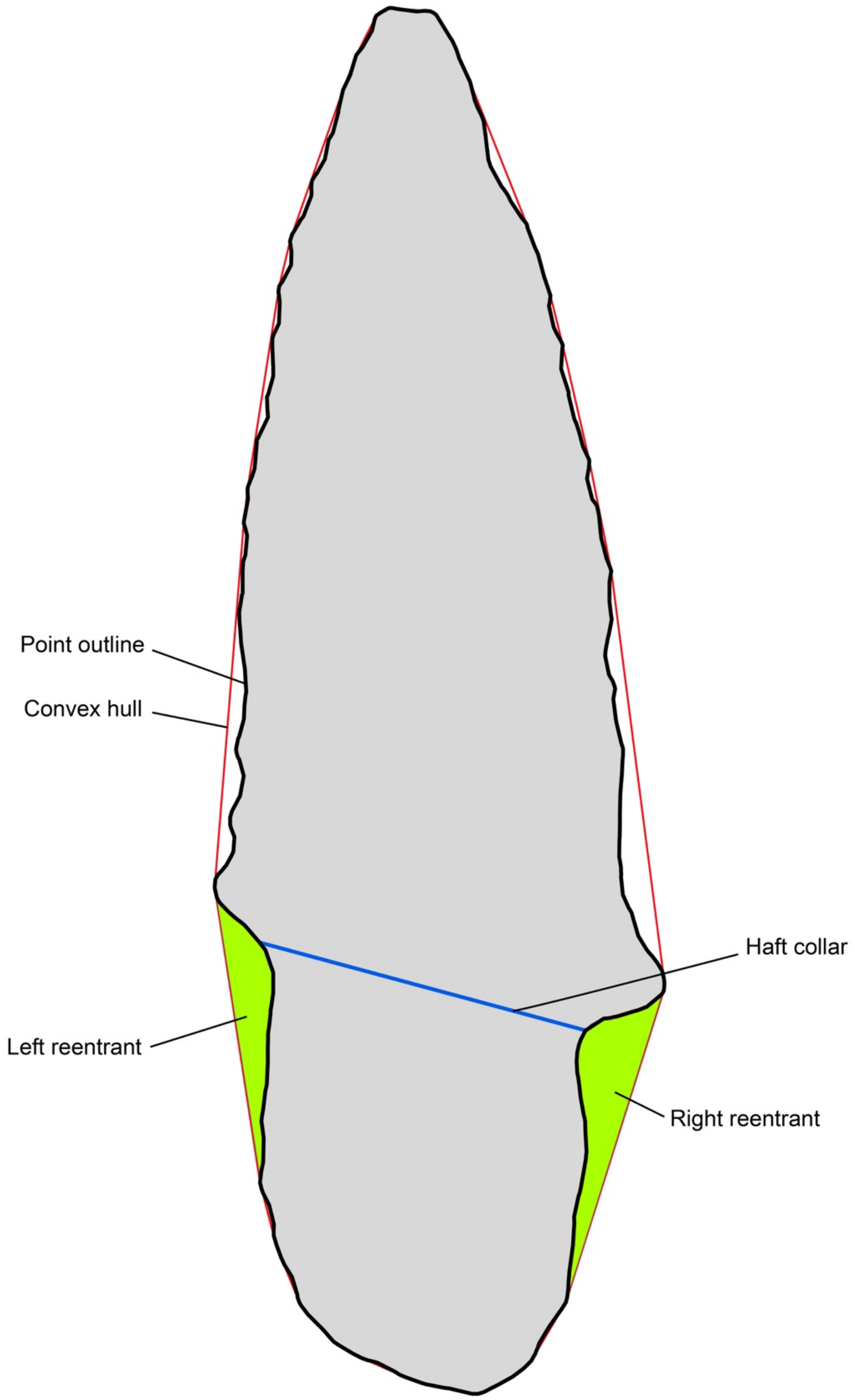

Figure S25. Projectile point reentrants and their morphometric elements as measured by GLiMR (*66*). Reentrants are the negative spaces between the convex hull (i.e., the smallest convex polygon that contains all the artifact scan's XY spatial points) and the artifact's outline (*72,73*). In the case of flaked stone tools, reentrants commonly represent areas of material removal along margins (e.g., serrations, denticulations, stemmed hafts, and notches).

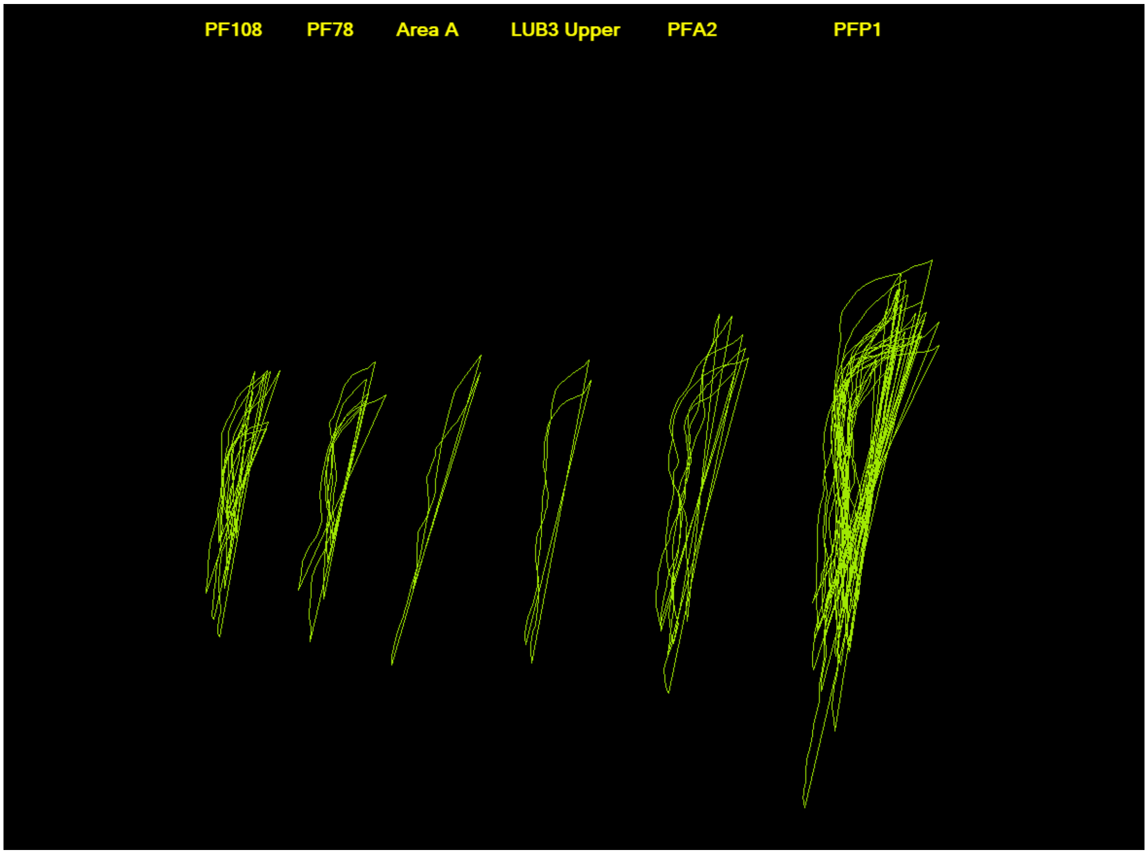

Figure S26. Outlines of haft reentrants measured from 3D digital models of late Pleistocene-aged stemmed projectile points at the Cooper's Ferry site in the Pit Feature 108, Pit Feature 78, Area A (point 73-60685 (*1*)), upper LUB3 (point 73-54105; Fig. 4), Pit Feature A2 (*42*), and Pit Feature P1 (*43*). The left-side reentrant has been mirrored about the long axis of the point to allow better comparison with the right-side reentrant. Therefore, there are two similarly oriented reentrants shown for each projectile point. Reentrants are organized from oldest (PF108 at left) to youngest (PFP1 at right) assemblages.

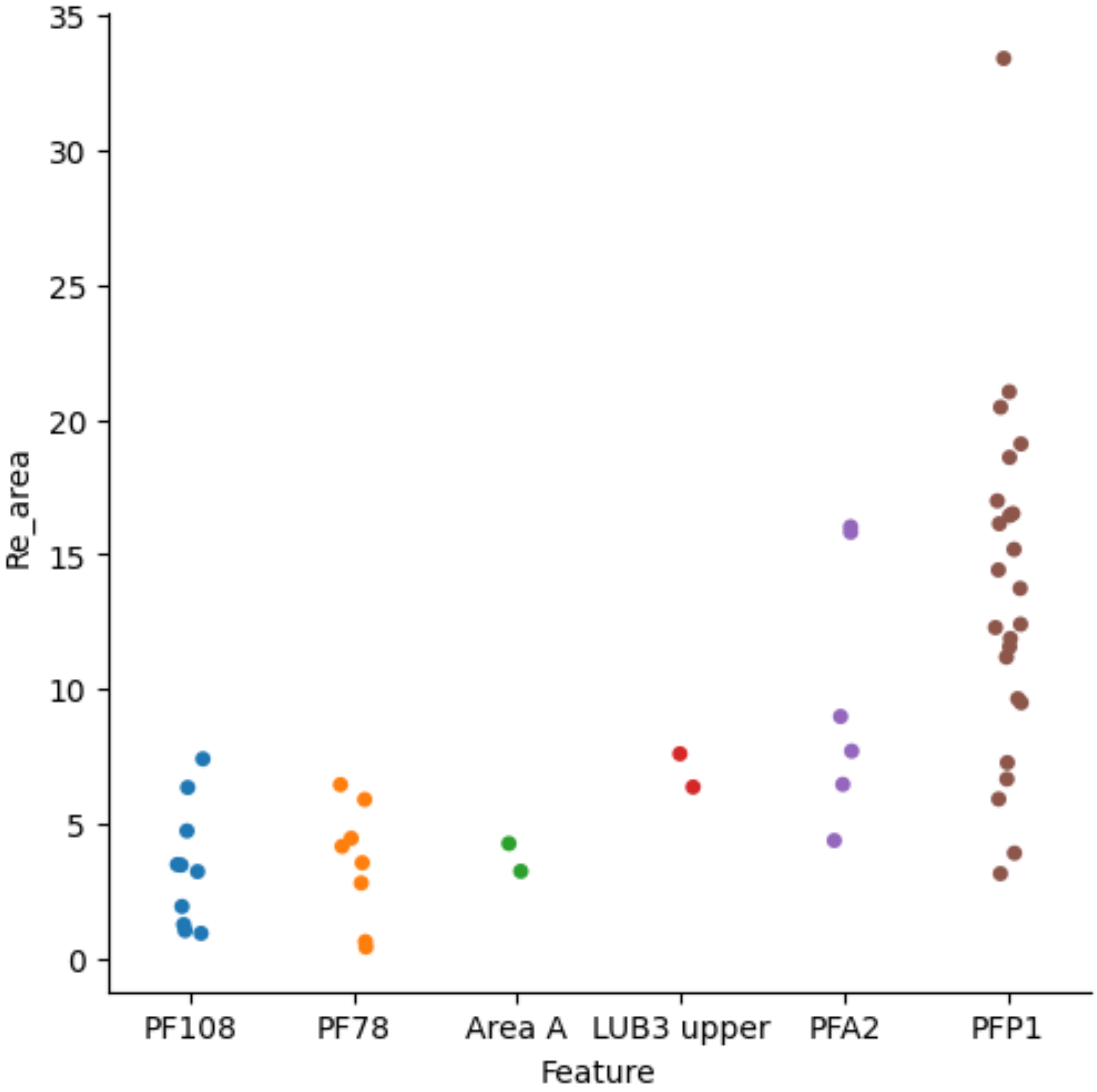

Figure S27. Correlation of haft reentrant area with projectile point assemblage. Plotted data points represent left and right-side haft reentrant areas (Re_area) in square millimeters. Feature 108 (PF108), Feature 78 (PF78), Area A (point 73-60685 (*1*)), LUB3 (73-54105; Fig. 4), Pit Feature A2 (PFA2) (*42*), and Pit Feature P1 (PFP1) (*43*). Reentrants are organized from oldest (PF108 at left) to youngest (PFP1 at right) assemblages.

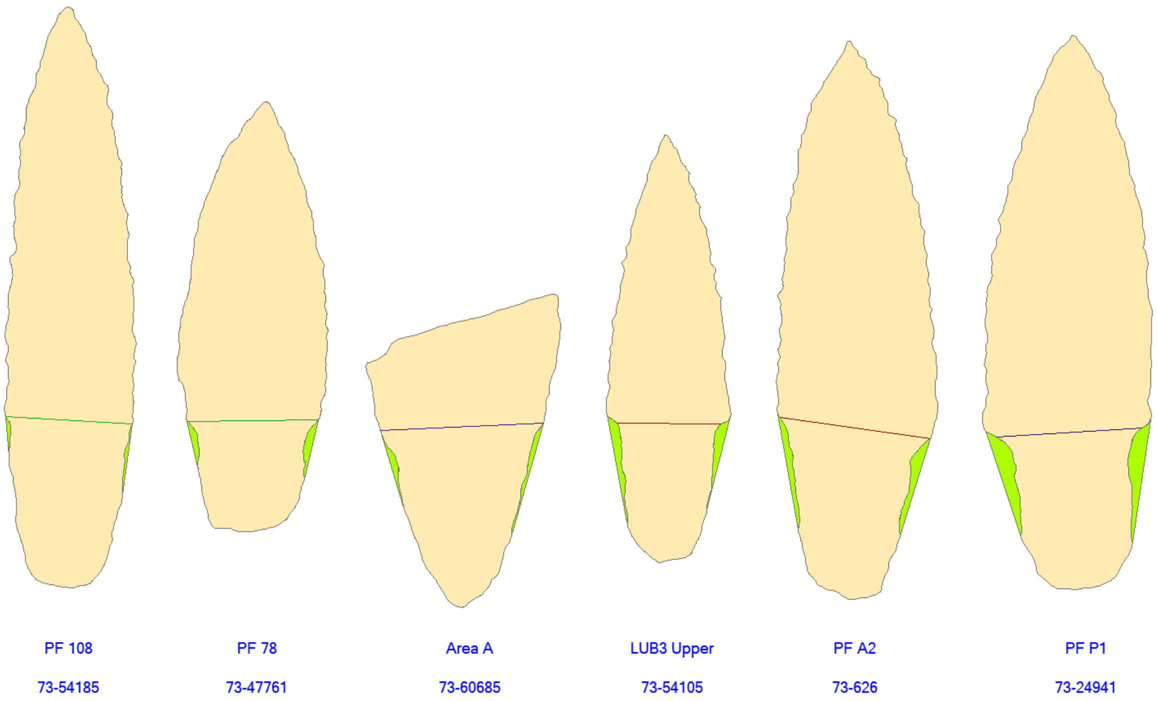

Fig. S28. Reentrant shapes (green polygons) measured on representative projectile points from Feature 108 (PF 108), Feature 78 (PF 78), Area A (i.e., LU3), the upper portion of LUB3, Pit Feature A2 (PFA2), and Pit Feature P1 (PFP1). The near-horizontal line separates the haft and blade elements. The point images are arranged in chronological order, from oldest (at left) to youngest (at right).

Table S1. Stratigraphic description of deposits exposed along the a-a' transect, Area B.

LUB16: A thick deposit of subangular to angular pebbly and cobbly brown (10YR 4/3 moist; 10YR 5/3 dry) fine sandy loam, dark grayish-brown (10YR 4/2 moist; 10YR 6/2 dry) sandy loam, and yellowish brown (10YR 5/4 moist; 10YR 7/1 dry) sand. This unit retains irregular and discontinuous internal beds and massive sediments that contain precontact and historic period artifacts mixed together. These are disturbed sediments created by mechanical excavation and backfilling of stratigraphically lower sediments. The lower boundary of this unit is sharp, irregular, and unconformable.
LUB14: Brown (10YR 4/3; 10YR 5/3 dry) massive, fine pebbly loamy sand. This deposit is contained in intrusive channel features that eroded into underlying LUB12, LUB11a, LUB5 sediments. The lower boundary of the unit is unconformable with a sharp and irregular form.
LUB12: Pale brown (10YR 6/3 moist; 10YR 7/2 dry), massive, moderately well-sorted sand without visible structures. Mica flakes and biotite accompany a large percentage of quartzitic and plagioclase sands. The lower boundary of this unit is sharp and smooth.
LUB11a: Brown (10YR 5/4 moist; 10YR 7/1 dry), massive, moderately well-sorted sand without visible structures. Mica flakes and biotite accompany a large percentage of quartzitic and plagioclase sands. The lower boundary of this unit is sharp and smooth.
LUB7: Yellowish brown (10YR 5/4; 10YR 7/1 dry), massive, fine loamy sand with a clear wavy conformable lower boundary. Rare calcium carbonate is diffused throughout matrix. Clear, wavy boundary.
LUB6: Brown (10YR 5/3 moist; 10YR 7/2 dry), massive, fine sandy loam with friable consistence. Rare calcium carbonate is diffused throughout matrix. Clear, wavy boundary.
LUB5: Yellowish brown (10YR 5/4; 10YR 7/1 dry), massive, fine loamy sand with a clear wavy conformable lower boundary. Rare calcium carbonate is diffused throughout matrix. Clear, wavy boundary.
LUB4: Yellowish brown (10YR 5/4 moist; 10YR 8/1 dry), massive, moderately well-sorted sand. This lowermost sand has a sharp irregular basal boundary marking an erosional unconformity.
LUB3: (S1) Brown (10YR 5/3; 10YR 6/3 dry), massive, fine sandy loam with friable to firm consistence, bounded below by a clear conformable boundary. Calcium carbonate horizon is present as fine filaments, hypocoatings, and forms pendants on the undersides of artifacts and bone fragments throughout the deposit. This unit bears the Rock Creek Paleosol that shows erosional truncation at its upper surface shared with LUB4.
LUB2: Yellowish brown (10YR 5/4 moist; 10YR 8/1 dry), massive, moderately well-sorted sand. Calcium carbonate appears as dispersed hypocoatings and fine filaments throughout the

sediment matrix. This lowermost sand has a sharp irregular basal boundary that appears to be conformable.
LUB1: Subrounded to subangular basalt clasts of fine pebble to fine boulder size with no apparent bedding structure in a relatively poorly sorted, clast-supported matrix. Carbonates coat the undersides of clasts in some areas. Lower boundary was not observed.

Table S2. Quantities of artifacts in LUB3 and early pit features in Area B, and LU3 in Area A. FCR = fire cracked rock. MF = modified flake. PPT = projectile point.

Context	Biface	Blade	Burin	Core	Debitage	FCR	MF	PPT	Artifact Subtotal
F78	1	0	1	1	250	7	0	5	265
F108	0	0	0	0	53	0	0	7	60
F151	0	0	0	0	8	0	0	0	8
LUB3	0	0	0	0	10	0	0	2	12
Subtotals	1	0	1	1	321	7	0	14	345
LU3	4	2	0	2	161	1	19	2	191
Subtotals	4	2	0	2	161	1	19	2	191
**Totals**	**5**	**2**	**1**	**3**	**482**	**8**	**19**	**16**	**536**

Table S3. Quantities of faunal materials and charcoal in LUB3 and early pit features in Area B, and LU3 in Area A. MShell = freshwater mussel shell.

Context	Bone	MShell	Faunal Subtotal	Charcoal
F78	226	0	226	2
F108	21	0	21	0
F151	16	0	16	0
LUB3	6	0	6	0
Subtotals	269	0	269	2
LU3	84	1	85	42
Subtotals	84	1	85	42
**Totals**	**353**	**1**	**354**	**44**

Table S4. AMS radiocarbon ages from Area A. RN is the reading number. The percent collagen is the yield of extracted collagen as a function of the starting weight of bone samples. C:N is the atomic weight ratio of carbon to nitrogen. %C is the percentage of carbon in the combusted sample. Stable isotope ratios of C and N are expressed in per mil (‰) relative to Vienna Pee Dee belemnite and ambient inhalable reservoir. The calibrations were done using the OxCal 4.3 software (*59*) and the IntCal20 calibration curve (*58*). Missing chronometric data (*) are due to a lack in reporting or measurement on behalf of the laboratories. CI, confidence interval; –, not determined.

RN	Laboratory no.	Material	Northing (m)	Easting (m)	Elevation (masl)	LU	% collagen	C:N	%C	δ13C (‰)	δ15N (‰)	yr B.P	± 1 SD	cal yr B.P. (95.4% CI)
136	D-2070	mussel shell	80.496	107.244	413.143	6	-	-	*	-13	-	8749	43	9895-9555
156	D-2071	mussel shell	80.193	106.138	413.085	6	-	-	*	-16.2	-	8229	36	9400-9027
177	D-2072	mussel shell	79.815	106.820	412.942	6	-	-	*	-8.2	-	8773	36	10,110-9557
20384	D-1875	mussel shell	79.78	107.678	412.853	6	-	-	*	-4.6	-	8776	38	10,113-9557
20691	D-1888	mussel shell	79.943	107.688	412.851	6	-	-	*	-15.6	-	8617	35	9679-9532
20381	D-1874	mussel shell	79.775	107.372	412.848	6	-	-	*	-0.5	-	8635	38	9685-9535
11157	D-1880	mussel shell	79.929	107.588	412.847	6	-	-	*	-0.6	-	8814	39	10,146-9686
20860	D-1890	mussel shell	80.113	107.299	412.847	6	-	-	*	5.4	-	9138	38	10,482-10,226
11154	D-1879	mussel shell	79.855	107.472	412.844	6	-	-	*	-11.7	-	8577	37	9659-9481
20584	D-1886	mussel shell	79.576	107.347	412.842	6	-	-	*	-7	-	8831	34	10,147-9707
11153	D-1878	mussel shell	79.824	107.455	412.841	6	-	-	*	0.8	-	8563	30	9550-9486
11149	D-1877	mussel shell	79.598	107.660	412.841	6	-	-	*	2.4	-	8624	31	9677-9534
20385	D-1876	mussel shell	79.787	107.722	412.841	6	-	-	*	4.4	-	8687	28	9700-9545
11162	D-1881	mussel shell	79.875	107.821	412.837	6	-	-	*	-5.6	-	8671	31	9690-9543
20583	D-1885	mussel shell	79.609	107.359	412.837	6	-	-	*	-13.1	-	8874	39	10,179-9778
20635	D-1887	mussel shell	79.554	107.365	412.833	6	-	-	*	-16.9	-	8935	37	10,200-9908
11767	D-1892	mussel shell	80.435	106.128	412.829	6	-	-	*	-26.2	-	8721	38	9889-9547
20577	D-1884	mussel shell	79.691	107.258	412.828	6	-	-	*	-16.8	-	8716	36	9889-9546
20572	D-1882	mussel shell	79.766	107.294	412.828	6	-	-	*	-5.3	-	8783	34	10,114-9561
20576	D-1838	mussel shell	79.724	107.264	412.826	6	-	-	*	-7.9	-	8635	33	9679-9537
20294	D-1873	mussel shell	79.458	107.521	412.818	6	-	-	*	-5.8	-	8676	33	9702-9541
20181	D-1600	mussel shell	79.521	107.129	412.813	6	-	-	*	14.2	-	8792	38	10,119-9606

20188	D-1605	mussel shell	79.387	107.345	412.810	6	-	-	*	-4.5	-	8848	34	10,156-9743
21763	D-1893	mussel shell	79.938	107.433	412.804	6	-	-	*	-3	-	8666	31	9687-9542
21764	D-1895	mussel shell	79.936	107.420	412.804	6	-	-	*	-16.5	-	8680	36	9729-9541
21764	D-1894	mussel shell	79.936	107.420	412.804	6	-	-	*	-6.7	-	8783	36	10,115-9560
20186	D-1603	mussel shell	79.341	107.324	412.804	6	-	-	*	-0.3	-	8884	44	10,186-9779
20187	D-1604	mussel shell	79.37	107.355	412.800	6	-	-	*	-2.2	-	8740	33	9890-9554
10718	D-1867	mussel shell	79.412	107.199	412.799	6	-	-	*	-19.1	-	8624	33	9677-9534
10718	D-1868	mussel shell	79.412	107.199	412.799	6	-	-	*	-14.9	-	8685	33	9722-9542
20291	D-1872	mussel shell	79.242	107.354	412.798	6	-	-	*	1.9	-	8624	33	9677-9534
11387	D-2620	mussel shell	81.317	107.692	412.797	6	-	-	*	-4.8	-	8687	35	9740-9542
11387	D-1889	mussel shell	81.317	107.692	412.797	6	-	-	*	1	-	8802	41	10,132-9609
10717	D-1608	mussel shell	79.396	107.157	412.797	6	-	-	*	-15.8	-	8884	36	10,182-9800
20183	D-1601	mussel shell	79.399	107.071	412.795	6	-	-	*	-11.7	-	8725	36	9889-9549
10716	D-1607	mussel shell	79.424	107.124	412.794	6	-	-	*	-10.9	-	8704	40	9887-9543
10715	D-1606	mussel shell	79.408	107.088	412.793	6	-	-	*	-11.3	-	8600	35	9678-9495
20184	D-1602	mussel shell	79.45	107.057	412.792	6	-	-	*	-7.2	-	8596	35	9677-9492
12488	D-2068	mussel shell	79.801	107.526	412.791	6	-	-	*	-7.6	-	8833	36	10,149-9707
20286	D-1871	mussel shell	79.223	107.803	412.781	6	-	-	*	-9.7	-	8759	33	9898-9558
20229	D-1870	mussel shell	79.496	107.140	412.774	6	-	-	*	1.7	-	8804	36	10,124-9679
714	D-2080	mussel shell	79.617	107.140	412.772	6	-	-	*	-13.7	-	8831	36	10,148-9702
682	D-2079	mussel shell	79.932	107.206	412.770	6	-	-	*	-7.3	-	8626	33	9677-9535
14000	D-8782	mussel shell	82.057	109.878	412.769	6	-	-	*	-6.7	-	8833	46	10,154-9695
20222	D-1869	mussel shell	79.094	107.985	412.767	6	-	-	*	-5.5	-	8619	35	9678-9532
13520	D-8771	mussel shell	82.817	109.751	412.765	6	-	-	*	2	-	8764	41	10,107-9554
583	D-2077	mussel shell	80.183	107.824	412.764	6	-	-	*	-13.2	-	8802	34	10,120-9679
11367	D-1596	mussel shell	81.507	98.698	412.759	6	-	-	*	-0.6	-	8783	34	10,114-9561
581	D-2078	mussel shell	80.34	107.819	412.758	6	-	-	*	-0.3	-	8725	43	9890-9548
5393	D-2069	mussel shell	82.783	105.744	412.755	6	-	-	*	-15.9	-	8683	40	9761-9540
2647	D-1581	mussel shell	80.077	108.122	412.746	6	-	-	*	-2.2	-	8799	34	10,120-9673

14170	D-8761	mussel shell	82.307	108.631	412.743	6	-	-	*	-2.7	-	8882	39	10,182-9787
5256	D-1598	mussel shell	82.573	105.787	412.742	6	-	-	*	-10.6	-	8778	34	10,111-9560
23305	D-8774	mussel shell	81.189	108.898	412.730	6	-	-	*	-3.1	-	8886	44	10,187-9781
23240	D-8787	mussel shell	81.83	109.320	412.720	6	-	-	*	-5.6	-	8735	41	9892-9551
208	D-2081	mussel shell	79.646	106.370	412.717	6	-	-	*	-1.7	-	8807	31	10,119-9687
3686	D-2075	mussel shell	79.681	108.861	412.714	6	-	-	*	-15	-	8838	41	10,153-9706
5423	D-2074	mussel shell	80.295	109.790	412.713	6	-	-	*	-14.4	-	8845	39	10,155-9724
5223	D-1587	mussel shell	82.564	109.651	412.709	6	-	-	*	-2.3	-	8766	33	9902-9559
14201	D-8762	mussel shell	82.678	109.131	412.709	6	-	-	*	-4.1	-	8802	38	10,126-9668
661	D-2076	mussel shell	80.359	105.791	412.705	6	-	-	*	-12.4	-	8792	36	10,119-9607
4933	D-2073	mussel shell	82.676	109.871	412.702	6	-	-	*	-10.8	-	8692	40	9881-9541
6835	D-1588	mussel shell	82.514	109.445	412.701	6	-	-	*	-11.5	-	8678	33	9705-9541
6817	D-1586	mussel shell	82.883	105.259	412.698	6	-	-	*	-6	-	8728	31	9889-9550
7696	D-1591	mussel shell	79.468	106.687	412.694	6	-	-	*	-2.8	-	8610	33	9678-9530
7602	D-1590	mussel shell	79.373	106.948	412.690	6	-	-	*	-2.8	-	8588	33	9660-9487
8521	D-1594	mussel shell	79.079	106.155	412.689	6	-	-	*	-2.6	-	8699	31	9750-9545
7942	D-1593	mussel shell	79.13	106.932	412.689	6	-	-	*	-1	-	8750	36	9895-9556
7597	D-1589	mussel shell	79.481	106.340	412.685	6	-	-	*	-4.7	-	8728	40	9890-9549
7941	D-1592	mussel shell	79.226	106.225	412.669	6	-	-	*	-7.4	-	8638	35	9681-9537
9024	D-1595	mussel shell	79.525	106.935	412.667	6	-	-	*	-7.8	-	8831	48	10,155-9691
11635	D-1891	mussel shell	79.617	105.182	412.666	6	-	-	*	-10.4	-	8030	37	9020-8724
8415	D-1583	mussel shell	82.97	104.720	412.657	6	-	-	*	0.6	-	8721	33	9888-9547
23362	D-8770	mussel shell	81.919	108.033	412.653	6	-	-	*	-2.9	-	8668	38	9709-9538
15126	D-8769	mussel shell	79.117	103.588	412.653	6	-	-	*	-2.6	-	8848	44	10,160-9720
8480	D-1585	mussel shell	82.668	104.822	412.642	6	-	-	*	-1.2	-	8706	33	9882-9545
16679	D-8786	mussel shell	82.743	108.088	412.630	6	-	-	*	-7.7	-	10,170	43	11,949-11,620
14887	D-8772	mussel shell	81.08	108.708	412.618	6	-	-	*	1.9	-	8387	41	9523-9292
15633	D-8781	mussel shell	81.281	109.809	412.612	6	-	-	*	-7.9	-	8826	46	10,153-9689
16470	D-8763	mussel shell	82.243	109.061	412.593	6	-	-	*	-5.4	-	9031	40	10,250-9969

24424	D-8760	mussel shell	82.245	109.284	412.587	6	-	-	*	1.8	-	9148	38	10,484-10,230
8489	D-1584	mussel shell	82.44	102.375	412.586	6	-	-	*	-6.6	-	8814	36	10,131-9686
10573	D-1599	mussel shell	82.206	101.910	412.580	6	-	-	*	-3.6	-	8680	38	9742-9541
8940	D-1582	mussel shell	82.568	104.416	412.578	6	-	-	*	-12	-	8676	38	9728-9540
17422	D-8775	mussel shell	82.579	109.211	412.564	6	-	-	*	2.6	-	8987	54	10,243-9910
15476	D-8773	mussel shell	79.531	103.292	412.561	6	-	-	*	-4.2	-	8706	38	9886-9544
17503	D-8776	mussel shell	82.572	109.067	412.550	6	-	-	*	-4.9	-	8652	35	9685-9540
7783	D-1597	mussel shell	79.448	102.813	412.540	6	-	-	*	-4.9	-	8711	33	9885-9546
18193	D-8780	mussel shell	81.334	109.922	412.531	6	-	-	*	-4.8	-	8017	43	9015-8653
26417	D-8784	mussel shell	81.768	108.376	412.526	6	-	-	*	-4.9	-	8807	43	10,149-9667
26420	D-8777	mussel shell	81.385	108.205	412.513	6	-	-	*	-6.6	-	8452	35	9534-9427
40093	D-8785	mussel shell	60.428	131.426	412.513	6	-	-	*	-2.6	-	8816	46	10,153-9679
28300	D-8778	mussel shell	79.437	106.935	412.478	6	-	-	*	-4	-	8546	40	9550-9475
28408	D-8779	mussel shell	80.309	107.521	412.457	6	-	-	*	-8.1	-	9287	41	10,641-10,295
52918	OxA-37,170	Bone	78.432	101.000	412.531	Lower LU6	6	3.2	45.2	-20.2	3.8	7984	40	8998-8650
29234	OxA-37,169	Bone	81.120	100.500	412.463	Mid LU5	4.16	3.4	42.6	-19.1	6.8	8141	38	9264-8998
59697	D-AMS 029851	Charcoal	79.842	106.657	411.981	Lower LU4	-	-	37	-20.8	-	7944	39	8986-8641
56440	OxA-38,048	Bone	79.922	106.325	412.295	Upper LU4	3.15	3.4	33	-19.6	7.6	9775	50	11,271-10,903
56817	OxA-X-2792-41	Bone	82.162	101.510	412.043	Lower LU4	1.4	3.4	19.3	-19.5	5.7	9110	50	10,481-10,191
56199	OxA-38,103	Bone	80.880	103.292	412.036	Lower LU4	3.4	3.2	41.3	-19.8	6.9	10,055	55	11,817-11,330
59391	OxA-X-2792-42	Bone	79.782	106.188	412.027	Lower LU4	2.1	3.3	21.7	-19.8	5.6	13,165	70	16,022-15,588
50554	OxA-37,171	Bone			412.027	Lower LU4	1.2	3.2	43.6	-20.1	6.9	10,005	40	11,700-11,275
56422	OxA-X-2792-43	Bone	80.900	103.327	411.990	Lower LU4	0.7	3.4	23.7	-20.5	6.6	10,050	60	11,817-11,320
57483	D-AMS 029850	Charcoal	82.795	107.393	412.009	Mid LU4	-	-	30	-21.3	-	9714	57	11,244-10,801
	TO-7349	Charcoal			411.900	Near surface of LU3	*	*	*	*	*	11,410	120	13,494-13,100
58223	OxA-X-2792-45	Bone	82.630	103.708	411.785	Upper LU3	1.1	3.5	11.7	-20.4	9.6	11,630	80	13,735-13,314
58628	OxA-38,104	Bone	82.185	100.293	411.695	Mid LU3	6.8	3.2	35.2	-20	6.9	7605	40	8518-8342
56446	D-AMS 029846	Charcoal	82.676	108.954	411.692	Mid LU3-Within F129 (hearth feature)	-	-	22	-34.3	-	12,348	71	14,845-14,095
59379	OxA-38,050	Charcoal	79.916	107.376	411.684	Mid LU3	-	-	29.1	-28.8	-	7831	40	8771-8477

56461	D-AMS 029749	Charcoal	82.945	108.417	411.667	Mid LU3- Within F129 (hearth feature)	-	-	21	-20.8	-	12,598	54	15,196-14,586
56623	D-AMS 029847	Charcoal	82.986	108.388	411.642	Mid LU3- Within F129 (hearth feature)	-	-	21	-21.2	-	12,472	61	14,996-14,305
56624	D-AMS 029848	Charcoal	80.020	108.459	411.641	Mid LU3 (on same surface as F129)	-	-	22	-19.9	-	12,363	49	14,841-14,148
53495	OxA-37,284	Bone				Lower LU3	5.16	3.5	30.8	-20.2	5.9	12,475	60	14,998-14,308
58720	OxA-38,051	Charcoal	78.267	102.672	411.572	Lower LU3	-	-			-	13,070	80	15,919-15,366
58398	OxA-X-2792-48	Bone	80.995	103.020	411.486	Lower LU3	1.6	3.3	28.3	-21	6.6	12,830	65	15,563-15,129
23283	OxA-38,106	Bone	82.520	102.629	411.310	Lower LU3	1.2	3.4	41.7	-20.9	4.5	7985	40	8998-8650
	Beta-114949	Charcoal			410.880	Within first pit (PFA2)	*	*	*	-22	*	11,370	40	13,316-13,169
56823	OxA-38,049	Bone	80.167	103.276	411.868	Within second pit (F134)	5.4	3.3	31.8	-20.2	5.5	10,010	50	11,735-11,275
58673	OxA-38,052	Bone	80.375	103.944	411.247	Within second pit (F134)	1.97	3.2	42	-19.3	7.8	13,335	75	16,276-15,796
59294	OxA-38,197	Bone	82.514	103.536	411.221	Within second pit (F134)	1.1	3.4	9.4	-20.2	10.4	11,720	80	13,779-13,443
59291	OxA-38,105	Bone	80.931	105.994	411.415	Within third pit (F135)	4.4	3.2	38.2	20	10.2	9955	50	11,688-11,243

*unreported

Table S5. Radiocarbon ages from living freshwater river mussels. pMC is the percentage of modern carbon in the combusted sample. Stable isotope ratios of C are expressed in per mil (‰) as measured by accelerator mass spectrometry on the reduced graphite target material.

**Laboratory no.**	**Sample #**	**Material**	**pMC**	**$\delta^{13}C$ (‰)**	**^{14}C Age**
D-AMS 002793	SHELL MFF-1	*Margaritifera falcata* shell carbonate	102.23	-26.9	MODERN
D-AMS 002625	SHELL MGA-1	*Gonidea angulata* shell carbonate	101.46	-7.4	MODERN
D-AMS 002626	SHELL MGA-2	*Gonidea angulata* shell carbonate	100.92	-10.7	MODERN
D-AMS 002628	SHELL MGA-4	*Gonidea angulata* shell carbonate	101.20	-10.8	MODERN
D-AMS 002629	SHELL MGA-5	*Gonidea angulata* shell carbonate	101.52	-5.2	MODERN
D-AMS 002744	SHELL MGA-6	*Gonidea angulata* shell carbonate	103.65	-22.6	MODERN
D-AMS 002745	SHELL MGA-7	*Gonidea angulata* shell carbonate	101.97	0.2	MODERN

Table S6. Luminescence properties under assumed water content of 10±5%.

Sample	Grain size (μm)	Aliquots	D_e (Gy)	D_e Overdispersion (%)	U (ppm)	Th (ppm)	K (%)	Ture W.C. (%)	Cosmic dose rate (Gy/ka)	Dose rate (Gy/ka)	Age (ka)
73-15-OSL-Lu2-1	90-125	14	38.44±1.12	7±1	2.15±0.09	11.90±0.33	2.39±0.07	4.44	0.11	3.52±0.15	10.92±0.56
73-15-OSL-Lu2-2	90-125	13	43.74±2.41	17±1	1.94±0.08	11.50±0.33	2.30±0.08	3.47	0.11	3.37±0.15	12.99±0.92
73-15-OSL-Lu2-3	90-125	15	39.79±1.51	12±1	2.08±0.09	12.00±0.34	2.43±0.07	4.52	0.11	3.55±0.16	11.21±0.66
73-15-OSL-Lu2-5	90-125	12	43.61±1.62	10±1	2.31±0.09	13.30±0.36	2.42±0.07	5.74	0.12	3.68±0.15	11.87±0.66
OSL-1	90-125	13	37.54±2.06	15±1	2.10±0.09	10.30±0.29	2.42±0.09	2.16	0.17	3.50±0.16	10.74±0.76
OSL-2	90-125	10	32.53±1.45	10±1	2.76±0.10	11.30±0.32	2.13±0.10	8.04	0.19	3.46±0.15	9.41±0.60

Table S7. Luminescence properties under assumed water content of 20±5%.

Sample	Grain size (μm)	Aliquots	D_e (Gy)	D_e Overdispersion (%)	U (ppm)	Th (ppm)	K (%)	Ture W.C. (%)	Cosmic dose rate (Gy/ka)	Dose rate (Gy/ka)	Age (ka)
73-15-OSL-Lu2-1	90-125	14	38.44±1.12	7±1	2.15±0.09	11.90±0.33	2.39±0.07	4.44	0.11	3.19±0.12	12.05±0.59
73-15-OSL-Lu2-2	90-125	13	43.74±2.41	17±1	1.94±0.08	11.50±0.33	2.30±0.08	3.47	0.11	3.05±0.12	14.33±0.99
73-15-OSL-Lu2-3	90-125	15	39.79±1.51	12±1	2.08±0.09	12.00±0.34	2.43±0.07	4.52	0.11	3.22±0.13	12.37±0.70
73-15-OSL-Lu2-5	90-125	12	43.61±1.62	10±1	2.31±0.09	13.30±0.36	2.42±0.07	5.74	0.12	3.33±0.13	13.10±0.70
OSL-1	90-125	13	37.54±2.06	15±1	2.10±0.09	10.30±0.29	2.42±0.09	2.16	0.17	3.17±0.13	11.83±0.82
OSL-2	90-125	10	32.53±1.45	10±1	2.76±0.10	11.30±0.32	2.13±0.10	8.04	0.19	3.14±0.13	10.36±0.64

Table S8. Luminescence properties under assumed water content of 30±5%.

Sample	Grain size (μm)	Aliquots	D_e (Gy)	D_e Overdispersion (%)	U (ppm)	Th (ppm)	K (%)	Ture W.C. (%)	Cosmic dose rate (Gy/ka)	Dose rate (Gy/ka)	Age (ka)
73-15-OSL-Lu2-1	90-125	14	38.44±1.12	7±1	2.15±0.09	11.90±0.33	2.39±0.07	4.44	0.11	2.92±0.11	13.17±0.62
73-15-OSL-Lu2-2	90-125	13	43.74±2.41	17±1	1.94±0.08	11.50±0.33	2.30±0.08	3.47	0.11	2.79±0.11	15.66±1.05
73-15-OSL-Lu2-3	90-125	15	39.79±1.51	12±1	2.08±0.09	12.00±0.34	2.43±0.07	4.52	0.11	2.94±0.12	13.52±0.74
73-15-OSL-Lu2-5	90-125	12	43.61±1.62	10±1	2.31±0.09	13.30±0.36	2.42±0.07	5.74	0.12	3.05±0.11	14.31±0.74
OSL-1	90-125	13	37.54±2.06	15±1	2.10±0.09	10.30±0.29	2.42±0.09	2.16	0.17	2.91±0.11	12.91±0.88
OSL-2	90-125	10	32.53±1.45	10±1	2.76±0.10	11.30±0.32	2.13±0.10	8.04	0.19	2.88±0.11	11.29±0.68

Table S9. Area B OSL age estimates using a 30% water content displayed stratigraphically.

Sample Number	Litho-Stratigraphic Unit	Median Age Estimate @ 30% Water Content	Age Range @ 2 Standard Deviations
OSL-2	LUB7	11.29±0.68 ka	9.93-12.65 ka
73-15-OSL-Lu2-5	LUB4	14.31 ±0.74 ka	12.83-15.79 ka
OSL-1	LUB3	12.91±0.88 ka	11.15-14.67 ka
73-15-OSL-Lu2-3	LUB3	13.52 ±0.74 ka	12.04-15.00 ka
73-15-OSL-Lu2-2	LUB2	15.66±1.05 ka	13.56-17.76 ka
73-15-OSL-Lu2-1	LUB2	13.17 ±0.62 ka	11.93-14.41 ka

};

Table S10. OxCal code for Bayesian analyses used in this study.

Cooper's Ferry Area A

```
Options()
{
 Resolution=50;
};
Plot()
{
 Outlier_Model("General",T(5),U(0,4),"t");
 Sequence("Cooper's Ferry Area A")
 {
  Boundary("Start LU-3");
  Phase("LU-3")
  {
   R_Date("OxA-38106", 7985, 40)
   {
    Outlier("General", 0.05);
   };
   R_Date("OxA-X-2792-48", 12830, 65)
   {
    Outlier("General", 0.05);
   };
   R_Date("OxA-38051", 13070, 80)
   {
    Outlier("General", 0.05);
   };
   R_Date("OxA-37284", 12475, 60)
   {
    Outlier("General", 0.05);
   };
```

```
R_Date("D-AMS 029848", 12363, 49)
{
 Outlier("General", 0.05);
};
Phase("F129")
{
 //F129
 R_Date("D-AMS 029847", 12472, 61)
 {
  Outlier("General", 0.05);
 };
 //F129
 R_Date("D-AMS 029749", 12598, 54)
 {
  Outlier("General", 0.05);
 };
 //F129
 R_Date("D-AMS 029846", 12348, 71)
 {
  Outlier("General", 0.05);
 };
 Date("F129");
};
R_Date("OxA-38050", 7831, 40)
{
 Outlier("General", 0.05);
};
Date("CFA017", N(2015-13710, 2620))
{
 Outlier("General", 0.05);
};
```

```
 R_Date("OxA-38104", 7605, 40)
 {
  Outlier("General", 0.05);
 };
 R_Date("OxA-X-2792-45", 11630, 80)
 {
  Outlier("General", 0.05);
 };
 R_Date("TO-7349", 11410, 120)
 {
  Outlier("General", 0.05);
 };
 Interval("Interval LU-3");
 Date("Date LU-3");
};
Boundary("End LU-3/Start hiatus");
Interval("Interval hiatus");
Boundary("End hiatus/Start LU-4");
Phase("LU-4")
{
 R_Date("D-AMS 029851", 7944, 39)
 {
  Outlier("General", 0.05);
 };
 R_Date("OxA-X-2792-43", 10050, 60)
 {
  Outlier("General", 0.05);
 };
 R_Date("D-AMS 029850", 9714, 57)
 {
  Outlier("General", 0.05);
```

```
 };
 R_Date("OxA-37171", 10005, 40)
 {
  Outlier("General", 0.05);
 };
 R_Date("OxA-X-2792-42", 13165, 70)
 {
  Outlier("General", 0.05);
 };
 Date("CFA018", N(2015-12730, 2400))
 {
  Outlier("General", 0.05);
 };
 R_Date("OxA-38103", 10055, 55)
 {
  Outlier("General", 0.05);
 };
 R_Date("OxA-X-2792-41", 9110, 50)
 {
  Outlier("General", 0.05);
 };
 R_Date("OxA-38048", 9775, 50)
 {
  Outlier("General", 0.05);
 };
 Date("Date LU-4");
 Interval("Interval LU-4");
};
Boundary("End LU-4/Start LU-5");
Phase("LU-5")
{
```

```
 };
 Date("CFA019", N(2015-12170, 2320))
 {
  Outlier("General", 0.05);
 };
 R_Date("OxA-37169", 8141, 38)
 {
  Outlier("General", 0.05);
 };
 Interval("Interval LU-5");
 Date("Date LU-5");
};
Boundary("End LU-5/Start LU6");
Phase("LU-6")
{
 R_Date("OxA-37170", 7984, 40)
 {
  Outlier("General", 0.05);
 };
 R_Date("D-8779", 9287, 41)
 {
  Outlier("General", 0.05);
 };
 R_Date("D-8778", 8546, 40)
 {
  Outlier("General", 0.05);
 };
 R_Date("D-8785", 8816, 46)
 {
  Outlier("General", 0.05);
 };
 R_Date("D-8777", 8452, 35)
```

```
{
 Outlier("General", 0.05);
};
R_Date("D-8784", 8807, 43)
{
 Outlier("General", 0.05);
};
R_Date("D-8780", 8017, 43)
{
 Outlier("General", 0.05);
};
R_Date("D-1597", 8711, 33)
{
 Outlier("General", 0.05);
};
R_Date("D-8776", 8652, 35)
{
 Outlier("General", 0.05);
};
R_Date("D-8773", 8706, 38)
{
 Outlier("General", 0.05);
};
R_Date("D-8775", 8987, 54)
{
 Outlier("General", 0.05);
};
R_Date("D-1582", 8676, 38)
{
 Outlier("General", 0.05);
};
```

```
R_Date("D-1599", 8680, 38)
{
 Outlier("General", 0.05);
};
R_Date("D-1584", 8814, 36)
{
 Outlier("General", 0.05);
};
R_Date("D-8760", 9148, 38)
{
 Outlier("General", 0.05);
};
R_Date("D-8763", 9031, 40)
{
 Outlier("General", 0.05);
};
R_Date("D-8781", 8826, 46)
{
 Outlier("General", 0.05);
};
R_Date("D-8772", 8387, 41)
{
 Outlier("General", 0.05);
};
R_Date("D-8786", 10170, 43)
{
 Outlier("General", 0.05);
};
R_Date("D-1585", 8706, 33)
{
 Outlier("General", 0.05);
```

```
};
R_Date("D-8769", 8848, 44)
{
 Outlier("General", 0.05);
};
R_Date("D-8770", 8668, 38)
{
 Outlier("General", 0.05);
};
R_Date("D-1583", 8721, 33)
{
 Outlier("General", 0.05);
};
R_Date("D-1891", 8030, 37)
{
 Outlier("General", 0.05);
};
R_Date("D-1595", 8831, 48)
{
 Outlier("General", 0.05);
};
R_Date("D-1592", 8638, 35)
{
 Outlier("General", 0.05);
};
R_Date("D-1589", 8728, 40)
{
 Outlier("General", 0.05);
};
R_Date("D-1593", 8750, 36)
{
```

```
};
 Outlier("General", 0.05);
};
R_Date("D-1594", 8699, 31)
{
 Outlier("General", 0.05);
};
R_Date("D-1590", 8588, 33)
{
 Outlier("General", 0.05);
};
R_Date("D-1591", 8610, 33)
{
 Outlier("General", 0.05);
};
R_Date("D-1586", 8728, 31)
{
 Outlier("General", 0.05);
};
R_Date("D-1588", 8678, 33)
{
 Outlier("General", 0.05);
};
R_Date("D-2073", 8692, 40)
{
 Outlier("General", 0.05);
};
R_Date("D-2076", 8792, 36)
{
 Outlier("General", 0.05);
};
R_Date("D-8762", 8802, 38)
```

```
{
 Outlier("General", 0.05);
};
R_Date("D-1587", 8766, 33)
{
 Outlier("General", 0.05);
};
R_Date("D-2074", 8845, 39)
{
 Outlier("General", 0.05);
};
R_Date("D-2075", 8838, 41)
{
 Outlier("General", 0.05);
};
R_Date("D-2081", 8807, 31)
{
 Outlier("General", 0.05);
};
R_Date("D-8787", 8735, 41)
{
 Outlier("General", 0.05);
};
R_Date("D-8774", 8886, 44)
{
 Outlier("General", 0.05);
};
R_Date("D-1598", 8778, 34)
{
 Outlier("General", 0.05);
};
```

```
R_Date("D-8761", 8882, 39)
{
 Outlier("General", 0.05);
};
R_Date("D-1581", 8799, 34)
{
 Outlier("General", 0.05);
};
R_Date("D-2069", 8683, 40)
{
 Outlier("General", 0.05);
};
R_Date("D-2078", 8725, 43)
{
 Outlier("General", 0.05);
};
R_Date("D-1596", 8783, 34)
{
 Outlier("General", 0.05);
};
R_Date("D-2077", 8802, 34)
{
 Outlier("General", 0.05);
};
R_Date("D-8771", 8764, 41)
{
 Outlier("General", 0.05);
};
R_Date("D-1869", 8619, 35)
{
 Outlier("General", 0.05);
```

```
};
R_Date("D-8782", 8833, 46)
{
 Outlier("General", 0.05);
};
R_Date("D-2079", 8626, 33)
{
 Outlier("General", 0.05);
};
R_Date("D-2080", 8831, 36)
{
 Outlier("General", 0.05);
};
R_Date("D-1870", 8804, 36)
{
 Outlier("General", 0.05);
};
R_Date("D-1871", 8759, 33)
{
 Outlier("General", 0.05);
};
R_Date("D-2068", 8833, 36)
{
 Outlier("General", 0.05);
};
R_Date("D-1602", 8596, 35)
{
 Outlier("General", 0.05);
};
R_Date("D-1606", 8600, 35)
{
```

```
};

 Outlier("General", 0.05);
};
R_Date("D-1607", 8704, 40)
{
 Outlier("General", 0.05);
};
R_Date("D-1601", 8725, 36)
{
 Outlier("General", 0.05);
};
R_Date("D-1608", 8884, 36)
{
 Outlier("General", 0.05);
};
R_Date("D-1889", 8802, 41)
{
 Outlier("General", 0.05);
};
R_Date("D-2620", 8687, 35)
{
 Outlier("General", 0.05);
};
R_Date("D-1872", 8624, 33)
{
 Outlier("General", 0.05);
};
R_Date("D-1868", 8685, 33)
{
 Outlier("General", 0.05);
};
R_Date("D-1867", 8624, 33)
```

```
{
 Outlier("General", 0.05);
};
R_Date("D-1604", 8740, 33)
{
 Outlier("General", 0.05);
};
R_Date("D-1603", 8884, 44)
{
 Outlier("General", 0.05);
};
R_Date("D-1894", 8783, 36)
{
 Outlier("General", 0.05);
};
R_Date("D-1895", 8680, 36)
{
 Outlier("General", 0.05);
};
R_Date("D-1893", 8666, 31)
{
 Outlier("General", 0.05);
};
R_Date("D-1605", 8848, 34)
{
 Outlier("General", 0.05);
};
R_Date("D-1600", 8792, 38)
{
 Outlier("General", 0.05);
};
```

```
R_Date("D-1873", 8676, 33)
{
 Outlier("General", 0.05);
};
R_Date("D-1838", 8635, 33)
{
 Outlier("General", 0.05);
};
R_Date("D-1882", 8783, 34)
{
 Outlier("General", 0.05);
};
R_Date("D-1884", 8716, 36)
{
 Outlier("General", 0.05);
};
R_Date("D-1892", 8721, 38)
{
 Outlier("General", 0.05);
};
R_Date("D-1887", 8935, 37)
{
 Outlier("General", 0.05);
};
R_Date("D-1885", 8874, 39)
{
 Outlier("General", 0.05);
};
R_Date("D-1881", 8671, 31)
{
 Outlier("General", 0.05);
```

```
};
R_Date("D-1876", 8687, 28)
{
 Outlier("General", 0.05);
};
R_Date("D-1877", 8624, 31)
{
 Outlier("General", 0.05);
};
R_Date("D-1878", 8563, 30)
{
 Outlier("General", 0.05);
};
R_Date("D-1886", 8831, 34)
{
 Outlier("General", 0.05);
};
R_Date("D-1879", 8577, 37)
{
 Outlier("General", 0.05);
};
R_Date("D-1890", 9138, 38)
{
 Outlier("General", 0.05);
};
R_Date("D-1880", 8814, 39)
{
 Outlier("General", 0.05);
};
R_Date("D-1874", 8635, 38)
{
```

```
   Outlier("General", 0.05);
  };
  R_Date("D-1888", 8617, 35)
  {
   Outlier("General", 0.05);
  };
  R_Date("D-1875", 8776, 38)
  {
   Outlier("General", 0.05);
  };
  R_Date("D-2072", 8773, 36)
  {
   Outlier("General", 0.05);
  };
  R_Date("D-2071", 8229, 36)
  {
   Outlier("General", 0.05);
  };
  R_Date("D-2070", 8749, 43)
  {
   Outlier("General", 0.05);
  };
  Interval("Interval LU-6");
  Date("Date LU-6");
 };
 Boundary("End LU6");
};
};
```

Cooper's Ferry Area B (Model A)

```
Plot()
```

```
{
 Outlier_Model("General",T(5),U(0,4),"t");
 Sequence("Cooper's Ferry Area B")
 {
  Boundary("Start LUB3");
  Phase("LUB3")
  {
   Phase("F151")
   {
    R_Date("OxA-41978", 13226, 52)
    {
     Outlier("General", 0.05);
    };
    R_Date("OxA-41974", 13091, 48)
    {
     Outlier("General", 0.05);
    };
    R_Date("OxA-X-3172-14", 13260, 240)
    {
     Outlier("General", 0.05);
    };
    Date("Date F151");
   };
   Phase("F78")
   {
    R_Date("OxA-41975", 13188, 48)
    {
     Outlier("General", 0.05);
    };
    R_Date("OxA-41976", 13175, 48)
    {
```

```
   Outlier("General", 0.05);
  };
  Date("Date F78");
 };
 Phase("F108")
 {
  R_Date("OxA-40389", 13147, 55)
  {
   Outlier("General", 0.05);
  };
  R_Date("OxA-40375", 13146, 59)
  {
   Outlier("General", 0.05);
  };
  Date("Date F108");
 };
 Date("Date LUB3");
 Interval("Interval LUB3");
};
Boundary("End LUB3/Start LUB4");
Interval("Interval hiatus 1");
Boundary("End LUB4/Start LUB5");
Phase("LUB5/F96")
{
 R_Date("OxA-40386", 9944, 39)
 {
  Outlier("General", 0.05);
 };
 R_Date("OxA-40353", 9885, 31)
 {
  Outlier("General", 0.05);
```

```
 };
 Date("Date LUB5/F96");
 Interval("Interval LUB5");
};
Boundary("End LUB5/Start hiatus");
Interval("Interval hiatus 2");
Boundary("End hiatus/Start LUB14");
Phase("LUB14")
{
 R_Date("UCIAMS-144543", 9620, 35)
 {
  Outlier("General", 0.05);
 };
 R_Date("OxA-41977", 9572, 33)
 {
  Outlier("General", 0.05);
 };
 R_Date("D-AMS 3581", 9244, 36)
 {
  Outlier("General", 0.05);
 };
 R_Date("D-AMS 3576", 9161, 35)
 {
  Outlier("General", 0.05);
 };
 R_Date("D-AMS 3572", 9133, 38)
 {
  Outlier("General", 0.05);
 };
 R_Date("D-AMS 3573", 9106, 32)
 {
```

```
  Outlier("General", 0.05);
 };
 R_Date("D-AMS 045621", 9658, 37)
 {
  Outlier("General", 0.05);
 };
 Phase("F111")
 {
  R_Date("OxA-40376 ", 9566, 34)
  {
   Outlier("General", 0.05);
  };
  R_Date("OxA-40387", 9505, 38)
  {
   Outlier("General", 0.05);
  };
  Date("Date F111");
 };
 Date("Date LUB14");
 Interval("Interval LUB14");
};
Boundary("End LUB14/Start LUB15");
Interval("Interval hiatus 3");
Boundary("End LUB15/Start LUB16");
Label("LUB16");
R_Date("D-AMS 045619", 1517, 20)
{
 Outlier("General", 0.05);
};
Boundary("End LUB16");
};
```

```
};
```

Cooper’s Ferry Area B (Model B)

```
Plot()
{
 Outlier_Model("General",T(5),U(0,4),"t");
 Sequence("Cooper's Ferry Area B")
 {
  Boundary("Start LUB2");
  Phase("LUB2")
  {
   Date("73-15-OSL-Lu2-1", N(2012-10920, 560))
   {
    Outlier("General", 0.05);
   };
   Date("73-15-OSL-Lu2-2", N(2012-12990, 920))
   {
    Outlier("General", 0.05);
   };
  };
  Boundary("End LUB2/Start LUB3");
  Phase("LUB3")
  {
   Phase("F151")
   {
    R_Date("OxA-41978", 13226, 52)
    {
     Outlier("General", 0.05);
    };
    R_Date("OxA-41974", 13091, 48)
    {
     Outlier("General", 0.05);
    };
    R_Date("OxA-X-3172-14", 13260, 240)
    {
     Outlier("General", 0.05);
    };
    Date("Date F151");
   };
   Phase("F78")
   {
    R_Date("OxA-41975", 13188, 48)
    {
     Outlier("General", 0.05);
    };
    R_Date("OxA-41976", 13175, 48)
    {
     Outlier("General", 0.05);
    };
    Date("Date F78");
   };
   Phase("F108")
   {
    R_Date("OxA-40389", 13147, 55)
    {
     Outlier("General", 0.05);
    };
    R_Date("OxA-40375", 13146, 59)
    {
     Outlier("General", 0.05);
    };
    Date("Date F108");
   };
   Date("73-15-OSL-Lu2-3", N(2012-11210, 660))
   {
```

```
  Outlier("General", 0.05);
 };
 Date("OSL-1", N(2012-10740, 760))
 {
  Outlier("General", 0.05);
 };
 Date("Date LUB3");
 Interval("Interval LUB3");
};
Boundary("End LUB3/Start LUB4");
Date("73-15-OSL-Lu2-5", N(2012-11870, 660))
{
 Outlier("General", 0.05);
};
Boundary("End LUB4/Start LUB5");
Phase("LUB5/F96")
{
 R_Date("OxA-40386", 9944, 39)
 {
  Outlier("General", 0.05);
 };
 R_Date("OxA-40353", 9885, 31)
 {
  Outlier("General", 0.05);
 };
 Date("Date LUB5/F96");
 Interval("Interval LUB5");
};
Boundary("End LUB5/Start LUB6-12");
Date("OSL-2", N(2012-9410, 600))
{
 Outlier("General", 0.05);
};
Boundary("End LUB6-12/Start LUB14");
Phase("LUB14")
{
 R_Date("UCIAMS-144543", 9620, 35)
 {
  Outlier("General", 0.05);
 };
 R_Date("OxA-41977", 9572, 33)
 {
  Outlier("General", 0.05);
 };
 R_Date("D-AMS 3581", 9244, 36)
 {
  Outlier("General", 0.05);
 };
 R_Date("D-AMS 3576", 9161, 35)
 {
  Outlier("General", 0.05);
 };
 R_Date("D-AMS 3572", 9133, 38)
 {
  Outlier("General", 0.05);
 };
 R_Date("D-AMS 3573", 9106, 32)
 {
  Outlier("General", 0.05);
 };
 R_Date("D-AMS 045621", 9658, 37)
 {
  Outlier("General", 0.05);
 };
 Phase("F111")
 {
  R_Date("OxA-40376 ", 9566, 34)
  {
   Outlier("General", 0.05);
  };
```

```
   R_Date("OxA-40387", 9505, 38)
   {
    Outlier("General", 0.05);
   };
   Date("Date F111");
  };
  Date("Date LUB14");
  Interval("Interval LUB14");
 };
 Boundary("End LUB14/Start LUB15");
 Interval("Interval hiatus 3");
 Boundary("End LUB15/Start LUB16");
 Label("LUB16");
 R_Date("D-AMS 045619", 1517, 20)
 {
  Outlier("General", 0.05);
 };
 Boundary("End LUB16");
};
};
```

Cooper's Ferry Area B (Model C)

```
Plot()

{

 Outlier_Model("General",T(5),U(0,4),"t");

 Sequence("Cooper's Ferry Area B")

 {

  Boundary("Start LUB2");

  Phase("LUB2")

  {

   Date("73-15-OSL-Lu2-1", N(2012-12050, 590))

   {

    Outlier("General", 0.05);

   };

   Date("73-15-OSL-Lu2-2", N(2012-14330, 990))

   {

    Outlier("General", 0.05);

   };

  };

  Boundary("End LUB2/Start LUB3");

  Phase("LUB3")

  {
```

```
Phase("F151")
{
 R_Date("OxA-41978", 13226, 52)
 {
  Outlier("General", 0.05);
 };
 R_Date("OxA-41974", 13091, 48)
 {
  Outlier("General", 0.05);
 };
 R_Date("OxA-X-3172-14", 13260, 240)
 {
  Outlier("General", 0.05);
 };
 Date("Date F151");
};
Phase("F78")
{
 R_Date("OxA-41975", 13188, 48)
 {
  Outlier("General", 0.05);
 };
 R_Date("OxA-41976", 13175, 48)
 {
  Outlier("General", 0.05);
 };
 Date("Date F78");
};
Phase("F108")
{
 R_Date("OxA-40389", 13147, 55)
```

```
   {
    Outlier("General", 0.05);
   };
   R_Date("OxA-40375", 13146, 59)
   {
    Outlier("General", 0.05);
   };
   Date("Date F108");
  };
  Date("73-15-OSL-Lu2-3", N(2012-12370, 700))
  {
   Outlier("General", 0.05);
  };
  Date("OSL-1", N(2012-11830, 820))
  {
   Outlier("General", 0.05);
  };
  Date("Date LUB3");
  Interval("Interval LUB3");
 };
 Boundary("End LUB3/Start LUB4");
 Date("73-15-OSL-Lu2-5", N(2012-13100, 700))
 {
  Outlier("General", 0.05);
 };
 Boundary("End LUB4/Start LUB5");
 Phase("LUB5/F96")
 {
  R_Date("OxA-40386", 9944, 39)
  {
   Outlier("General", 0.05);
```

```
 };
 R_Date("OxA-40353", 9885, 31)
 {
  Outlier("General", 0.05);
 };
 Date("Date LUB5/F96");
 Interval("Interval LUB5");
};
Boundary("End LUB5/Start LUB6-12");
Date("OSL-2", N(2012-10360, 640))
{
 Outlier("General", 0.05);
};
Boundary("End LUB6-12/Start LUB14");
Phase("LUB14")
{
 R_Date("UCIAMS-144543", 9620, 35)
 {
  Outlier("General", 0.05);
 };
 R_Date("OxA-41977", 9572, 33)
 {
  Outlier("General", 0.05);
 };
 R_Date("D-AMS 3581", 9244, 36)
 {
  Outlier("General", 0.05);
 };
 R_Date("D-AMS 3576", 9161, 35)
 {
  Outlier("General", 0.05);
```

```
 };
 R_Date("D-AMS 3572", 9133, 38)
 {
  Outlier("General", 0.05);
 };
 R_Date("D-AMS 3573", 9106, 32)
 {
  Outlier("General", 0.05);
 };
 R_Date("D-AMS 045621", 9658, 37)
 {
  Outlier("General", 0.05);
 };
 Phase("F111")
 {
  R_Date("OxA-40376 ", 9566, 34)
  {
   Outlier("General", 0.05);
  };
  R_Date("OxA-40387", 9505, 38)
  {
   Outlier("General", 0.05);
  };
  Date("Date F111");
 };
 Date("Date LUB14");
 Interval("Interval LUB14");
};
Boundary("End LUB14/Start LUB15");
Interval("Interval hiatus 3");
Boundary("End LUB15/Start LUB16");
```

```
  Label("LUB16");
  R_Date("D-AMS 045619", 1517, 20)
  {
   Outlier("General", 0.05);
  };
  Boundary("End LUB16");
 };
};
```

Cooper’s Ferry Area B (Model D)

```
Plot()
{
 Outlier_Model("General",T(5),U(0,4),"t");
 Sequence("Cooper's Ferry Area B")
 {
  Boundary("Start LUB2");
  Phase("LUB2")
  {
   Date("73-15-OSL-Lu2-1", N(2012-13170, 620))
   {
    Outlier("General", 0.05);
   };
   Date("73-15-OSL-Lu2-2", N(2012-15660, 1050))
   {
    Outlier("General", 0.05);
   };
  };
  Boundary("End LUB2/Start LUB3");
  Phase("LUB3")
```

```
{
 Phase("F151")
 {
  R_Date("OxA-41978", 13226, 52)
  {
   Outlier("General", 0.05);
  };
  R_Date("OxA-41974", 13091, 48)
  {
   Outlier("General", 0.05);
  };
  R_Date("OxA-X-3172-14", 13260, 240)
  {
   Outlier("General", 0.05);
  };
  Date("Date F151");
 };
 Phase("F78")
 {
  R_Date("OxA-41975", 13188, 48)
  {
   Outlier("General", 0.05);
  };
  R_Date("OxA-41976", 13175, 48)
  {
   Outlier("General", 0.05);
  };
  Date("Date F78");
 };
 Phase("F108")
 {
```

```
  R_Date("OxA-40389", 13147, 55)
  {
   Outlier("General", 0.05);
  };
  R_Date("OxA-40375", 13146, 59)
  {
   Outlier("General", 0.05);
  };
  Date("Date F108");
 };
 Date("73-15-OSL-Lu2-3", N(2012-13520, 740))
 {
  Outlier("General", 0.05);
 };
 Date("OSL-1", N(2012-12910, 880))
 {
  Outlier("General", 0.05);
 };
 Date("Date LUB3");
 Interval("Interval LUB3");
};
Boundary("End LUB3/Start LUB4");
Date("73-15-OSL-Lu2-5", N(2012-14310, 740))
{
 Outlier("General", 0.05);
};
Boundary("End LUB4/Start LUB5");
Phase("LUB5/F96")
{
 R_Date("OxA-40386", 9944, 39)
 {
```

```
  Outlier("General", 0.05);
 };
 R_Date("OxA-40353", 9885, 31)
 {
  Outlier("General", 0.05);
 };
 Date("Date LUB5/F96");
 Interval("Interval LUB5");
};
Boundary("End LUB5/Start LUB6-12");
Date("OSL-2", N(2012-11290, 680))
{
 Outlier("General", 0.05);
};
Boundary("End LUB6-12/Start LUB14");
Phase("LUB14")
{
 R_Date("UCIAMS-144543", 9620, 35)
 {
  Outlier("General", 0.05);
 };
 R_Date("OxA-41977", 9572, 33)
 {
  Outlier("General", 0.05);
 };
 R_Date("D-AMS 3581", 9244, 36)
 {
  Outlier("General", 0.05);
 };
 R_Date("D-AMS 3576", 9161, 35)
 {
```

```
  Outlier("General", 0.05);
 };
 R_Date("D-AMS 3572", 9133, 38)
 {
  Outlier("General", 0.05);
 };
 R_Date("D-AMS 3573", 9106, 32)
 {
  Outlier("General", 0.05);
 };
 R_Date("D-AMS 045621", 9658, 37)
 {
  Outlier("General", 0.05);
 };
 Phase("F111")
 {
  R_Date("OxA-40376 ", 9566, 34)
  {
   Outlier("General", 0.05);
  };
  R_Date("OxA-40387", 9505, 38)
  {
   Outlier("General", 0.05);
  };
  Date("Date F111");
 };
 Date("Date LUB14");
 Interval("Interval LUB14");
};
Boundary("End LUB14/Start LUB15");
Interval("Interval hiatus 3");
```

```
  Boundary("End LUB15/Start LUB16");
  Label("LUB16");
  R_Date("D-AMS 045619", 1517, 20)
  {
   Outlier("General", 0.05);
  };
  Boundary("End LUB16");
 };
};
```

REFERENCES AND NOTES

1. L. G. Davis, D. B. Madsen, L. Becerra-Valdivia, T. Higham, D. A. Sisson, S. M. Skinner, D. Stueber, A. J. Nyers, A. Keen-Zebert, C. Neudorf, M. Cheyney, M. Izuho, F. Iizuka, S. R. Burns, C. W. Epps, S. C. Willis, I. Buvit, Late upper paleolithic occupation at Cooper's Ferry, Idaho, USA, ~16,000 years ago. *Science* **365**:891–897 (2019).

2. JD Lancaster "Progress in the search for early sites in the lower Salmon River canyon, Idaho: A case study in geoarchaeological prospection," thesis, Oregon State University, Corvallis (2015).

3. L. G. Davis, K. Muehlenbachs, C. E. Schweger, N. W. Rutter, Differential response of vegetation to postglacial climate in the lower Salmon River canyon, Idaho. *Palaeogeogr. Palaeoclimatol. Palaeoecol.* **185**, 339–354 (2002).

4. L. G. Davis "The coevolution of early hunter-gatherer culture and riparian ecosystems in the southern Columbia River Plateau," thesis, University of Alberta, Edmonton (2001).

5. S. J. Fiedel, B. A. Potter, J. E. Morrow, M. K. Faught, C. Vance Haynes Jr, J. C. Chatters, *PaleoAm.* **7**, 28–42 (2020).

6. T. A. Surovell, S. A. Allaun, B. A. Crass, J. A. M. Gingerich, K. E. Graf, C. E. Holmes, R. L. Kelly, M. Kornfeld, K. E. Krasinski, M. L. Larson, S. R. Pelton, B. T. Wygal, Late date of human arrival to North America: Continental scale differences in stratigraphic integrity of pre-13,000 BP archaeological sites. *PLOS ONE* **17**, e0264092 (2022).

7. M. R. Waters, T. W. Stafford Jr., D. L. Carlson, The age of Clovis—13,050 to 12,750 cal yr B.P. *Sci. Adv.* **6**, eaaz0455 (2020).

8. L. Becerra-Valdivia, T. Higham, The timing and effect of the earliest human arrivals in North America. *Nature* **584**, 93–97 (2020).

9. Hokkaido Archaeological Operation Center, "Shirataki Isekigun VII: The Shirataki Group of Sites VII" (in Japanese) (Hokkaido Archaeological Operation Center, 2006).

10. B. R. Butler, The earlier cultural remains at Cooper's Ferry, *Tebiwa* **12**, 35–50 (1969).

11. See the Supplementary Materials.

12. L. G. Davis, A. J. Nyers, S. C. Willis, Context, provenance and technology of a western stemmed tradition artifact cache from the Cooper's Ferry Site, Idaho. *Am. Antiq.* **79**, 596–615 (2014).

13. A. J. Carroll "Perspectives on pits of the western stemmed tradition: An analysis on the contents of Feature 59 at the Cooper's Ferry site," thesis, Oregon State University, Corvallis (2018).

14. T. J. Williams, M. B. Collins, K. Rodrigues, W. J. Rink, N. Velchoff, A. Keen-Zebert, A. Gilmer, C. D. Frederick, S. J. Ayala, E. R. Prewitt, Evidence of an early projectile point technology in North America at the Gault Site, Texas, USA. *Sci. Adv.* **4**, eaar5954 (2018).

15. M. R. Waters, J. L. Keene, S. L. Forman, E. R. Prewitt, D. L. Carlson, J. E. Wiederhold, Pre-Clovis projectile points at the Debra L. Friedkin site, Texas—Implications for the Late Pleistocene peopling of the Americas. *Sci. Adv.* **4**, eaat4505 (2018).

16. L. A. A. de Anda, M. Maldonada-Koerdell, Association of artifacts with mammoth in the Valley of Mexico. *Am. Antiq.* **4**, 332–340 (1953).

17. L. A. A. de Anda, The second mammoth and associated artifacts at Santa Isabel Iztapan, Mexico. *Am. Antiq.* **22**, 12–28 (1956).

18. J. V. Moreno-Mayar, L. Vinner, Peter de Barros Damgaard, Constanza de la Fuente, J. Chan, J. P. Spence, M. E. Allentoft, T. Vimala, F. Racimo, T. Pinotti, S. Rasmussen, A. Margaryan, M. I. Orbegozo, D. Mylopotamitaki, M. Wooller, C. Bataille, L. Becerra-Valdivia, D. Chivall, D. Comeskey, T. Devièse, D. K. Grayson, L. George, H. Harry, V. Alexandersen, C. Primeau, J. Erlandson, C. Rodrigues-Carvalho, S. Reis, M. Q. R. Bastos, J. Cybulski, C. Vullo, F. Morello, M. Vilar, S. Wells, K. Gregersen, K. L. Hansen, N. Lynnerup, M. M. Lahr, K. Kjær, A. Strauss, M. Alfonso-Durruty, A. Salas, H. Schroeder, T. Higham, R. S. Malhi, J. T. Rasic, L. Souza, F. R. Santos, A.-S. Malaspinas, M. Sikora, R. Nielsen, Y. S. Song, D. J. Meltzer, E. Willerslev, Early human dispersals within the Americas. *Science* **362**, eaav2621 (2018).

19. M. Raghavan, M. Steinrücken, K. Harris, S. Schiffels, S. Rasmussen, M. De Giorgio, A.

Albrechtsen, C. Valdiosera, M. C. Ávila-Arcos, A.-S. Malaspinas, A. Eriksson, I. Moltke, M. Metspalu, J. R. Homburger, J. Wall, O. E. Cornejo, J. Víctor Moreno-Mayar, T. S. Korneliussen, T. Pierre, M. Rasmussen, P. F. Campos, P. de Barros Damgaard, M. E. Allentoft, J. Lindo, E. Metspalu, R. Rodríguez-Varela, J. Mansilla, C. Henrickson, A. Seguin-Orlando, H. Malmström, T. Stafford Jr, S. S. Shringarpure, A. Moreno-Estrada, M. Karmin, K. Tambets, A. Bergström, Y. Xue, V. Warmuth, A. D. Friend, J. Singarayer, P. Valdes, F. Balloux, I. Leboreiro, J. L. Vera, H. Rangel-Villalobos, D. Pettener, D. Luiselli, L. G. Davis, E. Heyer, C. P. E. Zollikofer, M. S. Ponce de León, C. I. Smith, V. Grimes, K.-A. Pike, M. Deal, B. T. Fuller, B. Arriaza, V. Standen, M. F. Luz, F. Ricaut, N. Guidon, L. Osipova, M. I. Voevoda, O. L. Posukh, O. Balanovsky, M. Lavryashina, Y. Bogunov, E. Khusnutdinova, M. Gubina, E. Balanovska, S. Fedorova, S. Litvinov, B. Malyarchuk, M. Derenko, M. J. Mosher, D. Archer, J. Cybulski, B. Petzelt, J. Mitchell, R. Worl, P. J. Norman, P. Parham, B. M. Kemp, T. Kivisild, C. Tyler-Smith, M. S. Sandhu, M. Crawford, R. Villems, D. G. Smith, M. R. Waters, T. Goebel, J. R. Johnson, R. S. Malhi, M. Jakobsson, D. J. Meltzer, A. Manica, R. Durbin, C. D. Bustamante, Y. S. Song, R. Nielsen, E. Willerslev, Genomic evidence for the Pleistocene and recent population history of Native Americans. *Science* **349**, aab3884 (2015).

20. B. Llamas, L. Fehren-Schmitz, G. Valverde, J. Soubrier, S. Mallick, N. Rohland, S. Nordenfelt, C. Valdiosera, S. M. Richards, A. Rohrlach, Maria Inés Barreto Romero, I. F. Espinoza, E. T. Cagigao, L. W. Jiménez, K. Makowski, Ilán Santiago Leboreiro Reyna, J. M. Lory, Julio Alejandro Ballivián Torrez, M. A. Rivera, R. L. Burger, M. C. Ceruti, J. Reinhard, R. Spencer Wells, G. Politis, C. M. Santoro, V. G. Standen, C. Smith, D. Reich, S. Y. W. Ho, A. Cooper, W. Haak, Ancient mitochondrial DNA provides high-resolution time scale of the peopling of the Americas. *Sci. Adv.* **2**, e1501385 (2016).

21. T. Pinotti, A. Bergström, M. Geppert, M. Bawn, D. Ohasi, W. Shi, D. R. Lacerda, A. Solli, J. Norstedt, K. Reed, K. Dawtry, F. González-Andrade, Cesar Paz-Y-Miño, S. Revollo, C. Cuellar, M. S. Jota, J. E. Santos Jr, Q. Ayub, T. Kivisild, J. R. Sandoval, R. Fujita, Y. Xue, L. Roewer, F. R. Santos, C. Tyler-Smith, Y chromosome sequences reveal a short Beringian Standstill, rapid expansion, and early population structure of native american founders. *Curr. Biol.* **29**, 149–157.e3 (2019).

22. K. Morisaki, M. Izuho, K. Terry, H. Sato, Lithics and climate: Technological responses to landscape change in Upper Palaeolithic northern Japan. *Antiquity* **89**, 554–572 (2015).

23. K. Morisaki, N. Oda, D. Kunikita, Y. Sasaki, Y. Kuronuma, A. Iwase, T. Yamazaki, N. Ichida, H. Sato, Sedentism, pottery and inland fishing in late Glacial Japan: A reassessment of the Maedakochi site. *Antiquity* **93**, 1442–1459 (2019).

24. I. Y. Ponkratova, L. G. Davis, D. W. Bean, D. B. Madsen, A. J. Nyers, I. Buvit, Technological similarities between ~13 ka stemmed points from Ushki V, Kamchatka, Russia, and the earliest stemmed points in North America, in *Maritime Prehistory of Northeast Asia*, J. Cassidy, I. Ponkratova, B. Fitzhugh, Eds. (Springer Nature, 2022), pp. 233–262.

25. F. Iizuka, The timing and behavioral context of the Late-Pleistocene adoption of ceramics in greater East and Northeast Asia and the First People (without pottery) in the Americas. *PaleoAm.* **4**, 267–324. (2018).

26. D. Natsuki, Migration and adaptation of Jomon people during Pleistocene/Holocene transition period in Hokkaido, Japan, *Quat. Int.* **608-609**, 49–64 (2022).

27. G. R. Scott, D. H. O'Rourke, J. A. Raff, J. C. Tackney, L. J. Hlusko, S. A. Elias, L. Bourgeon, O. Potapova, E. Pavlova, V. Pitulko, J. F. Hoffecker, Peopling the Americas: Not "out of Japan". *PaleoAm.* **7**, 309–332 (2021).

28. L. G. Davis, D. B. Madsen, The coastal migration theory: Formulation and testable hypotheses. *Quat. Sci. Rev.* **249**, 106605 (2020).

29. I. Buvit, K. Terry, and M. Izuho, Pathways along the Pacific: Using early stone tools to reconstruct coastal migration between Japan and the Americas, in *Paleolandscapes and Archaeology*, M. T. Carson Ed. (Routledge, 2021), pp. 39–81.

30. C. B. Ramsey, Development of the radiocarbon calibration program. *Radiocarbon* **43**, 355–363 (2001).

31. P. J. Reimer, W. E. N. Austin, E. Bard, A. Bayliss, P. G. Blackwell, C. Bronk Ramsey, M.

Butzin, H. Cheng, R. L. Edwards, M. Friedrich, P. M. Grootes, T. P. Guilderson, I. Hajdas, T. J. Heaton, A. G. Hogg, K. A. Hughen, B. Kromer, S. W. Manning, R. Muscheler, J. G. Palmer, C. Pearson, J. van der Plicht, R. W. Reimer, D. A Richards, E. M. Scott, J. R. Southon, C. S. M. Turney, L. Wacker, F. Adolphi, U. Büntgen, M. Capano, S. M. Fahrni, A. Fogtmann-Schulz, R. Friedrich, P. Köhler, S. Kudsk, F. Miyake, J. Olsen, F. Reinig, M. Sakamoto, A. Sookdeo, S. Talamo, The IntCal20 Northern Hemisphere radiocarbon age calibration curve (0-55 cal kBP). *Radiocarbon* **62**, 725–757 (2020).

32. A. S. Dyke, An outline of North American deglaciation with emphasis on central and northern Canada, in *Quaternary Glaciations—Extent and Chronology. Part II: North America*, J. Ehlers, P. L. Gibbard, Eds. (Elsevier, 2004), pp. 373–424.

33. B. J. Quirk, E. Huss, B. J. C. Laabs, E. Leonard, J. Licciardi, M.A. Plummer, Late Pleistocene glacial chronologies and paleoclimate in the northern Rocky Mountains *Clim. Past* **18**, 293–312 (2022).

34. R. B. Waitt Jr., Case for periodic, colossal jökulhlaups from Pleistocene glacial Lake Missoula. *Geol. Soc. Am. Bull.* **96**, 1271–1286 (1985).

35. R. B. Morrison, Quaternary stratigraphic, hydrologic, and climatic history of the Great Basin, with emphasis on Lakes Lahontan, Bonneville, and Tecopa, in *Quaternary Nonglacial Geology: Conterminous U.S., The Geology of North America*, vol. K-2, R. B. Morrison, Ed. (Geological Society of America, 1991), pp. 283–320.

36. J. Clark, J. X. Mitrovica, K. Latychev, Glacial isostatic adjustment in central Cascadia: Insights from three-dimensional Earth modeling. *Geology* **47**, 295–298 (2019).

37. Google Earth v.7.3.4.8642 (July 30, 2016) lower Salmon River canyon, western Idaho, USA. 445.905576° N, −116.596311° W, eye alt 627 m. Landsat 2019; www.earth.google.com [accessed 16 June 2022].

38. Schwede (*39*) identified this locale as "Nipéheme." However, Nakia Williamson-Cloud, Nez Perce Tribe Cultural Resource Program Director, explained that the proper reference to this

ancient village location is “Nipéhe.”

39. M. L. Schwede, “An ecological study of Nez Perce settlement patterns,” thesis, Washington State University, Pullman, WA (1966).

40. L. G. Davis, D. A. Sisson, An early stemmed point cache from the lower Salmon River canyon of West-Central Idaho. *Curr. Res. Pleistocene* **15**, 12–14 (1998).

41. L. G. Davis, C. E. Schweger, Geoarchaeological context of late pleistocene and early holocene occupation at the Cooper’s Ferry Site, Western Idaho, USA. *Geoarchaeology* **19**, 685–704 (2004).

42. L. G. Davis, A. J. Nyers, S. C. Willis, Context, provenance and technology of a Western Stemmed Tradition artifact cache from the Cooper’s Ferry Site, Idaho, *Am. Antiq.* **79**, 596–615 (2014).

43. L. G. Davis, D. W. Bean, A. J. Nyers, Orphometric and technological attributes of western stemmed tradition projectile points revealed in a second artifact cache from the Cooper's Ferry Site, Idaho. *Am. Antiq.* **82**, 536–557 (2017).

44. P. W. Birkeland, *Soils and Geomorphology* (Oxford Univ. Press, 1984).

45. Soil Survey Division Staff, *Soil Survey Manual* (USDA handbook no. 18, U.S. Printing Office, 1993).

46. North American Commission on Stratigraphic Nomenclature (NACOSN), North American stratigraphic code. *AAPG. Bull.* **67**, 841–875 (1983).

47. K. Pye, The nature, origin and accumulation of loess. *Quat. Sci. Rev.* **14**, 653–667 (1995).

48. K. Zamanian, K. Pustovoytov, Y. Kuzyakov, Pedogenic carbonates: Forms and formation processes. *Earth Sci. Rev.* **157**, 1–17 (2016).

49. L. L. Foley, “Quaternary chronology of the Palouse loess near Washtucna, Eastern Washington,” thesis, Western Washington University, Bellingham, WA (1982).

50. E. V. McDonald, A. J. Busacca, Late Quaternary stratigraphy of loess in the channeled scabland and Palouse regions of Washington State. *Quatern. Res.* **38**,141–156 (1992).

51. M. Sponheimer, C. M. Ryder, H. Fewlass, E. K. Smith, W. J. Pestle, S. Talamo, Saving old bones: A non-destructive method for bone collagen prescreening. *Sci. Rep.* **9**, 13928 (2019).

52. J. Workman, Jr., L. Weyer, *Practical Guide to Interpretative Near-Infrared Spectroscopy* (CRC Press, 2012).

53. F. Faris, M. Thorniley, Y. Wickramasinghe, R. Houston, P. Rolfe, N. Livera, A. Spencer, Non-invasive in vivo near-infrared optical measurement of the penetration depth in the neonatal head. *Clin. Phys. Physiol. Meas.* **12**, 353–358 (1991).

54. I. O. Afara C. Florea, I. A. Olumegbon, C. T. Eneh, M. K. H. Malo, R. K. Korhonen, J. Töyräs, Characterizing human subchondral bone properties using near-infrared (NIR) spectroscopy. *Sci. Rep.* **8**, 9733 (2018).

55. G. H. Turner-Walker, "The characterization of fossil bone," thesis, Durham University (1993).

56. G. H. Turner-Walker, M. Jans, Reconstructing taphonomic histories using histological analysis. *Palaeogeogr. Palaeoclimatol. Palaeoecol.* **266**, 227–235 (2008).

57. C. A. Suarez, M. J. Kohn, Caught in the act: A case study on microscopic scale physicochemical effects of fossilization on stable isotopic composition of bone. *Geochim. Cosmochim. Acta* **268**, 277–295 (2020).

58. C. B. Ramsey, Bayesian analysis of radiocarbon dates. *Radiocarbon* **51**, 337–360 (2009).

59. C. B. Ramsey, Dealing with outliers and offsets in radiocarbon dating. *Radiocarbon* **51**, 1023–1045 (2009).

60. A. S. Murray, A. G. Wintle, The single aliquot regenerative dose protocol: Potential for improvements in reliability. *Radiat. Meas.* **37**, 377–381 (2003).

61. G. Duller, Distinguishing quartz and feldspar in single grain luminescence measurements.

Radiat. Meas. **37**, 161–165 (2003).

62. P. Liang, S. L. Forman, LDAC: An Excel-based program for luminescence equivalent dose and burial age calculations. *Ancient TL* **37**, 21–40 (2019).

63. G. Li, F. Chen, D. Xia, H. Yang, X. Zhang, D. Madsen, C. Oldknow, H. Wei, Z. Rao, M. Qiang, A Tianshan Mountains loess-paleosol sequence indicates anti-phase climatic variations in arid Center Asia and in East Asia. *Earth Planet. Sci. Lett.* **494**, 153–163 (2018).

64. L. G. Davis, Paleoseismicity and prehistoric anadromous fish exploitation in the salmon river basin, Western Idaho. *N. Amer. Arch.* **23**, 233–263 (2008).

65. E. J. Rhodes, Optically stimulated luminescence dating of sediments over the past 200,000 years. *Annu. Rev. Earth Planet. Sci.* **39**, 461–488 (2011).

66. L. G. Davis, D. W. Bean, A. J. Nyers, D. R. Brauner, GLiMR: A GIS-based method for the geometric morphometric analysis of Artifacts. *Lithic Technol.* **40**, 199–217 (2015).

67. A. Steffen, E. J. Skinner, P. W. Ainsworth, A view to the core: Technological units and debitage analysis, in *Unit Issues in Archaeology: Measuring Time, Space, and Material*, A. F. Ramenofsky, A. Steffen, Eds. (University of Utah Press, 1997), pp. 131–146.

68. T. L. Ozbun, D. O. Stueber, M. Zehendner, J. L. Fagan, Lithic debitage and formed tools, in *Marmes Rockshelter: A Final Report on 11,000 Years of Cultural Use*, B. A. Hicks, Ed. (Washington State University Dent. Press, 2004), pp. 159–227.

69. F. Sellet, Chaine operatoire: The concept and its applications. *Lithic Technol.* **18**, 106–112 (1993).

70. N. Toth, K. Schick, in *The Cutting Edge: New Approaches to the Archaeology of Human Origins*, K. Schick, N. Toth, Eds. (Stone Age Institute, 2009), pp. 267–344.

71. L. G. Davis, S. C. Willis, in *Convergent Evolution and Stone-Tool Technology*, M. O'Brien, M. Eren, B. Buchannan, Eds. (MIT Press, 2018), pp. 253–274.

72. R. E. Clopton, Standard nomenclature and metrics of plane shapes for use in Gregarine Taxonomy. *Comp. Parasitol.* **73**, 130–140 (2004).

73. P. D. Polly, L. Killick, M. Ruddy, Using left-right asymmetry to estimate non-genetic variation in vole teeth (*Arvicolinae, Muridae, Rodentia*). *Palaeontol. Electron.* **14**, 1–12 (2011).
